# Targeting *KRAS* Codon 13 Mutations Using Direct Combination Approaches in Non–Small Cell Lung Cancer

**DOI:** 10.1158/2159-8290.CD-25-0884

**Published:** 2026-06-22

**Authors:** William J. McDaid, Helen Adderley, Paolo D. d’Arienzo, Ana S. Parreira, Laura C. Woodhouse, Yongxian Zhuang, Sonia Castillo-Lluva, Kirsten L. Tinsley, James Cooksedge, Martin J. Baker, Joshua Searle, Katherine D. Brown, Mathew Carter, Mihaela Aldea, Arianna Marinello, Jacqueline V. Aredo, Nadia Gomez Serra, Maria Bisbe, Philip East, Sophie de Carné Trécesson, Fabrice Barlesi, Marie Wislez, Benjamin Besse, Heather A. Wakelee, Ernest Nadal, Fiona Blackhall, David C. Wedge, Matthew Holderfield, Jacqueline A.M. Smith, Mallika Singh, Kyle J. Seamon, Angeliki Malliri, Colin R. Lindsay

**Affiliations:** 1 Division of Cancer Sciences, School of Medical Sciences, Faculty of Biology Medicine and Health, The University of Manchester, Manchester, United Kingdom.; 2 Cell Signaling Group, Cancer Research UK Manchester Institute, The University of Manchester, Manchester, United Kingdom.; 3 The Christie NHS Foundation Trust, Manchester, United Kingdom.; 4 Revolution Medicines, Redwood City, California.; 5 Department of Biochemistry and Molecular Biology, Faculty of Chemical Sciences, Complutense University of Madrid, Madrid, Spain.; 6 Cancer Research UK Lung Centre of Excellence, Manchester, United Kingdom.; 7 Department of Cancer Medicine, Gustave Roussy Cancer Campus, Villejuif, France.; 8 Stanford Cancer Institute, Stanford University School of Medicine, Stanford, California.; 9 Catalan Institute of Oncology (ICO), Bellvitge Biomedical Research Institute (IDIBELL), ICO-HUB Comprehensive Cancer Center, L’Hospitalet de Llobregat, Spain.; 10 Clinical Discovery Bioinformatics, Université Paris-Saclay, Institut Gustave Roussy, IHU PRISM, Villejuif, France.; 11 Inflammation and Cancer Plasticity Laboratory, Université Paris-Saclay, Inserm U981, Institut Gustave Roussy, IHU PRISM, Villejuif, France.; 12 Institut Gustave Roussy, Inserm, Prédicteurs Moléculaires et Nouvelles Cibles en Oncologie (U981), Université Paris-Saclay, Villejuif, France.; 13 Department of Pneumology, Thoracic Oncology Unit, AP-HP, Groupe Hospitalier HUPC, Hôpital Cochin, Paris, France.; 14 Centre de Recherche des Cordeliers, UMRS 1138 Complement, Inflammation and Cancer, Université Paris Descartes, Paris, France.

## Abstract

Direct inhibitors of KRAS^G12C^ are now used as standard-of-care therapy in *KRAS*^G12C^-mutant non–small cell lung cancer (NSCLC), but less is understood about the molecular mechanisms and consequent treatment vulnerabilities of *KRAS* codon 13 mutations in this setting. In this study, we characterized the tumorigenic properties of NSCLC harboring *KRAS*^G13X^ in a large multinational cohort of patients with NSCLC and in preclinical models of *KRAS*^G13X^ NSCLC. Induction of KRAS^G13C^ or KRAS^G13D^ expression confers reduced oncopotency compared with allele-specific controls affecting *KRAS* codon 12, particularly KRAS^G13C^. We identified functionally relevant comutations in *KRAS*^G13X^ NSCLC, including *KEAP1*, *STK11*, *BRAF*, and *NF1*. The novel RAS(ON) G13C-selective inhibitor, RMC-8839, reduces viability in a panel of *KRAS*^G13C^ NSCLC cell models. To determine combination partners that enhance RAS(ON) mutant-selective inhibition, a drug repurposing screen revealed that *KRAS*^G13C^ models are selectively vulnerable to chemotherapy. Combination of docetaxel with RMC-8839 demonstrated robust antiproliferative activity in *KRAS*^G13C^-driven NSCLC models *in vitro* and *in vivo*.

**Significance:**

We characterize the second most common *KRAS* mutational hotspot in NSCLC after codon 12, *KRAS*^G13X^, from tumor initiation to advanced disease. *KRAS*^G13C^ is a novel, directly targetable subset of NSCLC, with vulnerability to the combination of chemotherapeutics and the mutant-selective RAS(ON) inhibitor RMC-8839, a finding immediately relevant to patient care.

*See related commentary by Araujo and Skoulidi, p. 2206*

*See related article by Seamon et al., p. 2256*

## Teaser

Clinical and preclinical analyses identify unique features of KRAS codon 13–mutant lung cancer, demonstrating that combining KRAS^G13C^-selective inhibition with chemotherapy potentiates antitumor efficacy.

## Introduction

Point mutation of *RAS* occurs in ∼30% of human cancers (1), most commonly in the isoform *KRAS*, which has been extensively characterized as a critical oncogene in epithelial cancers (2). In non–small cell lung cancer (NSCLC), which accounts for ∼20% of international cancer-related deaths (3, 4), *KRAS* mutations are the most frequently occurring oncogenic driver and are detected in ∼30% of lung adenocarcinoma cases (5). Although recent breakthroughs with KRAS^G12C^ inhibitors offer new treatment options to patients affected by *KRAS*^G12C^-mutant NSCLC, most cancers with *KRAS* mutations are affected by other mutations, for which there are several emerging direct RAS inhibitors, but there are no current clinically approved targeted therapies (6).

*KRAS* mutations affecting codon 13 (hereafter referred to as “*KRAS*^G13X^”) occur in 5% to 7% of *KRAS*-mutant NSCLC cases, representing the second most common mutational hotspot after codon 12 (7). *KRAS*^G13D^ mutations are also relatively common in colorectal cancer (7). *KRAS*^G13X^ mutants confer oncogenicity by diminishing RAS hydrolysis and increasing its intrinsic nucleotide exchange, while also having a propensity, in colorectal cancer, to comutate with *NF1* that results in impaired neurofibromin GTPase-activating protein–mediated RAS-GTP hydrolysis (8, 9). The presence of the *KRAS*^G13D^ mutation in colorectal cancer predicts improved outcomes with upstream receptor tyrosine kinase targeting via EGFR inhibition but also with chemotherapy, when compared with other *KRAS* mutations (10, 11), a finding that may be dependent on the presence of *NF1* comutation (9, 12). To date, *KRAS*^G13X^ mutations have not been directly targetable in cancer. Furthermore, recent reports suggest that *KRAS*^G13X^-driven cancers are less vulnerable to broad-spectrum RAS(ON)-targeted therapy than *KRAS*^G12X^-driven cancers (13, 14), highlighting the need for investigation into mutant allele-targeted approaches.

Beyond targeting non-*KRAS*^G12C^ mutations, another key challenge for RAS precision medicine is the anticipation of resistance, which has already been characterized extensively for KRAS^G12C^ inhibitors in both the preclinical and clinical settings (15–17). This problem is of huge importance given that (i) no (K)RAS-specific targeted therapy has yet been shown to improve overall survival (OS) for patients with NSCLC and (ii) it would take decades and enormous resources to conduct clinical trials that cover the vast spectrum of emerging RAS inhibitor combinations. Refining the selection of patients with NSCLC and treatment combination partners using rational preclinical approaches, therefore, has the potential to offer enormous clinical impact, minimizing patient time wasted on ineffective treatment combinations.

Here, we characterize preclinical models of *KRAS*^G13X^ NSCLC that correspond to different stages of NSCLC tumor evolution in humans, while in parallel detailing patient outcomes and corresponding genetic profiles using international clinical databases of early and advanced *KRAS*^G13X^-mutant and, for comparison, *KRAS*^G12X^-mutant NSCLC. Across all models and datasets, we genetically and functionally validate key pathogenic comutations driving oncogenesis in *KRAS*^G13X^ NSCLC. Applying targeted and empirical approaches to identify and validate novel cell-intrinsic treatments that are specific to *KRAS*^G13X^, we demonstrate genotype-specific therapeutic vulnerabilities of *KRAS*^G13C^ NSCLC to chemotherapy and a novel RAS(ON) G13C-selective tricomplex inhibitor, RMC-8839, both as monotherapies and in combination. In summary, we demonstrate for the first time in a preclinical setting that *KRAS* codon 13 mutations are a directly targetable subset of NSCLC.

## Results

### Examining the Outcomes and Molecular Determinants of PD-1/PD-L1 Immune Checkpoint Blockade in *KRAS*^G13X^ NSCLC

We began by examining the vulnerability of *KRAS*^G13X^ NSCLC to immune checkpoint inhibition (ICI) with PD-1/PD-L1 blockade. This was assessed using databases of early-stage NSCLC [Genomics England National 100,000 Genome Program (100KGP)] and advanced NSCLC [RAS-Precision Medicine (RAS-PM), cBioPortal, and The Cancer Genome Atlas (TCGA) Firehose Legacy database; Supplementary Fig. S1A]. This treatment modality is specifically relevant to the majority of newly diagnosed patients with treatment-naive advanced NSCLC and, more recently, in the perioperative treatment setting (18–21). Previous data suggest that patients affected by *KRAS*^G12D^ NSCLC may not benefit from ICIs (22), but the clinical benefit of PD-1/PD-L1 inhibition has not previously been evaluated in the context of rarer *KRAS*-mutant NSCLC subtypes.

**Figure 1.**
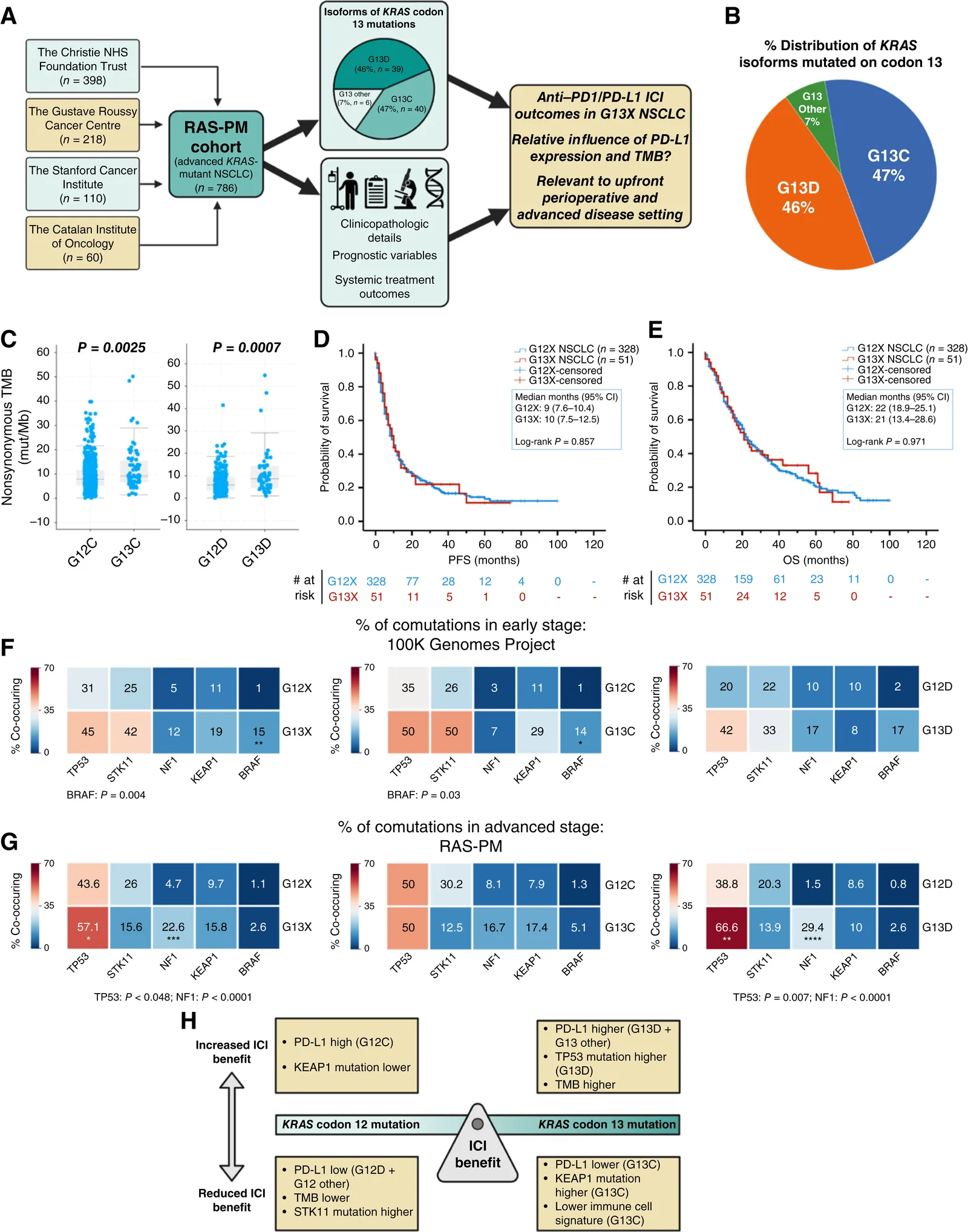
Examining the response and molecular determinants of PD-1 immune checkpoint blockade in *KRAS*^G13X^ NSCLC. **A,** Schematic illustrating the use of RAS-PM to explore ICI-specific outcomes, TMB, and PD-L1 in patients affected by advanced/inoperable *KRAS*-mutant NSCLC. **B,** Percentage of patients within the codon 13 cohort harboring *KRAS*^G13C^, *KRAS*^G13D^, and other codon 13 mutations. **C,** Analysis of TMB between *KRAS*^G12C^ and *KRAS*^G13C^ patients and between *KRAS*^G12D^ and *KRAS*^G13D^ patients from cBioPortal. **D,** PFS between *KRAS* codon 12 and 13 mutant NSCLC patients receiving immunotherapy as first-line treatment. **E,** OS between *KRAS* codon 12 and 13 mutant NSCLC patients receiving immunotherapy first-line treatment. **F,** The percentage of comutations associated with *KRAS*^G12C^-, *KRAS*^G12D^-, *KRAS*^G13C^-, and *KRAS*^G13D^-mutant patients taken from the 100,000 Genomes Project. Comparisons are as follows: *KRAS*^G12X^ (*n* = 155) vs. *KRAS*^G13X^ (*n* = 26), *KRAS*^G12C^ (*n* = 115) vs. *KRAS*^G13C^ (*n* = 14), and *KRAS*^G12D^ (*n* = 40) vs. *KRAS*^G13D^ (*n* = 12). **G,** The percentage of comutations associated with *KRAS*^G12C^-, *KRAS*^G12D^-, *KRAS*^G13C^-, and *KRAS*^G13D^-mutant patients taken from RAS-PM. Comparisons are as follows: *KRAS*^G12X^ vs. *KRAS*^G13X^, *KRAS*^G12C^ vs. *KRAS*^G13C^, and *KRAS*^G12D^ vs. *KRAS*^G13D^. Not every patient was tested for all 5 comutations simultaneously. Refer to Supplementary Table S3 for the numbers of patients tested for each comutation. **H,** Schematic illustrating factors contributing to the lack of difference in immunotherapy response between *KRAS*^G12X^- and *KRAS*^G13X^-driven NSCLC patients from the RAS-PM database. Statistical significance between comparisons was calculated using an unpaired Student *t* test (**C**), log-rank (Mantel–Cox; **D** and **E**), and Fisher exact test (**F** and **G**). ****, *P* < 0.0001; ***, *P* < 0.001; **, *P* < 0.01; *, *P* < 0.05; ns > 0.05. **A,** Created in BioRender. Tinsley, K. (2026) https://BioRender.com/vyu8cs2; **H,** Created in BioRender. Tinsley, K. (2026) https://BioRender.com/4ju5v3u.

To test this question in a large, multicenter, international real-world cohort, we first reviewed ICI-specific outcomes in patients affected by advanced/inoperable *KRAS*-mutant NSCLC, analyzing the RAS-PM patient cohort (Fig. 1A). For this dataset, we curated and examined clinicopathologic details, prognostic variables, and systemic treatment outcomes of 398 patients with *KRAS*-mutant NSCLC treated at The Christie NHS Foundation Trust, 218 patients at the Gustave Roussy Cancer Centre, 110 patients at the Stanford Cancer Institute, and 60 patients at the Catalan Institute of Oncology, totaling 786 patients (Fig. 1A). Of 83 NSCLCs affected by *KRAS* codon 13 mutations, 39 (47%) were *KRAS*^G13C^, 38 (45.7%) were *KRAS*^G13D^, and 6 (7.1%) had other codon 13 mutations (Fig. 1B; Supplementary Fig. S1B). Baseline characteristics of patients affected by advanced NSCLC harboring codon 13 mutations were mostly comparable with those harboring the more common *KRAS* codon 12 mutations. We observed that PD-L1 expression by IHC [tumor proportion score (TPS)], the most commonly used clinical biomarker of ICI response (23, 24), was numerically higher in patients with *KRAS*^G13X^ NSCLC (median PD-L1 TPS%: *KRAS*^G12X^ = 19% and *KRAS*^G13X^ = 30%; Supplementary Fig. S1C). Based on the specific *KRAS* mutation, median PD-L1 expression was highest in *KRAS*^G13D^ NSCLC but lowest in *KRAS*^G13C^ NSCLC (median PD-L1 TPS%: *KRAS*^G12C^ = 39%, *KRAS*^G12D^ = 9%, *KRAS*^G12other^ = 5%, *KRAS*^G13C^ = 7.5%, *KRAS*^G13D^ = 45%, and *KRAS*^G13other^ = 72.5%; Supplementary Fig. S1D). Tumor mutational burden (TMB), a second clinically used biomarker of ICI response (25), was assessed in a limited number of RAS-PM patients, revealing a higher burden for *KRAS*^G13X^ patients (median TMB for *KRAS*^G12C^ vs. *KRAS*^G13C^: 12 vs. 13.7 mut/Mb; *KRAS*^G12D^ vs. *KRAS*^G13D^: 4.6 vs. 10 mut/Mb; Supplementary Table S1). Consistent with this, cBioPortal (26–28) also showed that TMB was higher in *KRAS*^G13X^ (median TMB for *KRAS*^G12C^ vs. *KRAS*^G13C^: 7.83 vs. 9.18 mut/Mb; *KRAS*^G12D^ vs. *KRAS*^G13D^: 5.87 vs. 8.65 mut/Mb; Fig. 1C). Despite these increases in PD-L1 expression and TMB in *KRAS*^G13X^ NSCLC compared with *KRAS*^G12X^ NSCLC, there was no significant difference in progression-free survival (PFS) or OS between these two groups when receiving ICI-based treatments in the first-line advanced setting (PFS: median, 9 months for *KRAS*^G12X^ patients vs. 10 months for *KRAS*^G13X^ patients; *P* = 0.857, log-rank; OS: median, 22 months for *KRAS*^G12X^ patients vs. 21 months for *KRAS*^G13X^ patients, *P* = 0.971, log-rank; Fig. 1D and E; Supplementary Table S2).

We next considered that comutations associated with immunotherapy vulnerability may contribute to rates of ICI response in *KRAS*^G13X^ patients. Aiming to address this question comprehensively in both early- and late-stage NSCLC (Supplementary Fig. S1A), we first analyzed a cohort of resected early-stage *KRAS*-mutant NSCLC tumors, which underwent whole-genome sequencing (WGS) via the Genomics England National 100KGP. This national initiative represents the largest reported cancer cohort of whole genomes internationally, including a 20-fold increase in primary lung tumor specimens compared with previous WGS studies. In total, 677 resected lung adenocarcinomas underwent WGS, of which 115, 41, 14, and 12 harbored *KRAS*^G12C^, *KRAS*^G12D^, *KRAS*^G13C^, and *KRAS*^G13D^ mutations, respectively (Supplementary Fig. S1E). Comparing codon 13 lung adenocarcinomas with those harboring *KRAS* codon 12 mutations, we noted that there was a higher propensity for common comutations to occur in the codon 13 group (Fig. 1F), including mutations affecting *TP53*, *KEAP1*, and *STK11*, that are associated with modulation of ICI benefit (29–31). Mutations in *KEAP1* and *STK11* have been reported to promote an immunosuppressive environment, which includes increased myeloid cells and decreased CD8^+^ T cells (32). This comutational association was particularly evident in *KRAS*^G13C^ tumors, which were more likely to comutate with variants of *TP53*, *STK11*, and *KEAP1* in the early-stage 100KGP cohort. *TP53* and *STK11* mutations were also very prevalent in *KRAS*^G13D^ tumors. Of genes more commonly associated with cancer cell intrinsic changes to the mitogen-activated protein kinase (MAPK) pathway, *BRAF* variants were more likely to occur in the presence of codon 13 tumors, a finding that was statistically significant despite the relatively low codon 13 tumor numbers available for analysis (Fig. 1F).

To complement the early-stage 100KGP cohort, we also investigated comutational differences between *KRAS*^G12X^ and *KRAS*^G13X^ in advanced NSCLC using the RAS-PM database (Fig. 1G; Supplementary Table S3). Consistent with the early-stage cohort, *TP53* comutations were more prevalent in NSCLC with *KRAS* codon 13 mutations (Fig. 1G). This observation was specific to *KRAS*^G13D^, as *KRAS*^G13D^ NSCLC had a higher *TP53* comutation prevalence compared with *KRAS*^G12D^, whereas *KRAS*^G13C^ and *KRAS*^G12C^ had comparable *TP53* prevalence. In contrast to early-stage NSCLC, despite low numbers for *KRAS*^G13X^ tumors precluding statistical confirmation, a higher *STK11* comutational prevalence was observed in cancers with *KRAS* codon 12 variants (Fig. 1G). A trend for increased *KEAP1* mutation prevalence was particularly evident in cancers with *KRAS*^G13C^, a finding comparable with the orthogonal early-stage 100KGP cohort. High *NF1* comutational prevalence was notably more prevalent in advanced NSCLC affected by *KRAS*^G13C^ and *KRAS*^G13D^ (particularly *KRAS*^G13D^), implying that *NF1* alterations are acquired in later stages of tumor progression (Fig. 1G). Although less apparent in advanced patients with NSCLC, *BRAF* mutations were more likely to occur in patients with *KRAS*^G13X^ NSCLC (Fig. 1G). In addition, we found *KRAS*^G13X^ tumors harbored a significantly higher number of co-occurring *KRAS* mutations (*KRAS*^G12X^: 1.1%; *KRAS*^G13X^: 6.7%, *P* = 0.002; Supplementary Fig. S1F). Overall, comparing *KRAS* codon 12 with codon 13 NSCLC across early-stage (100KGP) and advanced-stage (RAS-PM) cohorts, *KRAS*^G13D^ NSCLC comutated persistently across tumor evolution with *TP53*, and *KRAS*^G13C^ NSCLC comutated persistently with *KEAP1*. *NF1* comutations became more prevalent with *KRAS*^G13X^ tumor progression compared with *KRAS*^G12X^ tumors (Fig. 1F and G).

Using the RAS-PM cohort to validate *KRAS*^G13X^ ICI survival outcomes in the context of *TP53*, *STK11*, and *KEAP1* mutations, we examined the clinical outcomes of *KRAS*^G13X^ patients harboring either a *TP53* mutation alone (*G13X/TP53*) versus patients harboring *STK11* and/or *KEAP1* mutations (*G13X/STK11* and/or *KEAP1*). The *G13X/TP53* subgroup had significantly higher PD-L1 TPS compared with the *G13X/STK11* and/or *KEAP1* subgroup (median PD-L1 TPS%: *G13X/TP53* alone = 60%; *G13X/STK11* and/or *KEAP1* = 0%, *P* = 0.043; Supplementary Fig. S2A), which translated into longer PFS and OS (statistically significant; OS: median 23 months vs. 7 months, *P* = 0.027, log-rank; Supplementary Fig. S2B and S2C). This is supported by previous NSCLC reports (29–31) and also highlights that these comutation-influenced ICI responses are relevant to rarer *KRAS*-mutant subtypes, which up until now have been underreported.

To corroborate our treatment findings, we assessed immune gene signatures using RNA-seq data from the TCGA Firehose Legacy dataset (33). Examining the expression of genes, which define immune cell populations, we observed comparable expression of immune cell signatures between *KRAS*^G12D^ and *KRAS*^G13D^ patient tumors (Supplementary Fig. S3A). However, reduced expression of immune cell genes was clearly evident in *KRAS*^G13C^ patient tumors compared with *KRAS*^G12C^ tumors (Supplementary Fig. S3B). In particular, we observed reduced signature expression of CD45^+^ cells, CD8^+^ and CD4^+^ T cells, dendritic cells, macrophages, and mast cells, suggesting that *KRAS*^G13C^ tumors are immune cell–excluded and less responsive to immunotherapy.

Summarizing these clinical datasets, we observed no difference in ICI survival outcomes between patients with *KRAS*^G12X^ NSCLC and those with *KRAS*^G13X^ NSCLC, despite noting higher TMB, PD-L1 TPS, and *TP53* comutational prevalence in *KRAS*^G13D^ tumors (Fig. 1H). However, *KRAS*^G13C^ NSCLCs were noted to have a higher prevalence of *KEAP1* comutation, lower PD-L1 TPS, and a more immune-excluded TME (Fig. 1H). These findings highlighted the importance of investigation into alternative treatment modalities for patients who harbor this specific *KRAS* mutation.

### *KRAS* Codon 13 Mutations Confer Reduced Oncopotency during Tumor Initiation

**Figure 2.**
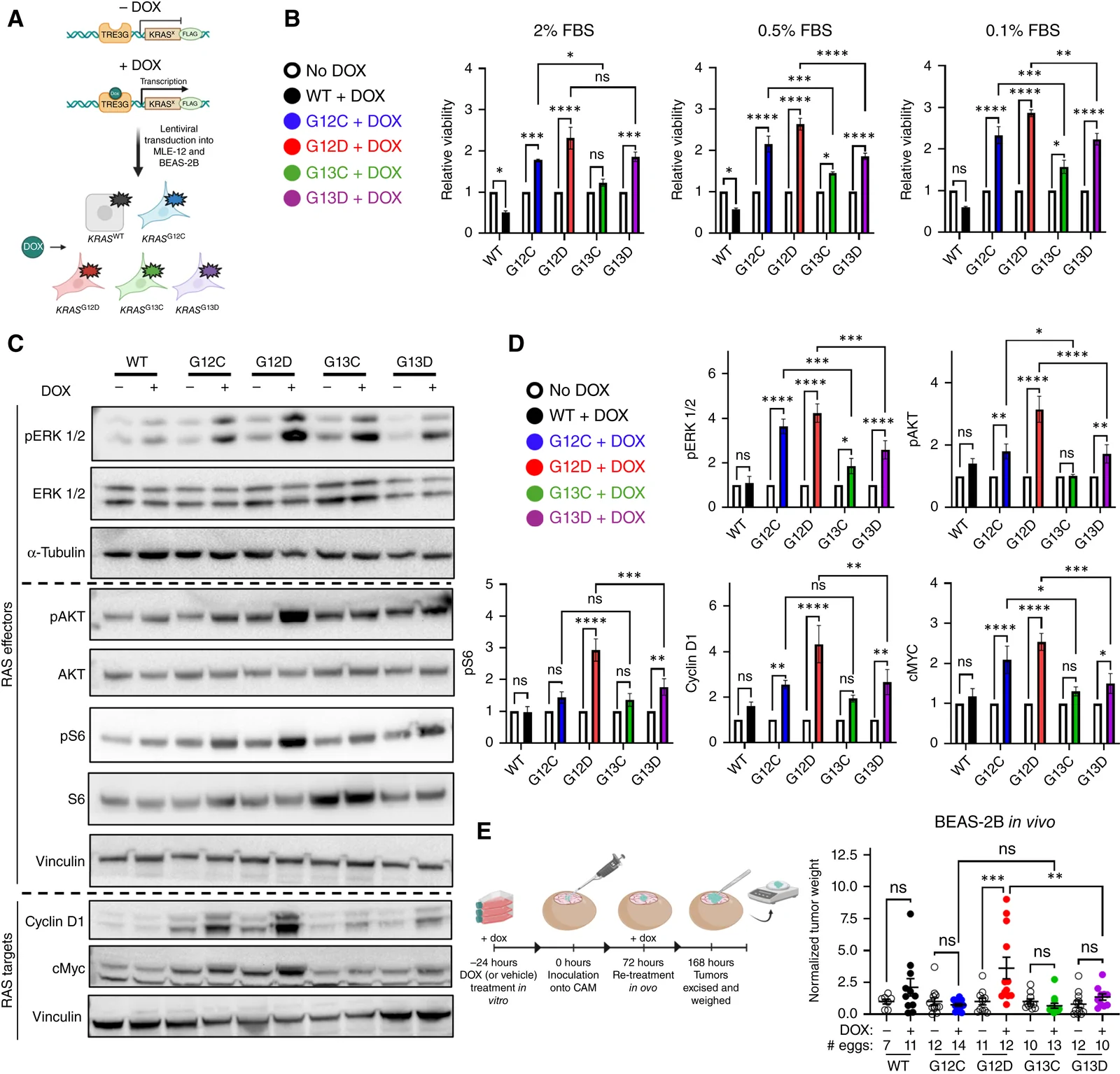
*KRAS* codon 13 mutations confer reduced oncopotency. **A,** Schematic outlining the generation of DOX-inducible isogenic MLE-12 and BEAS-2B cells to compare the effect of expressing different mutant KRAS variants on NSCLC initiation. **B,** Relative viability of isogenic MLE-12 cells 96 hours after DOX-induced expression (100 ng/mL) of each mutant KRAS variant in three different serum conditions – 2%, 0.5%, and 0.1% FBS. Cells were plated in ULA conditions. Viability was analyzed using CellTiter-Glo 3D. Data for each isogenic line are expressed relative to the untreated (no DOX) control of that particular line. *n* = 3 biological repeats; mean ± SEM. **C,** Western blotting of isogenic MLE-12 cells 24 hours after DOX-induced expression (100 ng/mL) of each mutant KRAS variant in 0.5% serum. Cells were plated in ULA conditions. Data are representative of five biological repeats for KRAS effector proteins and four biological repeats for KRAS target proteins. Dashed lines indicate that representative blots from three different biological replicates are presented. Vinculin and α-tubulin were used as protein loading controls. **D,** Quantification of **C** and associated replicates. For phospho-proteins (KRAS effector blots), phosphorylated protein and total protein run on separate blots were normalized to their respective loading controls on the same blot, and then phospho-protein was normalized to the total protein. Data for each isogenic line were then normalized to the untreated (no DOX) control of that particular line. KRAS target proteins were normalized to the loading control, and then the data were normalized to the untreated (no DOX) control. *n* = 5 biological repeats for KRAS effector proteins and four biological repeats for KRAS target proteins; mean ± SEM. **E,** (Left) Timeline of BEAS-2B oncogenicity *in vivo* experiment using chicken egg xenograft. (Right) Normalized tumor weights measured upon termination of the experiment. Number of eggs indicated per condition. Mean ± SEM reported. Statistical significance between comparisons was calculated using a two-way ANOVA (**B**, **D**, and **E**). ****, *P* < 0.0001; ***, *P* < 0.001; **, *P* < 0.01; *, *P* < 0.05; ns, > 0.05. **A,** Created in BioRender. Tinsley, K. (2026) https://BioRender.com/ojsukx1; **E,** (Left) Created in BioRender. Tinsley, K. (2026) https://BioRender.com/wrfehon.

We recently showed that *KRAS*^G12D^ is more “oncopotent” than *KRAS*^G12C^ in driving NSCLC initiation, a phenotype conferred by coopting the PI3K–AKT–mTOR signaling pathway (34). However, little is known about the tumorigenic properties of codon 13 *KRAS* mutations in this disease. Given the higher abundance of comutations within *KRAS*^G13X^-driven NSCLCs relative to *KRAS*^G12X^-driven NSCLCs (Fig. 1F and G), we hypothesized that *KRAS*^G13X^ mutations may confer a weaker oncogenic phenotype compared with the more common codon 12 variants in NSCLC. To examine and compare the oncogenic potential of *KRAS* codon 13 and codon 12 variants in isolation in NSCLC, we generated an isogenic panel using an immortalized murine alveolar type 2 cell line, MLE-12 cells, to ectopically express FLAG-tagged wild-type (WT) KRAS (KRAS^WT^), KRAS^G12C^, KRAS^G12D^, KRAS^G13C^, or KRAS^G13D^ using a doxycycline (DOX)-regulated system (Fig. 2A; Supplementary Fig. S4A). Culturing MLE-12 cells in ultralow attachment (ULA) plates to induce growth as spheroids, we first confirmed in 3D that the induction of KRAS^G13X^ expression by DOX can induce their transformation, using CD44 as a readout (Supplementary Fig. S4B; ref. 35). In MLE-12 cells grown in 2% FBS-containing medium, a significant increase in cell viability was observed at 96 hours after DOX treatment in KRAS^G12C^, KRAS^G12D^, and KRAS^G13D^-mutant initiated cells when compared with untreated controls, with KRAS^G12D^ expression driving the greatest increase in viability (Fig. 2B). No significant increase in viability was observed for cells expressing KRAS^G13C^. When comparing the specific amino acid substitutions between codons directly, MLE-12 cells expressing KRAS^G13C^ had reduced viability compared with MLE-12 cells expressing KRAS^G12C^, and similarly, KRAS^G13D^ expression also led to reduced viability compared with KRAS^G12D^ expression. As growth factors in serum can activate proliferation pathways through endogenous WT RAS, thereby reducing the impact of exogenous KRAS-mutant expression, we repeated this experiment in lower (0.5% FBS) and restricted (0.1% FBS) serum conditions to minimize endogenous RAS activation. All four KRAS mutants had a much stronger impact on viability upon expression under reduced serum conditions. More importantly, the same allele-specific trend of *KRAS*^G13X^ mutations being less oncopotent than *KRAS*^G12X^ mutations was noted, with statistically significant differences seen in lower serum conditions (Fig. 2B). Taken together, these results are indicative of weaker oncopotency when *KRAS* is mutated at codon 13, with *KRAS*^G13C^ being the weakest oncogene (Fig. 2B). In parallel with these phenotypic changes, we investigated the activation of KRAS signaling effectors in medium containing 0.5% serum, in which significant differences in viability between codons were evident. As we have previously reported (34), we observed that MAPK signaling [phospho-ERK 1/2 (pERK 1/2)] was comparable between *KRAS*^G12C^ and *KRAS*^G12D^ MLE-12 cells, whereas PI3K–AKT–mTOR signaling [phospho-AKT (pAKT) and phospho-S6 (pS6)] was more activated in *KRAS*^G12D^ MLE-12 cells (Fig. 2C and D). *KRAS*^G13X^ MLE-12 cells were associated with significantly weaker MAPK and PI3K–AKT–mTOR signaling outputs compared with *KRAS*^G12X^ MLE-12 cells, suggesting that *KRAS*^G13X^ mutations are less potent activators of KRAS signaling (Fig. 2C and D). We also examined RAS signaling gene expression targets such as cyclin D1 (36) and cMyc (37), observing lower expression of these targets in *KRAS*^G13X^ MLE-12 cells compared with *KRAS*^G12X^ cells (Fig. 2C and D). To support the reproducibility and robustness of these findings, we generated a second isogenic panel using an immortalized human bronchial cell line, BEAS-2B, with ectopic expression of FLAG-tagged KRAS^WT^ and the KRAS mutants mentioned above (Supplementary Fig. S4C). Similar to the MLE-12 model, mutant expression induced cell transformation, as indicated by mutant-specific increases in CD44 (Supplementary Fig. S4D). An increase in cell viability and spheroid area was observed over three different time points in *KRAS*-mutant expressing cells when compared with cells expressing KRAS^WT^ (Supplementary Fig. S4E). Supporting the MLE-12 data, BEAS-2B cells expressing KRAS^G13C^ or KRAS^G13D^ exhibited reduced viability and spheroid growth compared with cells with KRAS^G12C^ or KRAS^G12D^ expression, respectively (Supplementary Fig. S4E). Furthermore, there was a trend for KRAS^G13X^-expressing BEAS-2B cells to exhibit weaker KRAS signaling: pERK 1/2, pAKT, pS6, and p4E-BP1 [a second mTOR effector (38)] were less activated in *KRAS*^G13X^ BEAS-2B cells compared with *KRAS*^G12X^ BEAS-2B cells (Supplementary Fig. S4F and S4G). In parallel, using an isogenic *KRAS*-mutant mouse embryonic fibroblast (MEF) model (39), we saw that *KRAS*^G12D^ MEFs were able to grow as spheroids, whereas *KRAS*^G13D^ MEFs could not (Supplementary Fig. S4H). Similar to the initiation models, there was reduced pERK 1/2 and pS6 in *KRAS*^G13D^ MEFs compared with *KRAS*^G12D^ MEFs (Supplementary Fig. S4I).

Finally, we assessed *KRAS*^G13X^*in vivo* tumorigenicity by inoculating the isogenic BEAS-2B models onto the chorioallantoic membrane (CAM) of 11-day-old chicken eggs. Cells were exposed to DOX *in vitro* 24 hours before inoculation onto the CAM. Seventy-two hours later, cells were exposed again to DOX to maintain mutant KRAS expression. After a further 96 hours, tumor weight was measured as a readout for *in vivo* growth (Fig. 2E). A significant increase in tumor weight was observed in KRAS^G12D^-expressing BEAS-2B cells when compared with the respective untreated control and KRAS^G13D^-expressing BEAS-2B cells. There was no increase in tumor weight observed in BEAS-2B cells expressing the other KRAS mutants, indicating weaker oncogenic potential *in vivo* (Fig. 2E). This was a short-term experiment, and it is possible that longer time points could aid in evaluating differences between *KRAS*^G12C^ and *KRAS*^G13C^-driven *in vivo* oncogenicity. However, in a previous report utilizing a barcoded library of *KRAS* mutations to initiate lung tumors, it was observed that most *KRAS*^G13X^ mutants initiated less tumors compared with most *KRAS*^G12X^ mutants, consistent with our findings (40).

Overall, these results indicate that *KRAS*^G13X^ mutations confer reduced oncopotency in NSCLC models during tumor initiation and are associated with lower KRAS signaling activation.

### Comutations Converge on the RAS Pathway to Increase *KRAS* Codon 13 Oncogenicity

**Figure 3.**
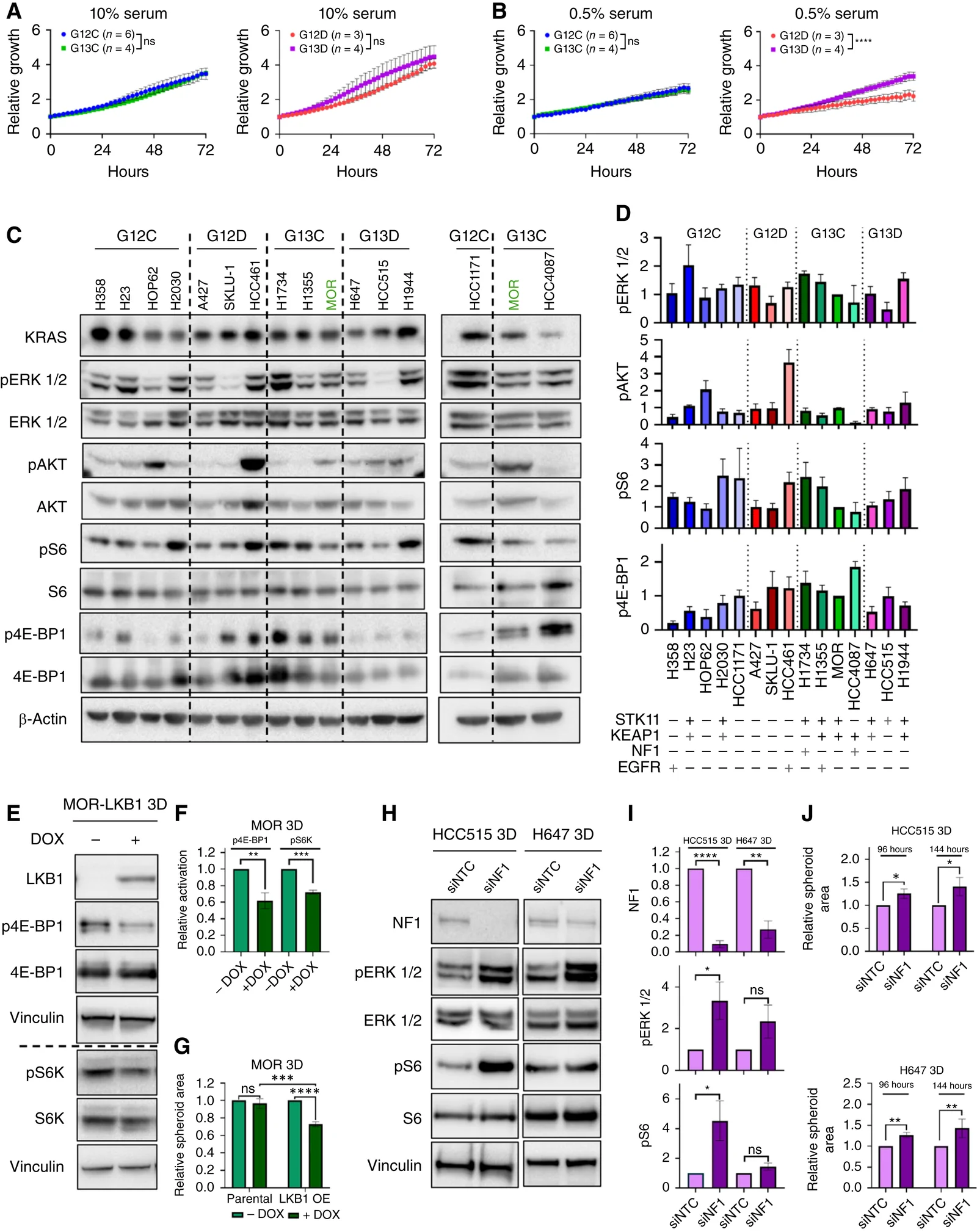
Comutations converge on the RAS pathway to increase *KRAS* codon 13 oncogenicity. **A,** Relative proliferation rates of advanced NSCLC cell lines cultured in medium containing 10% FBS. Cell lines are grouped and averaged by mutant *KRAS* genotype, with comparisons made between (left) *KRAS*^G12C^ and *KRAS*^G13C^ cell lines and (right) *KRAS*^G12D^ and *KRAS*^G13D^ cell lines. Each dot represents a time point, which is normalized to 0 hours. *n* = 3–6 cell lines per genotype, with three biological repeats carried out for each cell line; mean ± SEM. **B,** Relative proliferation rates of advanced NSCLC cell lines cultured in medium containing 0.5% FBS. Cell lines are grouped and averaged by mutant *KRAS* genotype, with comparisons made between (left) *KRAS*^G12C^ and *KRAS*^G13C^ cell lines and (right) *KRAS*^G12D^ and *KRAS*^G13D^ cell lines. Each dot represents a time point, which is normalized to 0 hours. *n* = 3–6 cell lines per genotype, with three biological repeats carried out for each cell line; mean ± SEM. **C,** Western blotting of advanced cancer cell lines 48 hours after plating in 3D ULA conditions in medium containing 10% FBS, seeded at equal cell density. Cell lines were run on two gels, with MOR cells run on both to allow quantification and comparison between both blots. Data are representative of 2–4 biological repeats. β-Actin was used as a protein loading control (sample processing control). Dashed lines separate cell lines belonging to each *KRAS*-mutant genotype. **D,** Quantification of **C**. Phosphorylated protein and total protein run on separate blots were normalized to the sample processing control, and then phospho-protein was normalized to the total protein. The data were then normalized to MOR cells to allow comparisons between blots. *n* = 2–4 biological repeats per cell line; mean ± SEM. The comutations associated with each cell line are listed underneath. Black + indicates pathogenic alteration, gray + indicates a variant of unknown significance, and – indicates no alteration. **E,** Western blotting of MOR-LKB1 overexpressing (OE) cells 24 hours after DOX-induced expression (1,000 ng/mL) of LKB1. Cells were plated in 3D ULA conditions. Data are representative of 3–4 biological repeats for every protein probed. Vinculin was used as a protein loading control. The dashed line indicates that representative blots from two different biological replicates are presented. **F,** Quantification of **E**. Phosphorylated protein and total protein run on separate blots were normalized to their respective loading controls on the same blot, and then phospho-protein was normalized to the total protein. The data were then normalized to untreated (no DOX) control. *n* = 3–4 biological repeats for every protein probed; mean ± SEM. **G,** Relative spheroid area of MOR parental and MOR-LKB1 OE cells 8 days after DOX-induced expression (1,000 ng/mL) of LKB1. Cells were plated in ULA conditions. Spheroid area was analyzed using ImageJ. Data are expressed relative to untreated (no DOX) control. *n* = 3 biological repeats; mean ± SEM. **H,** Western blotting of HCC515 and H647 cells 72 hours after exposure to a cocktail of four siRNAs targeting *NF1* (siNF1) pooled together or nontargeting siRNA (siNTC). Data are representative of 3 or 4 biological repeats. Vinculin was used as a protein loading control. **I,** Quantification of **H**. Phosphorylated protein and total protein run on separate blots were normalized to their respective loading controls on the same blot, and then phospho-protein was normalized to the total protein. Data within each cell line were then normalized to siNTC control. *n* = 3 or 4 biological repeats; mean ± SEM. **J,** Relative spheroid area of HCC515 and H647 cells 96 and 144 hours after exposure to a cocktail of four siRNAs targeting *NF1* (siNF1) pooled together or nontargeting siRNA (siNTC). Cells were plated in ULA conditions. Spheroid area was analyzed using ImageJ. Data within each cell line are expressed relative to siNTC. *n* = 3 biological repeats per cell line; mean ± SEM. Statistical significance between comparisons was calculated using a two-way ANOVA (**A**, **B**, and **G**) and an unpaired Student *t* test (**F**, **I**, and **J**) and ****, *P* < 0.0001; ***, *P* < 0.001; **, *P* < 0.01; *, *P* < 0.05; ns > 0.05.

Having established the weaker oncopotency of KRAS^G13X^ oncoproteins during tumor initiation, we next determined if these phenotypes persisted in advanced tumor cell models. We utilized an NSCLC cell line panel, which covered the four *KRAS* mutations tested in the initiation models: *KRAS*^G12C^, *KRAS*^G12D^, *KRAS*^G13C^, and *KRAS*^G13D^. These cell lines also harbored other genetic alterations commonly seen in NSCLC, such as *TP53*, *STK11*, *KEAP1*, *EGFR*, *CDKN2A*, *ATM*, and *NF1* (Supplementary Fig. S5A). First, when assessing the proliferation of advanced NSCLC cell lines in 10% FBS, our findings contrasted with the initiation models—we could no longer see codon-specific differences in proliferation, with NSCLC cell lines proliferating at comparable rates when they were grouped by *KRAS*-mutant genotype (Fig. 3A; Supplementary Fig. S5B). Given that these codon-specific differences in proliferation were more striking between *KRAS*^G12X^- and *KRAS*^G13X^-driven initiation models in reduced serum conditions (0.5% and 0.1% FBS), we also assessed the proliferation of NSCLC cell lines in serum-restricted conditions (0.5% FBS). Although the majority of cell lines grew slower in serum-restricted conditions (Supplementary Fig. S5C), the impact was most pronounced in NSCLC cell lines expressing KRAS^G12D^, with a mean growth inhibition of between 39.2% and 58.3% for *KRAS*^G12D^ NSCLC cell lines compared with 10.9% and 40.7%, 24.9% and 38.1%, and 29.6% and 33.9% for *KRAS*^G12C^, *KRAS*^G13C^, and *KRAS*^G13D^ NSCLC lines, respectively (HCC1974 cells were the only exception and proliferated better in restricted serum conditions). Therefore, when cell lines were grouped according to *KRAS* genotype, those expressing *KRAS*^G13C^ grew at comparable rates to those driven by *KRAS*^G12C^ cell lines in restricted serum (Fig. 3B; Supplementary Fig. S5D), yet *KRAS*I-mutated cells grew slightly faster than *KRAS*^G12D^-mutated lines when grown in 0.5% serum (Fig. 3B; Supplementary Fig. S5D). Taken together, these data contrast with our isogenic initiation models and suggest that in advanced disease, *KRAS*^G13X^-driven NSCLC models no longer demonstrate a weaker oncogenic phenotype when compared with *KRAS*^G12X^ models, implying that *KRAS*^G13X^ oncogenicity increases during disease progression.

Given that our comutation analyses within the 100KGP and RAS-PM datasets revealed the presence of key comutations, which converge on RAS signaling pathways (*NF1*, *STK11*, *KEAP1*, and *BRAF*; Fig. 1F and G), as well as a higher incidence of secondary *KRAS* mutations (Supplementary Fig. S1F) in *KRAS*^G13X^ NSCLC, we hypothesized that these alterations upregulate RAS signaling in *KRAS*^G13X^-expressing cells, increasing oncogenicity and fitness under stress conditions. In our cell line panel, *KRAS*^G13X^ cell lines mostly harbored alterations in *STK11* and *KEAP1* (and alterations of unknown significance in *EGFR* and *NF1*), known to enhance MAPK and mTORC1 signaling (9, 41–43). Examining signaling downstream of KRAS in our advanced NSCLC panel, there was comparable pERK 1/2 activation between cell lines of different *KRAS*-mutant genotypes (Fig. 3C and D; Supplementary Fig. S6), contrasting with our initiation models and suggesting an increase in MAPK signaling in *KRAS*^G13X^-driven NSCLC cell lines compared with what was observed at the point of initiation. Additionally, pAKT and pS6 were also comparable across the four genotypes, again contrasting with *KRAS*^G13X^-initiated cells in which lower activation of PI3K–AKT–mTOR signaling was evident (Fig. 3C and D; Supplementary Fig. S6). Notably, in *KRAS*^G13C^-driven NSCLC cell lines, p4E-BP1, which is a direct mTOR substrate, was significantly higher than in those cell lines with *KRAS*^G12C^ (Fig. 3C and D; Supplementary Fig. S6), in contrast to initiation data in which the induction of KRAS^G13C^ expression did not affect the activation of 4E-BP1 after DOX exposure (Supplementary Fig. S4F and S4G).

To confirm that *STK11* mutations enhanced mTOR signaling and *KRAS*^G13C^ oncogenicity, we inducibly expressed LKB1 in KRAS^G13C^-expressing MOR cells (which have lost LKB1 expression through *STK11* alterations), then assessed 4E-BP1 and S6K [a second direct mTOR substrate (44)] activation as a readout of mTOR pathway activity. LKB1 expression led to decreased phosphorylation of S6K and 4E-BP1 (Fig. 3E and F), indicating partial mTOR signaling inhibition. This was accompanied by reduced MOR spheroid area (Fig. 3G), suggesting suppressed anchorage-independent expansion that is consistent with previous reports of LKB1 restoration in *KRAS*-mutant NSCLC cell lines in 3D contexts (45). Given the increase in *NF1* comutational prevalence between late- and early-stage NSCLC, we hypothesized that additional genetic hits to the MAPK pathway further enhanced the tumorigenic potential of *KRAS*^G13X^ NSCLC in advanced disease. As a key MAPK comutation that has also been highlighted in colorectal cancer and suggested to influence treatment vulnerability (9), we validated the functional relevance of *NF1* comutation through genetic depletion. Using small interfering RNA (siRNA) against *NF1*, we observed increased activation of RAS signaling, evidenced by increases in both ERK 1/2 and S6 activation in two *KRAS*^G13D^ NSCLC cell lines, with statistically significant differences seen for HCC515, but not for H647 (Fig. 3H and I). Despite this, both cell lines demonstrated increased proliferation when grown as spheroids across two time points (Fig. 3J), supporting the oncogenic contribution of *NF1* loss-of-function mutations in NSCLC.

Overall, these findings indicate that in advanced NSCLC models, differences in oncopotency between *KRAS*^G12X^ and *KRAS*^G13X^ initiation are no longer evident. Proliferation differences seem to balance between *KRAS*^G12X^- and *KRAS*^G13X^-driven advanced cell lines, which are associated with enhanced engagement of MAPK and mTORC1 signaling in *KRAS*^G13X^-driven lines when compared with tumor initiation models, most notably in NSCLC cancer cell lines expressing KRAS^G13C^. This is, at least in part, due to genetic alterations in proteins that promote RAS pathway signaling through MAPK and PI3K–AKT–mTOR arms and enhance *KRAS*^G13X^ oncogenicity. Furthermore, these alterations, particularly in *STK11* and *NF1*, are known to maintain proliferation under stress conditions such as nutrient and serum deprivation (46, 47), conferring advanced *KRAS*^G13X^ NSCLC models with a survival advantage over NSCLC models driven by other *KRAS* genotypes not dependent on comutations, such as *KRAS*^G12D^.

### Examining a Novel RAS(ON) G13C-Selective Inhibitor, RMC-8839

For patients with pretreated NSCLC (relapsed or second-line advanced), cancer cell–intrinsic approaches offer increasing importance, given that patients are typically treated with immune checkpoint blockade at earlier points in their care (48). Therefore, we next investigated direct targeting of *KRAS* codon 13 mutations using two RAS(ON) tricomplex inhibitors that target the GTP-bound or active (ON) state of KRAS^G13C^. These include the RAS(ON) G13C-selective inhibitor, RMC-8839, and the RAS(ON) multiselective preclinical tool compound, RMC-7977, that is representative of the investigational agent daraxonrasib (RMC-6236; ref. 13). The potential of direct RAS(ON) inhibitors to offer benefits to patients with NSCLC is under clinical trial evaluation using the investigational agents daraxonrasib, elironrasib, and zoldonrasib (NCT06881784).

We first investigated the impact of *KRAS* gene deletion in *KRAS*^G13X^ cell lines relative to *KRAS*^G12X^ using CRISPR-Cas9 genetic screening data from the Cancer Dependency Map (DepMap) database, predicting the responsiveness of *KRAS*^G13X^ cell lines to *KRAS* gene depletion. A mean gene effect of −1 or less signifies that cell lines are dependent on the *KRAS* gene. The mean *KRAS* gene effect was approximately −1.6 for *KRAS*^G12C/G12D^ NSCLC cell lines and approximately −1 for *KRAS*^G13C/G13D^ NSCLC cell lines (Supplementary Fig. S7A), demonstrating that NSCLC cell lines harboring *KRAS*^G12C/G12D^ mutations were more sensitive to KRAS depletion than those harboring *KRAS*^G13C/G13D^ mutations. This implied that *KRAS*^G13X^ NSCLC cell lines can function more independently of KRAS signaling compared with *KRAS*^G12X^-driven cell lines, in turn suggesting they may exhibit reduced sensitivity to pharmacologic KRAS targeting.

**Figure 4.**
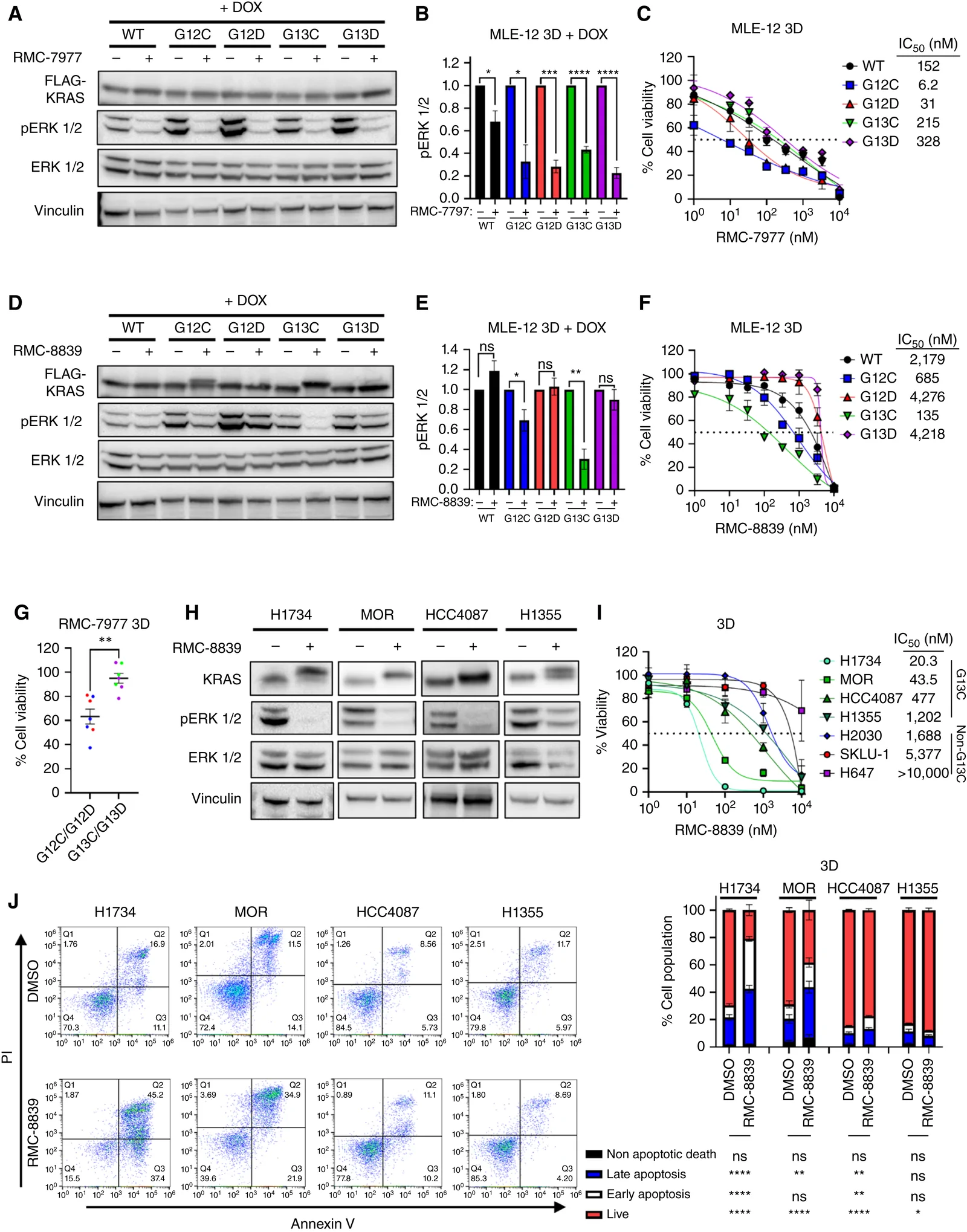
Examining a novel RAS(ON)-G13C selective inhibitor, RMC-8839. **A,** Western blotting of isogenic MLE-12 cells previously treated with DOX for 24 hours to induce KRAS variant expression in 0.5% serum followed by 6 hours of treatment with 50 nmol/L RMC-7977. Cells were plated in ULA conditions. Data are representative of three biological repeats. Vinculin was used as a protein loading control. **B,** Quantification of pERK 1/2 from **A**. Phosphorylated ERK 1/2 and total ERK 1/2 run on separate blots were normalized to their respective loading controls on the same blot, and then pERK 1/2 was normalized to total ERK 1/2. The data for each isogenic line were then normalized to the DMSO control of that line. *n* = 3 biological repeats per isogenic cell line; mean ± SEM. **C,** Dose-response analysis of isogenic MLE-12 cells previously treated with DOX for 24 hours to induce KRAS variant expression in 0.5% serum, followed by 72 hours of treatment with a concentration range of RMC-7977. Cells were plated in ULA conditions. Viability was analyzed by CellTiter-Glo 3D. Data are expressed as a % of DMSO control. *n* = 3 biological repeats per isogenic cell line; mean ± SEM. IC_50_ values were calculated for each cell line and presented as a table. **D,** Western blotting of isogenic MLE-12 cells previously treated with DOX for 24 hours to induce KRAS variant expression in 0.5% serum, followed by 6 hours of treatment with 50 nmol/L RMC-8839. Cells were plated in ULA conditions. Data are representative of three biological repeats. Vinculin was used as a protein loading control. **E,** Quantification of pERK 1/2 from **D**. Phosphorylated ERK 1/2 and total ERK 1/2 run on separate blots were normalized to their respective loading controls on the same blot, and then pERK 1/2 was normalized to total ERK 1/2. The data for each isogenic line were then normalized to the DMSO control of that line. *n* = 3 biological repeats per isogenic cell line; mean ± SEM. **F,** Dose-response analysis of isogenic MLE-12 cells previously treated with DOX for 24 hours to induce KRAS variant expression in 0.5% serum followed by a 72-hour treatment with a concentration range of RMC-8839. Cells were plated in ULA conditions. Viability was analyzed by CellTiter-Glo 3D. Data are expressed as a percentage of DMSO control. *n* = 3 biological replicates per isogenic cell line; mean ± SEM. IC_50_ values were calculated for each cell line and presented as a table. **G,** Viability in response to 10 nmol/L RMC-7977 in advanced *KRAS*-mutant NSCLC cell lines grouped by *KRAS* codon genotype. Cells were plated in ULA conditions. Viability was analyzed after 72 hours by CellTiter-Glo 3D. Each dot represents a cell line within that genotype. Data are expressed as a percentage of DMSO control. *n* = 3 biological repeats per cell line; mean ± SEM. **H,** Western blotting of *KRAS*^G13C^ advanced cancer cell lines 24 hours after exposure to 100 nmol/L RMC-8839. Cells were plated in ULA conditions. Data are representative of three biological repeats per cell line. Vinculin was used as a protein loading control. **I,** Dose response analysis of advanced cancer cell lines harboring *KRAS*^G13C^ (H1734, H1355, HCC4087, and MOR), *KRAS*^G12C^ (H2030), *KRAS*^G12D^ (SKLU-1), and *KRAS*^G13D^ (H647) exposed to increasing concentrations of RMC-8839 for 168 hours. Viability was measured by CellTiter-Glo 3D. Data are expressed as a percentage of DMSO control. *n* = 3–4 biological repeats per cell line; mean ± SEM. IC_50_ values were calculated for each cell line and presented as a table. **J,** Apoptosis detection (left) and quantification (right) of H1734, MOR, HCC4087, and H1355 cells after 120 hours of exposure to 100 nmol/L RMC-8839. Cells were stained with annexin V and propidium iodide to detect apoptotic cells. *n* = 3 biological repeats per cell line; mean ± SEM. Statistical significance between comparisons was calculated using an unpaired Student *t* test (**B**, **E**, and **G**) and a two-way ANOVA (**J**). ****, *P* < 0.0001; ***, *P* < 0.001; **, *P* < 0.01; *, *P* < 0.05; ns > 0.05.

Using the previously described isogenic initiation NSCLC cell models (Fig. 2; Supplementary Fig. S4), treatment with RAS(ON) inhibitors as monotherapy was first examined at the point of mutant KRAS initiation. We noted that the RAS(ON) multiselective inhibitor, RMC-7977, inhibited RAS signaling in MLE-12 isogenic cell lines harboring each KRAS variant, as evidenced by reductions in ERK 1/2 phosphorylation (Fig. 4A and B). However, isogenic lines harboring *KRAS*^G13X^ were less sensitive to treatment compared with those with *KRAS*^G12X^, as evidenced by a higher IC_50_ (Fig. 4C), consistent with *KRAS* depletion in DepMap (Supplementary Fig. S7A) and other recent reports (13, 14). This was further supported using the isogenic BEAS-2B model, wherein cells expressing *KRAS*^G12X^ mutations exhibited greater sensitivity (lower IC_50_ and IC_75_) to RMC-7977 (Supplementary Fig. S7B).

We then exposed MLE-12 cells to 50 nmol/L RMC-8839, a RAS(ON) G13C-selective covalent inhibitor, observing complete cross-linking of FLAG-tagged KRAS^G13C^ and greater inhibition of RAS signaling (ERK 1/2 phosphorylation) in *KRAS*^G13C^ MLE-12 cells, relative to cells expressing other variants (Fig. 4D and E). At this concentration, we also observed partial cross-linking to FLAG-tagged KRAS^G12C^ and a modest reduction in ERK 1/2 phosphorylation in *KRAS*^G12C^ MLE-12 cells, but no effect on cells expressing other variants, confirming the selectivity of RMC-8839 for KRAS^G13C^ (Fig. 4D and E). Consistent with this, *KRAS*^G13C^ MLE-12 isogenic cells were most sensitive to the antiproliferative effects of RMC-8839, demonstrating the lowest IC_50_ (135 nmol/L), followed by *KRAS*^G12C^ MLE-12 (685 nmol/L). RMC-8839–treated MLE-12 cells harboring other KRAS variants exhibited IC_50_ values that were at least 10-fold higher than treated *KRAS*^G13C^ MLE-12 cells, further highlighting its selectivity for KRAS^G13C^ (Fig. 4F). Furthermore, *KRAS*^G13C^ BEAS-2B cells also showed the highest sensitivity to RMC-8839 (IC_25_ 7.5 nmol/L), followed by *KRAS*^G12C^ BEAS-2B cells (IC_25_: 42 nmol/L) and other variants (IC_25_: 85–3,245 nmol/L; Supplementary Fig. S7C).

Next, we exposed our advanced cancer cell line panel to the RAS(ON) multiselective inhibitor, RMC-7977, observing that *KRAS*^G13X^-mutant cell lines were more resistant to RAS inhibition than *KRAS*^G12X^ NSCLC cell lines, further highlighting that NSCLC cell lines harboring *KRAS*^G13X^ mutations are less dependent on KRAS-mutant signaling than those harboring *KRAS*^G12X^ mutations (Fig. 4G). With the RAS(ON) G13C-selective inhibitor, RMC-8839, in advanced cell lines, Western blotting revealed that it effectively binds to KRAS^G13C^, switching off MAPK signaling to various levels in *KRAS*^G13C^ NSCLC lines only (Fig. 4H; Supplementary Fig. S7D). We observed clear reductions in viability conferred by RMC-8839 in all four *KRAS*^G13C^ cell lines after 144 hours of exposure to the compound (Fig. 4I). However, the broad range in IC_50_ between the *KRAS*^G13C^ cell lines (20.3–1,202 nmol/L) revealed that responses were heterogeneous, with one *KRAS*^G13C^ cell line (H1355) exhibiting greater resistance to this compound. Treated cell lines harboring alternative *KRAS* mutations had significantly higher IC_50_ values ranging from 1,688 to 10,000 nmol/L (Fig. 4I; Supplementary Fig. S7E). This, again, clearly demonstrated the selectivity and activity of RMC-8839 when it is administered to advanced and more genetically complex *KRAS*^G13C^ models. Interestingly, H1734 and MOR cells, which demonstrated higher sensitivity to RMC-8839, show comparable sensitivity to RMC-7977, whereas the more resistant HCC4087 and H1355 cells showed greater sensitivity to RMC-7977, suggesting HCC4087 and H1355 cells have a greater reliance on WT RAS signaling as explored in the accompanying article by Seamon and colleagues (Supplementary Fig. S7F; ref. 49). Using a concentration of RMC-8839 that reduced viability in all four *KRAS*^G13C^ cell lines (100 nmol/L), we observed that RMC-8839 induced apoptotic cell death in the more sensitive cell lines only (H1734 and MOR cells), suggesting that this compound was conferring a cytostatic effect on the more resistant cell lines (HCC4087 and H1355; Fig. 4J).

Overall, these results with monotherapy demonstrated the mutant-selective targeting potential of RMC-8839 across isogenic and advanced *KRAS*^G13C^ cell line models but also underlined the importance of optimizing cell-intrinsic combination approaches in the pretreated *KRAS*-mutant NSCLC population. Given its novelty and potential to offer an attractive tolerability profile (in turn increasing the feasibility of finding a tolerable treatment combination), we next focused on identifying combination partners for RMC-8839.

### Selective Sensitivity of *KRAS*^G13C^ NSCLC to Chemotherapy

**Figure 5.**
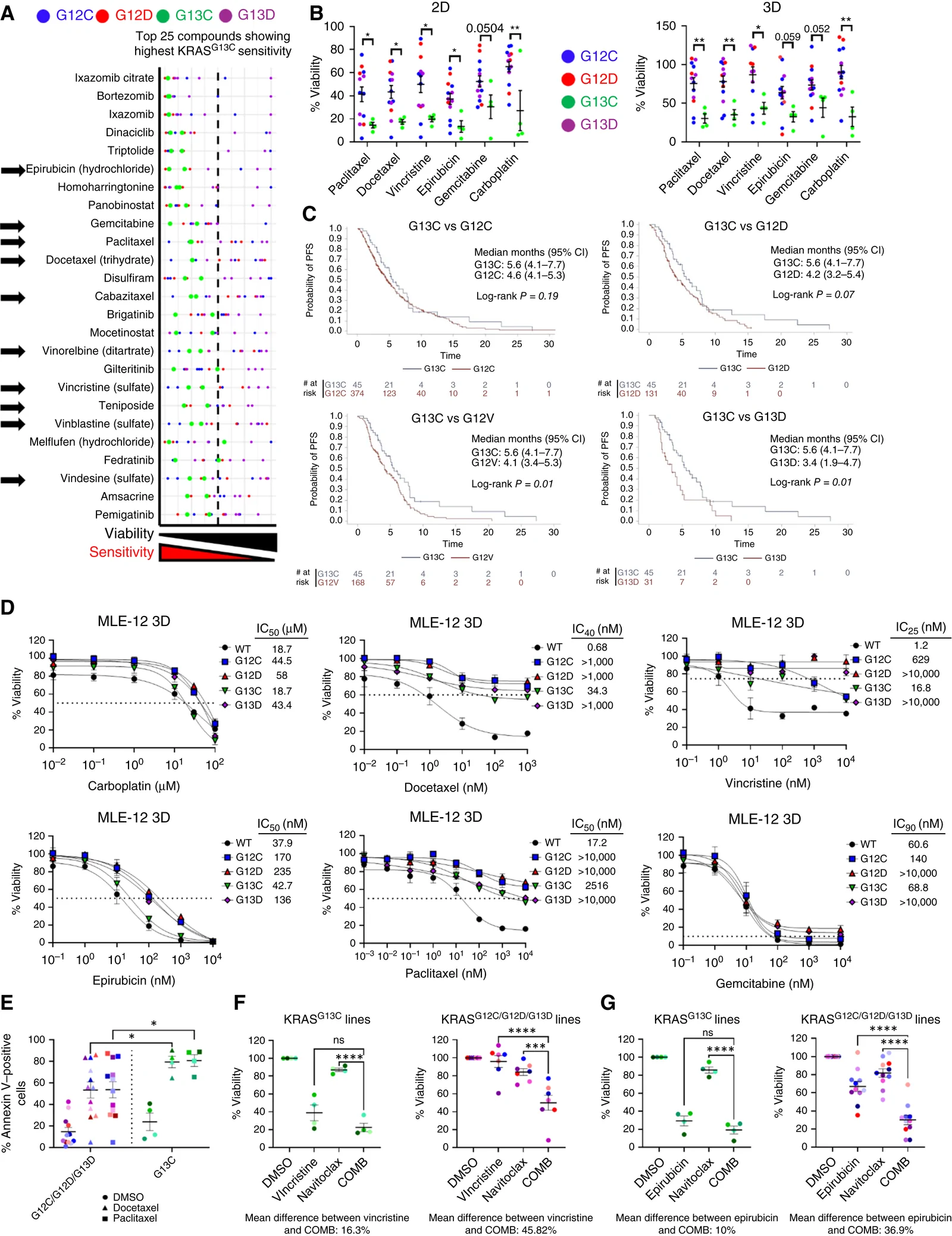
Selective sensitivity of *KRAS*^G13C^ NSCLC to chemotherapy. **A,** Top 25 compounds demonstrating the highest sensitivity to KRAS^G13C^-driven NSCLC cell lines compared with cell lines with other *KRAS*-mutant genotypes. Each dot represents a cell line and is the mean of two repeats. The dashed line indicates the midway point between the most resistant and the most sensitive cell line for every compound, with cell lines to the left of the dashed line deemed drug-sensitive and cell lines to the right deemed drug-resistant. Each cell line is considered a genotype-specific biological replicate: *KRAS*^G12C^ (blue), *n* = 4 cell lines; *KRAS*^G12D^ (red), *n* = 3 cell lines; *KRAS*^G13C^ (green), *n* = 2 cell lines; and *KRAS*^G13D^ (purple), *n* = 4 cell lines. The dot represents the mean of the technical duplicate. Black arrows indicate compounds of interest. **B,** 2D (left) and 3D (right) viability of advanced cancer cell lines expressing either *KRAS*^G12C^ (H358, H23, HCC1171, H2030, H1792, and HOP62), *KRAS*^G12D^ (SKLU-1, A427, and HCC461), *KRAS*^G13C^ (H1734, H1355, HCC4087, and MOR), or *KRAS*^G13D^ (H647, H1944, HCC515, and HCC1974) exposed to 1 μmol/L docetaxel, 1 μmol/L paclitaxel, 1 μmol/L vincristine, 1 μmol/L epirubicin, 1 μmol/L gemcitabine, and 100 μmol/L carboplatin. Viability was measured by CellTiter-Glo (3D) 72 hours later. Data are expressed as a percentage of DMSO control. *n* = 3 per cell line with the mean of three biological repeats shown as a dot. Error bars represent mean ± SEM of all cell lines within each condition (*KRAS*^G12C^ + *KRAS*^G12D^ + *KRAS*^G13D^ vs. *KRAS*^G13C^). Two *KRAS*^G12C^ lines (H2030 and HCC1171) were excluded from vincristine response in 3D analysis due to a viability increase of more than 180% relative to DMSO control. **C,** PFS between patients with *KRAS*^G13C^ and those with *KRAS*^G12C^, *KRAS*^G12D^, *KRAS*^G12V^_,_ or *KRAS*^G13D^ NSCLC receiving first-line chemotherapy. Data are taken from the Francophone de Cancérologie Thoracique dataset (51). **D,** Dose-response analysis of isogenic MLE-12 cells previously treated with DOX for 24 hours to induce KRAS variant expression in 0.5% serum, followed by a 72-hour treatment with a concentration range of indicated chemotherapeutics. Cells were plated in ULA conditions. Viability was analyzed by CellTiter-Glo 3D. Data are expressed as a percentage of DMSO control. *n* = 3 biological repeats per isogenic cell line; mean ± SEM. IC values were calculated for each cell line and presented as a table. **E,** Apoptosis quantification of all cell lines used in B (except H1792) after 72 hours of exposure to either 100 nmol/L docetaxel or 100 nmol/L paclitaxel. Cells were stained with FITC-conjugated annexin V to detect apoptotic cells. *n* = 3 biological repeats per cell line with the mean of three biological repeats shown as a dot. Error bars represent mean ± SEM of all cell lines within each condition. **F,** Viability of advanced cancer cell lines expressing (left) *KRAS*^G13C^ and (right) *KRAS*^G12C^, *KRAS*^G12D^, and *KRAS*^G13D^ grouped together, exposed to 1 μmol/L vincristine or either 100 or 1,000 nmol/L navitoclax or both compounds in combination. Viability was measured by CellTiter-Glo (3D) 72 hours later. Data are expressed as a percentage of DMSO control. *n* = 3 per cell line with the mean of 2 or 3 biological repeats shown as a dot. Error bars represent mean ± SEM of all cell lines within each condition. **G,** Viability of advanced cancer cell lines expressing (left) *KRAS*^G13C^ and (right) *KRAS*^G12C^, *KRAS*^G12D^, and *KRAS*^G13D^ grouped together exposed to 1 μmol/L epirubicin or either 100 or 1,000 nmol/L navitoclax or both compounds in combination. Viability was measured by CellTiter-Glo (3D) 72 hours later. Data are expressed as a percentage of DMSO control. *n* = 3 per cell line with the mean of 2 or 3 biological repeats shown as a dot. Error bars represent mean ± SEM of all cell lines within each condition. Statistical significance between comparisons was calculated using an unpaired Student *t* test (**B**), log-rank (Mantel–Cox; **C**), a two-way ANOVA (**E**), and a one-way ANOVA (**F** and **G**). COMB, combination. ****, *P* < 0.0001; ***, *P* < 0.001; **, *P* < 0.01; *, *P* < 0.05; ns > 0.05.

Due to the unique molecular profiles of each mutant KRAS isoform and the specific combination sensitivities previously reported with either *KRAS*^G12C^ or *KRAS*^G12D^ (34), we next aimed to identify if there were mutant-specific sensitivities associated with *KRAS*^G13C^-driven NSCLC that could inform combination partners for RMC-8839. We empirically approached this by treating our advanced *KRAS*-mutant NSCLC cell line panel, harboring *KRAS*^G12C^, *KRAS*^G12D^, *KRAS*^G13C^, or *KRAS*^G13D^ mutants, with a drug compound library that incorporated FDA-approved and clinically established chemotherapies or tyrosine kinase inhibitors (TKI; Supplementary File S1). The benefits of drug repurposing have been recently highlighted in the setting of *KRAS*^G12C^ NSCLC, noting that subsequent combinatorial clinical trials could be fast-tracked by coopting therapies that are already well characterized for toxicity and dose (50). In total, our compound library screen assessed 228 compounds in 13 NSCLC cell lines. We observed heterogeneous responses across all cell lines within each genotype (Supplementary Fig. S8A; Supplementary File S2). Filtering the top 25 compounds that showed enhanced sensitivity to two KRAS^G13C^-expressing cell lines used in the screen, several chemotherapies were noted as hits, including vinca alkaloids and taxanes, whereas cell lines harboring other *KRAS* genotypes showed more heterogeneous responses to these treatments (Fig. 5A). Other chemotherapies that the compound library screen indicated to be more effective in *KRAS*^G13C^-driven cell lines included gemcitabine and epirubicin. Assessing the relative benefits of different chemotherapies according to mutated *KRAS* codon (“*KRAS*^G12X^” vs. “*KRAS*^G13X^”), there was little difference in individual treatment vulnerabilities between these groups (Supplementary Fig. S8B). However, similar to their disparity of immunotherapy-associated biomarkers and comutations, important differences emerged when comparing *KRAS*^G13C^ and *KRAS*^G13D^ cells, with the relatively resistant nature of *KRAS*^G13D^ concealing *KRAS*^G13C^-specific vulnerabilities when grouped together as *KRAS*^G13X^ (Supplementary Fig. S8B).

We took forward five candidate chemotherapies for validation using a broader panel of cell lines (including additional *KRAS*^G13C^ cell lines MOR and HCC4087) in both 2D and 3D culture conditions, also including carboplatin, which was not covered in the screen. In this wider panel, higher sensitivity of *KRAS*^G13C^ cell lines to all six chemotherapeutic agents was evident in both 2D and 3D culture (Fig. 5B; Supplementary Fig. S8C). To corroborate our findings, we assessed a previously published study of patients with advanced NSCLC from the internationally reputed Intergroupe Francophone de Cancérologie Thoracique consortium, which reported survival outcomes of patients affected by *KRAS*-mutant NSCLC who received chemotherapy alone as first line (51). This study represents the largest cohort of NSCLC affected by *KRAS*^G13X^ mutations treated with chemotherapy (45 patients harboring a *KRAS*^G13C^ mutation, 31 patients with *KRAS*^G13D^), offering a unique opportunity to assess *KRAS*-mutant allele-specific differences in response to chemotherapy in the preimmunotherapy era. Comparing *KRAS*^G13C^ patients versus *KRAS*^non-G13C^-mutant patients, we observed a trend toward longer PFS in patients harboring *KRAS*^G13C^ (median months: 5.6 for *KRAS*^G13C^ patients vs. 4.4 for *KRAS*^non-G13C^-mutant patients; *P* = 0.09, log-rank; Supplementary Fig. S8D). PFS between *KRAS*^G13C^ patients and either *KRAS*^G12C^, *KRAS*^G12V^, *KRAS*^G12D^, or *KRAS*^G13D^ patients was then compared separately. Again, patients harboring *KRAS*^G13C^ overall had a longer PFS compared with those harboring other *KRAS* mutants, with statistical significance reached for comparisons with *KRAS*^G12V^ (median months: 5.6 for *KRAS*^G13C^ patients vs. 4.1 for *KRAS*^G12V^ patients; *P* = 0.01, log-rank) and *KRAS*^G13D^ patients (median months: 5.6 for *KRAS*^G13C^ patients vs. 3.4 for *KRAS*^G13D^ patients; *P* = 0.01, log-rank; Fig. 5C). Although these results were consistent with our *in vitro* results suggesting that *KRAS*^G13C^ is selectively chemosensitive, it should be noted that this was still a relatively small number of *KRAS*^G13C^ patients.

Aiming to characterize a mechanism for *KRAS*^G13C^ chemosensitivity, we first assessed whether it may be due to faster proliferation of our advanced *KRAS*^G13C^ NSCLC models, given the anticipated benefits of chemotherapies on cells/cancers that proliferate quickly (52). As expected from our prior experiments (Fig. 3A), there was a lack of correlation between the relative proliferation rate and chemotherapy-induced inhibition of viability (Supplementary Fig. S8E). We then reasoned that comutations in our advanced NSCLC panel may drive increased sensitivity of *KRAS*^G13C^ NSCLC to chemotherapy, given that LKB1 expression has been associated with paclitaxel resistance *in vitro* (53). Using the previously described DOX-inducible LKB1-overexpressing MOR cell line (Fig. 3E), we found that LKB1 expression only modestly increased resistance to chemotherapy (Supplementary Fig. S8F), suggesting that *STK11* mutations were not the driver of this phenotype. Although three out of four *KRAS*^G13C^ cell lines, including MOR, harbor mutations in *KEAP1*, these mutations have been shown to activate NRF2 signaling and protect cells from chemotherapy-induced oxidative stress (54, 55) and were therefore very unlikely to contribute to a chemosensitive phenotype. This suggests that the degree of sensitivity of *KRAS*^G13C^-driven models with a *KEAP1* mutation in response to chemotherapy may be even more striking in the context of WT *KEAP1* although further experiments involving the restoration of WT *KEAP1* would clarify this.

To further validate that chemosensitivity was increased in KRAS^G13C^-expressing cells, we exposed the isogenic MLE-12 models to increasing concentrations of all six chemotherapies and determined IC values. *KRAS*^WT^ MLE-12 cells consistently demonstrated the lowest IC values for five of six chemotherapies, whereas cells expressing *KRAS* mutations exhibited greater resistance. The level of resistance was variable and determined by the specific *KRAS* mutant, with *KRAS*^G12D^ MLE-12 cells exhibiting the strongest resistance to chemotherapy, whereas *KRAS*^G13C^ MLE-12 cells consistently demonstrated the least resistance (Fig. 5D). These findings suggested a direct link between chemoresistance and the *KRAS* mutational subtype and that the weaker oncogenic properties of *KRAS*^G13C^ are relevant to its chemosensitive phenotype.

*KRAS* mutations are known to increase therapy resistance through multiple mechanisms, such as increased apoptotic resistance (56), metabolic reprogramming and improved REDOX homeostasis (57), and enhanced drug efflux (58). If the reason underpinning the greater sensitivity of *KRAS*^G13C^-expressing cells to chemotherapy is the weaker oncogenicity of *KRAS*^G13C^, then we rationalized that these cells may be less able to activate these resistance mechanisms in response to treatment with chemotherapeutics. We focused on apoptosis and antiapoptotic resistance as possible mechanisms contributing to the vulnerability of *KRAS*^G13C^-driven NSCLC cell lines to chemotherapy. Apoptotic cell death in our NSCLC cell line panel was significantly higher in *KRAS*^G13C^ cell lines when treated with taxanes (Fig. 5E), suggesting that *KRAS*^G12C^, *KRAS*^G12D^, and *KRAS*^G13D^ mutations may promote stronger apoptotic resistance. Antiapoptotic proteins, such as BCL-2, BCL-XL, and MCL-1, play a critical role in promoting apoptotic resistance in cancer, actively suppressing both intrinsic and extrinsic cell death pathways (59, 60). Inhibiting BCL2, BCL-XL, and MCL1 using cell line–specific concentrations of navitoclax (100 or 1,000 nmol/L) in the presence of three chemotherapeutics, we observed that the addition of navitoclax only enhanced the overall activity of vincristine, epirubicin, and docetaxel by 16.3%, 10%, and 21.2%, respectively, in *KRAS*^G13C^ cell lines (Fig. 5F and G; Supplementary Fig. S8G). However, for *KRAS*^G12C^, *KRAS*^G12D^, and *KRAS*^G13D^ NSCLC cell lines, navitoclax overall exerted strong sensitization and enhanced the activity of the same chemotherapies by 45.82%, 36.9%, and 32.3%, respectively (Fig. 5F and G; Supplementary Fig. S8G). Overall, these findings suggest that the cytotoxic impact of chemotherapies in models driven by *KRAS*^G12C^, *KRAS*^G12D^, and *KRAS*^G13D^ is reduced due to stronger apoptotic resistance, a phenotype that can be overcome by suppression of antiapoptotic protein activity using navitoclax.

Collectively, these data reveal a unique vulnerability of *KRAS*^G13C^-driven NSCLC to chemotherapy. We highlight that despite harboring several comutations, which can increase *KRAS*^G13C^ oncogenicity, *KRAS*^G13C^ remains less capable than other, more biologically dominant KRAS mutants in promoting tumorigenic processes such as apoptotic resistance, increasing its sensitivity to common standard-of-care chemotherapies. Other *KRAS* genotypes benefit more from BCL-2 family inhibition, which may reflect genotype-specific baseline dependence on antiapoptotic proteins or differential drug efflux.

### RMC-8839 and Taxanes Synergize in *KRAS*^G13C^-Driven NSCLC Models

**Figure 6.**
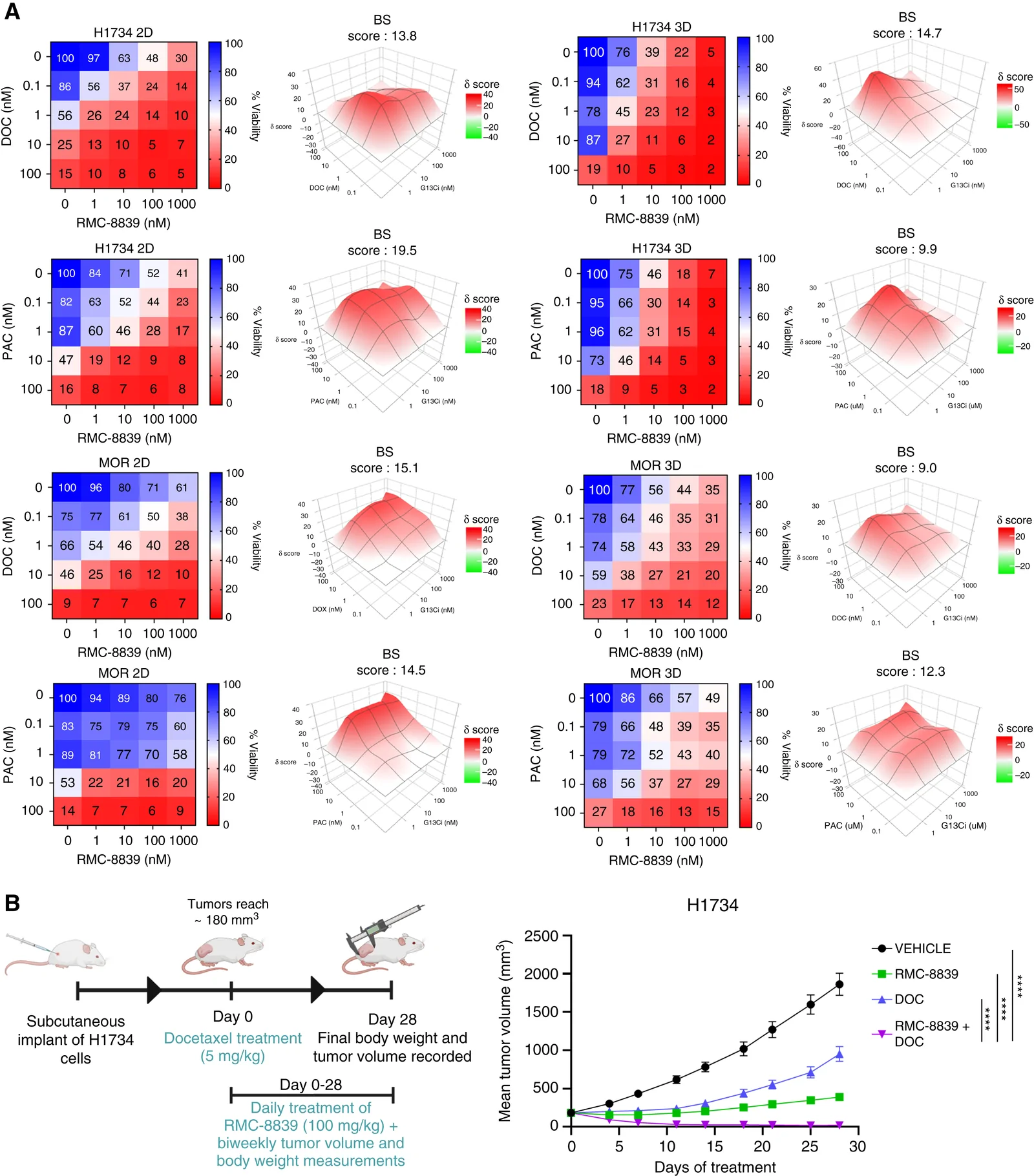
RMC-8839 and taxanes synergize in *KRAS*^G13C^-driven NSCLC models. **A,** H1734 and MOR cells were exposed to increasing concentrations of KRAS^G13C^ inhibition (RMC-8839) and either docetaxel (DOC) or paclitaxel (PAC) in both 2D and 3D conditions. After 72 hours, viability was analyzed by CellTiter-Glo (3D) and expressed as a percentage of DMSO control. Drug interaction was calculated using Bliss synergy (BS) analysis. *n* = 3 biological repeats per cell line; the mean is shown. BS less than −10 denotes antagonism; a BS score more than −10 but less than 10 denotes additivity; and a BS above 10 denotes synergy. In BS graphs, G13Ci indicates RMC-8839 **B,** (Left) Schematic: H1734 cells were transplanted subcutaneously into the flank of NOD-SCID mice. Treatments began when the mean tumor volumes reached approximately 183 mm^3^. A single intravenous dose of docetaxel at 5 mg/kg was administered on day 0; RMC-8839 was administered orally at 100 mg/kg daily for 28 days. Tumor volume and body weight were measured biweekly throughout the treatment period. (Right) Mean tumor volumes. *n* = 9 mice per group; mean ± SEM. Statistical significance between treatment groups was calculated using a two-way ANOVA. ****, *P* < 0.0001. **B,** (Left) Created in BioRender. Tinsley, K. (2026) https://BioRender.com/9dc6kig.

Choosing docetaxel as a clinically tractable chemotherapy that is regularly used in the second-line space for the treatment of NSCLC (in which novel trial combinations are typically introduced), we further investigated its promise as a combination partner for effective targeting of *KRAS*^G13C^ NSCLC. We first assessed the benefits of docetaxel with RMC-8839 in *KRAS*^G13C^ cell lines that were previously noted to be either sensitive or resistant to RMC-8839 monotherapy. As a mutant-selective inhibitor, RMC-8839 has the potential to offer a larger therapeutic window for effective combination relative to a RAS(ON) multiselective inhibitor, minimizing the risk of serious toxicity that remains a significant challenge for NSCLC translational progress (61, 62). Despite being a standard second-line treatment option for advanced NSCLC for 10 to 15 years, differences in docetaxel efficacy between *KRAS*-mutant subtypes have never been comprehensively reported (63). Highly additive or synergistic benefits (Bliss synergy score of >10) were observed using the combination of RMC-8839 and docetaxel or paclitaxel in sensitive H1734 and MOR cell lines, in both 2D and 3D culture (Fig. 6A). This combination was also associated with significantly increased apoptosis compared with the use of either drug alone (Supplementary Fig. S9A). In contrast, the combination of RMC-8839 with taxanes offered low-to-medium additivity in the more RMC-8839–resistant HCC4087 and H1355 cells (Supplementary Fig. S9B). We reasoned that taxanes did not cooperate with RMC-8839 in these cells due to the resistance of these cell lines to RMC-8839 monotherapy.

Finally, we tested the combination of RMC-8839 and docetaxel *in vivo* using a subcutaneous xenograft model in which tumors formed after the injection of H1734 cells (Fig. 6B). Both RMC-8839 and docetaxel individually led to tumor growth inhibition compared with the vehicle control. In contrast, the combination of RMC-8839 and docetaxel resulted in significant tumor regression, with all nine tumors exhibiting greater than 80% tumor volume reduction (Fig. 6B; Supplementary Fig. S9C). All treatments were tolerated based on body weight assessment (Supplementary Fig. S9D). We employed InvivoSyn (64), a tool to detect global drug synergy in *in vivo* studies across multiple time points [using both the highest single agent (HSA) and Bliss independence models of drug cooperation], identifying that the combination of RMC-8839 and docetaxel was extremely synergistic (Supplementary Fig. S9E), as evidenced by statistically significant combination index (CI) scores of 0.077 and 0.2333 (HSA and Bliss independence models, respectively; CI < 1 = synergy). Overall, these data demonstrate that the combination of KRAS^G13C^ inhibition and standard-of-care taxanes induces highly synergistic antitumor activity in RMC-8839–sensitive *KRAS*^G13C^ models, both *in vitro* and *in vivo*.

## Discussion

**Figure 7.**
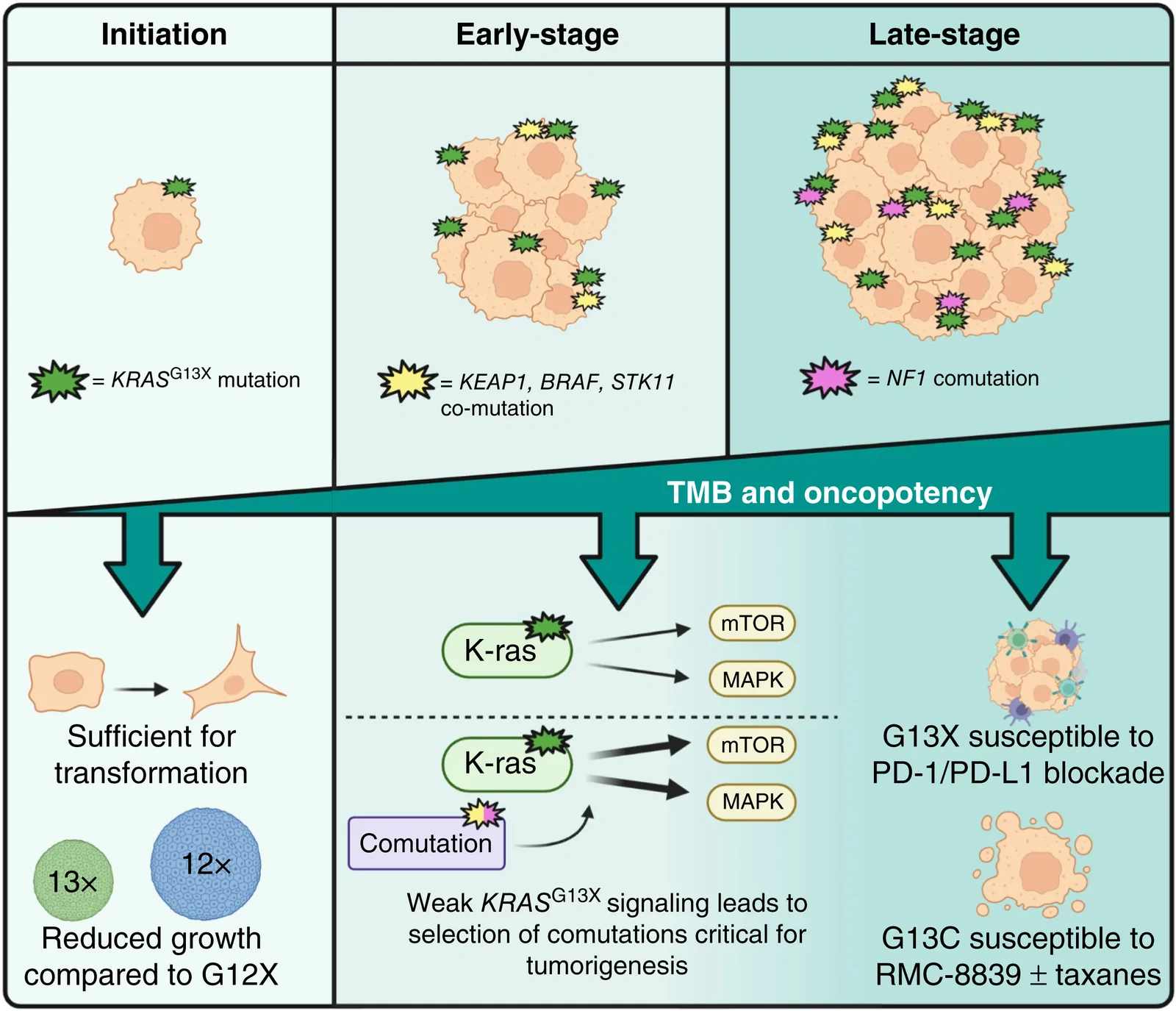
The phenotypes and comutations associated with early- and late-stage KRAS^G13X^-driven NSCLC and treatment vulnerabilities of KRAS^G13C^-driven NSCLC. *KRAS*^G13X^ mutations can transform epithelial cells but confer reduced growth and signaling properties compared with *KRAS*^G12X^ mutations. In early-stage *KRAS*^G13X^-driven tumors, mutations in *KEAP1*, *STK11*, and *BRAF* occur, whereas in late-stage tumors, mutations in *NF1* occur. These upregulate MAPK and mTOR signaling to promote tumor progression and malignancy. Advanced *KRAS*^G13X^ tumors demonstrate both partial sensitivity to PD-1/PD-L1 blockade and increased sensitivity to chemotherapeutics ± RMC-8839. Created in BioRender. Tinsley, K. (2026) https://BioRender.com/k9dtdzq.

In this study, we have assimilated functional, bioinformatic, and clinical research to comprehensively characterize the phenotype and treatment vulnerabilities of *KRAS*^G13X^ mutations in NSCLC (Fig. 7). With examination of cellular-based models that represent different stages of RAS addiction and NSCLC tumor evolution, we show that *KRAS*^G13X^ mutations confer a weakly oncogenic phenotype (particularly *KRAS*^G13C^) in the early stages of tumorigenesis. Using an advanced *KRAS*-mutant NSCLC cell line panel and an internationally recruited cohort of patients with stage IV *KRAS*-mutant NSCLC (RAS-PM), we show that at the point of advanced cancer, the oncopotency of *KRAS*^G13X^ NSCLC seems equivalent to NSCLC harboring other *KRAS* mutations. Hypothesizing that *KRAS*^G13X^ mutations coopt critical pathogenic comutations to drive their oncogenic phenotype in advanced NSCLC, we complemented functional characterization of NSCLC with parallel interrogation of genomic databases that represent a combination of early- and late-stage *KRAS*^G13X^ NSCLC, showing that *KRAS*^G13X^ NSCLCs are more likely than *KRAS*^G12X^ NSCLCs to harbor pathogenic comutations that influence cell-autonomous effects and immunotherapy responses. We finished by identifying chemotherapy as a *KRAS*^G13C^-specific vulnerability, which we show may be linked to the inability of *KRAS*^G13C^ to promote tumorigenic processes such as apoptotic resistance. Furthermore, we confirmed the potential of combination therapy with RMC-8839, a novel RAS(ON) G13C-selective inhibitor, and chemotherapeutics. In summary, we describe a novel directly targetable subset of NSCLC, adding KRAS^G13C^ to the ever-expanding list of tractable molecular targets in NSCLC, in which previous successes have included TKI treatment of *EGFR* and *BRAF-*mutant cancers, as well as tumors with *ALK*, *ROS1*, and *RET* fusions (65–67). Our data suggest that in line with recent successful clinical trial data on chemotherapy combination with EGFR inhibitors, combination with chemotherapy should be considered early with KRAS inhibition while also suggesting that chemotherapy monotherapy may confer significant benefits to existing patients affected by *KRAS*^G13C^ NSCLC.

We recently demonstrated that *KRAS*^G12D^ is a stronger oncogene compared with *KRAS*^G12C^ in NSCLC initiation, a phenotype linked to higher PI3K–AKT–mTOR signaling (34). Using this example, we now show that mutations at codon 13 confer weaker oncogenicity compared with codon 12 mutants in models reflective of early-stage disease, a finding that is associated with reduced activation of MAPK and PI3K–AKT–mTOR signaling. In our advanced NSCLC models, we no longer observed allele-specific differences in potency between *KRAS*^G12X^ and *KRAS*^G13X^ cell lines, which seem to have comparable KRAS signaling. The reduced capability of KRAS^G13X^ (and KRAS^G12C^) to activate MAPK and PI3K–AKT–mTOR signaling compared with KRAS^G12D^ in our initiation models may explain the higher propensity of *TP53*, *NF1*, *BRAF*, and *KEAP1* to comutate within established tumors driven by weaker oncogenes such as KRAS^G13X^ (68). Given that mTOR signaling promotes NSCLC progression (44) and KEAP1 negatively regulates mTOR signaling (41, 42), loss-of-function *KEAP1* alterations are acquired through selective pressures to drive tumorigenesis by enhancing mTOR signaling. In addition, *NF1* loss-of-function alterations increase RAS activation (GTP-loading), thereby upregulating RAS activity and increasing tumorigenic signaling (9). As *KRAS*^G12D^ is a stronger oncogene that can activate the MAPK and mTOR pathways effectively, additional mutations converging on the RAS pathway are therefore less frequent in *KRAS*^G12D^-driven NSCLC.

Translational advances in RAS precision medicine have progressed exponentially since the first preclinical inhibitor of KRAS^G12C^, which binds to the switch-II pocket of KRAS^G12C^ and locks it in its OFF state (69). This breakthrough acted as a watershed for the discovery of KRAS inhibitors targeting the switch-II pocket, such as the KRAS^G12D^-selective MRTX1133 (70, 71) and the pan-KRAS inhibitors BI-2865/BI-2493 (72). Inhibitors targeting RAS by alternative mechanisms, such as tricomplex RAS(ON) multiselective inhibitors, have also been developed, demonstrating superior potency over first-generation switch-II pocket binding inhibitors (13, 14). Two KRAS^G12C^ inhibitors, sotorasib and adagrasib, have so far been approved for use in the clinic (6, 73). However, a myriad of mechanisms of KRAS^G12C^ inhibitor resistance have been described in both preclinical and clinical studies (15–17). To expedite translation and maximize the chances of clinical success, refining the selection of patients with NSCLC and treatment combination partners using rational preclinical choices will be important. In particular, our preclinical data demonstrate that NSCLC harboring *KRAS*^G13C^ is vulnerable to a novel RAS(ON) G13C-selective inhibitor, RMC-8839, in which antitumor activity is potentiated by its combined use with chemotherapy (for tumor cells responsive to RMC-8839 monotherapy). Platinum-based chemotherapy, vinca alkaloids, and taxanes are frequently used in the lung cancer clinic, but their use has not previously been considered with this level of patient selection. Furthermore, RAS(ON) targeting with a mutant-selective inhibitor is anticipated to offer an improved tolerability profile relative to a RAS(ON) multiselective inhibitor, with preliminary data from single-arm phase I to II studies of the RAS(ON) G12D-selective inhibitor zoldonrasib supportive of this (74, 75). Going forward, it will be important to understand the clinical tolerability profile of the RAS(ON) G13C mutant-selective inhibitor, RMC-8839, and whether it can offer a larger therapeutic window for chemotherapy combinations. Part of this challenge will involve advancing our understanding of why *KRAS*^G13C^ is particularly vulnerable to chemotherapy, with antiapoptotic resistance expected to be one of several contributing mechanisms.

In tandem with Seamon and colleagues (49), we describe the RAS(ON) G13C-selective tricomplex inhibitor, RMC-8839, for the first time. In the complementary work, isogenic cells with *KRAS* codon 13 mutations show reduced tumorigenic potential when compared with *KRAS* codon 12 mutants. This report also notes that co-occurring RAS pathway mutations, including *NF1* loss, are significantly more common in patients with *KRAS* codon 13 mutants than in *KRAS* codon 12 mutant tumors. Additionally, upon near-complete target engagement with RMC-8839, one third of *KRAS*^G13C^-mutant human cancer cell lines showed incomplete suppression of RAS pathway signaling, suggesting a complementary role for WT RAS(ON). To pharmacologically inhibit WT RAS(ON), RMC-8839 was combined with a RAS(ON) multiselective inhibitor, demonstrating deeper suppression of the MAPK pathway and greater reductions in *KRAS*^G13C^-driven cancer cell line viability *in vitro* and *in vivo*. Taken collectively, the preclinical and clinical data across our reports suggest that KRAS^G13C^-driven cancer is a directly targetable molecular subset of NSCLC.

Despite significant regional variation in *KRAS*^G13X^ prevalence in NSCLC, our survey of early and advanced stage NSCLC cohorts suggests that, in the context of absolute patient numbers, the introduction of a RAS (ON) G13C-selective inhibitor, for example, RMC-8839, to the clinic could offer significant impact: 3.1% and 5.6% of early- and advanced-stage *KRAS*-mutant lung adenocarcinomas were affected by *KRAS*^G13C^. Translated into the number of new lung cancer cases affecting the US per annum (∼228,000 lives), this is therefore a treatment that offers the potential to improve the care of 6,840 to 11,400 patients with lung cancer on an annual basis. Given that other targeted therapies for rare (<2% of total cases) molecular subtypes of NSCLC have demonstrated improved outcomes and achieved regulatory approval in multiple geographies, the clinical challenge of introducing trials addressing KRAS^G13C^ can also be considered achievable.

Although the expanding list of successful molecular targets in NSCLC generally represents a paradigm-shifting improvement in its care, progress with molecular therapies in the majority of patients who are affected by smoking-related disease remains slower-paced than previous breakthroughs that have been observed with TKIs in never- and light-smoking patients. Allele-specific and GTP-bound targeting of KRAS, one of the most frequently mutated oncogenes in cancer, has now revealed the potential benefits of investigational small molecules that can confer clinical activity and offer a large therapeutic window for patients who are often affected by significant frailty and comorbidities (76). Following on from the clinical descriptions of KRAS^G12C^ inhibitors and the RAS(ON) multiselective inhibitor, daraxonrasib, direct targeting of KRAS^G13C^(ON) with RMC-8839, chemotherapy, or their combination offers a potential next step toward effective translation for patients affected by unmet clinical need.

## Methods

### RAS-PM Patient Analysis

An international real-world dataset from 804 *RAS*-mutant cancers, with advanced stage (IIIb/IV) NSCLC from four tertiary cancer centers: (i) Christie NHS Foundation Trust (Manchester, UK), (ii) Gustave Roussy Cancer Centre (Paris, France), (iii) Stanford Cancer Center (California, USA), and (iv) Catalan Institute of Oncology, L’Hospitalet. RAS-PM was registered and approved as a quality improvement project (reference: SE18/2251) with additional registration at the multiple participating sites, with the coordinating center being the Christie NHS Foundation Trust, Manchester, UK. Retrospective data were extracted and shared only in deidentified form under local information governance agreements and were fully anonymized before analysis and reported in aggregate form to ensure confidentiality. Given the minimal risk, retrospective, and quality improvement nature of the work, formal research ethics committee review and individual informed consent were not required. Key inclusion criteria were as follows: (i) stage IIIb or IV NSCLC, (ii) confirmed *RAS* mutation with DNA coding and protein sequencing as defined by HGVS nomenclature, (iii) key baseline clinical and genomic details available, (iv) treatment and survival outcomes available, and (v) received at least one cycle of systemic anticancer therapy. Key exclusion criteria were stated as follows: (i) inconclusive or unconfirmed histologic NSCLC diagnosis, (ii) *WT RAS* cancers, and (iii) inadequate details on baseline characteristics or survival. This dataset was used to explore PFS and OS outcomes in patients who had received immunotherapy, as well as in which TMB, PD-L1 status, and comutational prevalence were known. PFS was defined as the length of time from the commencement of baseline therapy until progression or death, whichever occurred first. OS was defined as the length of time from the commencement of baseline therapy until death. A data cutoff of September 30, 2025, was used: If no progression or death had occurred, censoring was applied using the date of the last follow-up. Note: For comparative analysis of ICI outcomes in *KRAS*^G13X^ patients harboring either a *TP53* mutation alone versus *STK11* and/or *KEAP1* mutations, three patients had co-occurring *STK11/TP53* or *KEAP1/TP53* mutations and demonstrated characteristics of an immune-cold TME (such as low PD-L1) and were included in the *STK11* and/or *KEAP1* subgroup.

### cBioPortal Patient TMB Analysis

TMB (mut/Mb) was analyzed across 14 studies of patients with NSCLC deposited on cBioPortal. Patients with multiple *KRAS* mutations were excluded from this analysis.

### Genomics England 100KGP Cohort Analysis

The National Genomic Research Library, which holds data provided by patients and collected by the UK National Health Service as part of their care, is managed by Genomics England Limited (the National Genomics Library version 5.1, https://www.genomicsengland.co.uk/). WGS was performed on paired tumor–normal pairs from patients recruited to the 100k Genomes Project, release version 16, with sequence alignment to the human reference genome GRCh38 (Genome Research Consortium human build 38). Variant Effect Predictor version 109.0 was used to identify *KRAS* variants *KRAS*^G12C^, *KRAS*^G12D^, *KRAS*^G13C^, and *KRAS*^G13D^ and co-occurring mutations of interest (*TP53*, *STK11*, *NF1*, *ATM*, *KEAP1*, and *BRAF*). A full description of the methodology is published in the 100KGP cancer-specific clinical data—Genomics England Research Environment User Guide. Statistical analysis was performed in R version 4.2.1.

### Analysis of PFS Outcomes in Patients with *KRAS*-Mutant NSCLC Receiving First-line Chemotherapy

This involved a retrospective analysis of a previously published cohort of patients. For the current study, comparisons were made between patients with *KRAS*^G13C^ NSCLC and either all other patients with *KRAS*-mutant NSCLC or patients with NSCLC with specific mutations only (51). Patient recruitment, eligibility, ethics, and methods of statistical analysis can be found in the original publication.

### Analysis of Immune Signatures Using TCGA Firehose Legacy Dataset

TCGA lung adenocarcinoma mutation data were obtained from the publicly available TCGA Firehose Legacy repository (data_mutations.txt; ref. 33). Samples were subset to primary tumors, and mutation calls were parsed to extract codon-level alterations for *KRAS* using the annotated HGVSp_Short and Codons fields. Immune signatures were computed by taking the variance-stabilizing transformed mean expression of the constituent signature genes for each sample using the original gene lists from Danaher and colleagues (77) and Davoli and colleagues (78). Analysis was performed in RStudio (version 2025.09.2+418) running R 4.5.2 (October 31, 2025).

### Cell Culture

All cell lines were cultured at 37°C with 5% CO_2_. H358 (RRID:CVCL_1559), HCC1171 (RRID:CVCL_5126), H1792 (RRID:CVCL_1495), H2030 (RRID:CVCL_1517), H23 (RRID:CVCL_1547), HOP-62 (RRID:CVCL_1285), A427 (RRID:CVCL_1055), SKLU-1 (RRID:CVCL_0629), HCC461 (RRID:CVCL_5135), H1734 (RRID:CVCL_1491), H1355 (RRID:CVCL_1464), HCC4087 (RRID:CVCL_9Z18), MOR (RRID:CVCL_2618), H647 (RRID:CVCL_1574), HCC515 (RRID:CVCL_5136), H1944 (RRID:CVCL_1508), and HCC1974 (RRID:CVCL_V615) cells were cultured in RPMI 1640 medium (Gibco, #21875034) supplemented with 1% penicillin/streptomycin (P/S; Gibco, #15140122) and 10% tetracycline-free FBS (TF-FBS; Biosera, #FB-1001T). BEAS-2B (RRID:CVCL_0168), *KRAS*-mutant MEFs, and Lenti-X 293T (RRID:CVCL_4401) cells were cultured in DMEM (Gibco, #41966-029) with 1% P/S and 10% TF-FBS. MLE-12 cells (RRID:CVCL_3751) were cultured in DMEM/F-12 (Gibco, #11330-032) supplemented with 2% TF-FBS, 1% P/S, 2 mmol/L glutamine, 30 nmol/L sodium selenite, 5 μg/mL insulin, 10 nmol/L hydrocortisone, 10 μg/mL transferrin, and 10 nmol/L β-estradiol. During experimentation, BEAS-2B and MLE-12 cells were cultured in the same media except with 2.5% (BEAS-2B), 0.5%, or 0.1% (MLE-12) TF-FBS. BEAS-2B, H358, H23, A427, SKLU-1, HCC1171, H1792, H2030, and HOP62 cells were purchased from ATCC. Lenti-X 293T cells were purchased from Takara Bio. HCC461, H1734, H1355, HCC4087, H1944, HCC515, H647, and HCC1974 cells were kindly donated by John Minna (UT Southwestern, USA). MOR cells (ECACC 84112312) were purchased from Culture Collections. *KRAS*-mutant MEFs were provided by the Frederick National Lab for Cancer Research (NCI Frederick). Cell line authentication (Verogen MainstAY kit) and mycoplasma testing (MycoAlert Mycoplasma Detection kit, Lonza, #LT07-318) were carried out regularly. Cell culture images were taken using the Evos XL core imaging system (RRID:SCR_022190). Cells were cultured for up to 15 passages after thawing, during which time experiments were conducted.

### Generation of Isogenic *KRAS*-Mutant MLE-12 and BEAS-2B Cell Panels

Isogenic MLE-12 and BEAS-2B cell line panels expressing different *KRAS* mutations were carried out as previously described (34) using AttL5-AttR1 N-terminal FLAG tag; AttL1-AttL2 human KRAS4b constructs encoding FLAG-tagged *KRAS*^WT^, *KRAS*^G12C^, *KRAS*^G12D^, *KRAS*^G13C^, or *KRAS*^G13D^; and a TRE3G promoter fragment.

### Generation of LKB1-Overexpressing MOR Cells

N-terminal HA-tagged *STK11* was cloned into the DOX-inducible pCW57 plasmid using restriction enzymes SalI and MluI. Following bacterial transformation in One Shot Stbl3 competent cells and colony selection, successful recombination was confirmed by Sanger sequencing. Lentiviral transduction was carried out using Lenti-X 293T cells, third-generation lentiviral packaging plasmids, and polybrene. Transduced MOR cells were selected with 5 μg/mL puromycin.

### siRNA Transfection

Reverse transfection of siRNA oligonucleotides was performed using Lipofectamine RNAiMAX Reagent (Thermo Fisher Scientific, reference 13778150) according to the manufacturer’s instructions. *NF1*-targeting and nontargeting control siRNA pools were used (ON-TARGETplus human *NF1* siRNA SMARTpool, Dharmacon, reference L-003916-00-0005, target sequences: GAACAAACCAUAGCUAUAA, CAGAUAAUCCGUAUUCUUA, CGAAGUCGCUGCAGCCUAA, GCAAGUACUUACAUCAAUU; ON-TARGETplus nontargeting pool, Dharmacon, reference D-001810-10-05, target sequences: UGGUUUACAUGUCGACUAA, UGGUUUACAUGUUGUGUGA, UGGUUUACAUGUUUUCUGA, UGGUUUACAUGUUUUCCUA). siRNA (25 and 50 nmol/L) was used for HCC515 and H647 cells, respectively.

### Reagents

RMC-8839 [RAS(ON) G13C-selective inhibitor] and RMC-7977 [RAS(ON) multiselective inhibitor] were supplied by Revolution Medicines. DOX (#D9891) was purchased from Sigma. Ninety-six-well ULA plates (#7007), twenty-four-well ULA plates (#3473), and six-well ULA plates (#3471) were purchased from Corning. Gemcitabine (#CAY9003096), vincristine (#CAY11764), and paclitaxel (#CAY10461) were purchased from Cayman Chemicals. Docetaxel (#D5709) and epirubicin hydrochloride (#E6235-1) were purchased from LKT Labs. Docetaxel (#HY-B0011) for *in vivo* experimentation and navitoclax (#HY-10087) were purchased from MedChemExpress. Carboplatin (#S1215) was purchased from SelleckChem. All compounds were dissolved in DMSO (#D8418, Sigma), except for carboplatin, which was dissolved in phosphate-buffered saline (PBS).

### Proliferation and Viability Assays

For isogenic *KRAS*-mutant MLE-12 cells, proliferation and viability in response to DOX and/or drug treatment were measured by growing spheroids using 96-well ULA plates and using CellTiter-Glo 3D (Promega, #G9681) according to the manufacturer’s guidelines. For isogenic *KRAS*-mutant BEAS-2B cells, proliferation and viability in response to DOX and/or drug treatment were measured by growing spheroids using 96-well ULA plates and by using two readouts: (i) measuring spheroid area using ImageJ software (RRID:SCR_002285) and (ii) using CellTiter-Glo 3D according to the manufacturer’s guidelines. For human NSCLC cell lines, proliferation was measured by bright-field evaluation of cell confluence using the Incucyte SX3 (Essen Instruments), and viability in response to drug treatment was assessed under adherent conditions using CellTiter-Glo 2D (Promega, #G7570) and under ULA conditions in 96-well ULA plates using CellTiter-Glo 3D. DOX and drug treatments were carried out 24 hours after seeding. For *KRAS*-mutant MEFs, proliferation was assessed by growing cells as spheroids in 96-well ULA plates and measuring spheroid area using ImageJ software.

### Western Blotting

Cells were seeded onto six-well ULA plates and, after the indicated time point with or without exposure to the indicated compounds, were lysed using RIPA buffer (Sigma, #R0278) containing phosphatase inhibitor cocktail PhosSTOP (Roche, #4906845001) and protease inhibitor cocktail (Sigma, #P8340). Protein lysates were generated, resolved by gel electrophoresis using 4% to 12% Bis-Tris NuPAGE gels in MES running buffer, and transferred to polyvinylidene difluoride membranes (Merck, #IPVH00010). After transfer, membranes were blocked in PBS-Tween containing 5% BSA and then incubated with primary antibodies overnight. This was followed by incubation in horseradish peroxidase–conjugated secondary antibodies, exposure to enhanced chemiluminescence substrate (Pierce, #32106), and imaging using a Bio-Rad Chemidoc Touch Imaging System (RRID:SCR_021693). Primary and secondary antibodies (and relevant RRIDs) used for Western blotting are outlined in Supplementary Table S4. For all Western blotting experiments, phosphorylated proteins and total proteins were probed on separate blots. A representative loading control from either blot is presented in cropped panels to represent both blots from the same experiment. Where necessary, if the loading control is the same molecular weight as a different protein of interest, the blot was stripped and reprobed for the loading control protein (β-actin in all cases). Western blots were quantified using ImageJ.

### Apoptosis Detection

For the detection of apoptotic cell death, cells were seeded in six-well plates. The next day, cells were treated with the indicated compounds. After 72 or 120 hours, cells were harvested and stained with FITC-conjugated annexin V (BioLegend, #640945) alone or in combination with propidium iodide (Sigma, #P4864) where indicated. Cells were analyzed on a BD Fortessa flow cytometer or Attune NxT flow cytometer (Invitrogen, RRID:SCR_019590), and apoptosis was calculated by gating on annexin V and PI-positive cells, recording gated 10,000 events per condition and expressing positive cells as a percentage of the total cell population. Data were analyzed using FlowJo10 (RRID: SCR_008520). For detection of caspase-3/7 activation, cells were seeded in 96-well ULA plates and treated with the indicated compounds the next day. Twenty-four hours later, caspase-3/7 activation was quantified using Caspase-Glo 3/7 (Promega, #G8090).

### CD44 Surface Staining

Isogenic *KRAS*-mutant BEAS-2B and MLE-12 cells were seeded in six-well ULA plates and exposed to 100 ng/mL DOX. Twenty-four hours later, cells were harvested and stained with APC-conjugated CD44 antibody and APC-conjugated isotype control antibody (Supplementary Table S4) in staining buffer (PBS/5% FBS). Median fluorescence intensity of the fluorophore per condition was measured using a BD Fortessa flow cytometer recording 10,000 gated events per condition. Data were analyzed using FlowJo10.

### Compound Library Screening

A total of 500 to 1,000 cells were seeded onto a 384-well plate using a Formulatrix MANTIS (RRID:SCR_019923). The next day, each plate was exposed to 228 preselected FDA-approved anticancer drugs using an Integrated Labcyte Echo Liquid Handling Robot at a concentration of 1 to 10 μmol/L. Seventy-two hours later, viability was measured by CellTiter-Glo 3D using the Integrated Labcyte Echo Liquid Handling Robot. RStudio was used to define relative sensitivity: The midway point between the most resistant and the most sensitive cell line for every compound was calculated. The cell lines used in this screen were H1792, H2030, H23, HCC1171, SKLU-1, HCC461, A427, H1355, H1734, H1944, H647, HCC515, and HCC1974. The compounds are listed in Supplementary File S1.

### Gene Essentiality Analysis

Cell line data were downloaded from the Broad Institute’s DepMap portal (RRID:SCR_017655; https://depmap.org/portal/download/; ref. 79). *KRAS* mutation status was assigned to cell lines from the 20Q2 release of Cancer Cell Line Encyclopedia mutation data. Achilles CRISPR/Cas9 (Chronos)–derived *KRAS* “dependency” scores per *KRAS* genotype were derived from the portal. A lower dependency score indicates a higher probability that the gene is essential. A score of 0 is considered nonessential, and a score of −1 or less is considered essential. DepMap and OncoKB were also used to detail the comutations within advanced NSCLC cell lines.

### *In Vivo* Therapeutic Study

All procedures pertaining to animal handling, care, and treatment were carried out in compliance with all applicable regulations and guidelines approved by the Institutional Animal Care and Use Committee (IACUC). Female NOD SCID mice 6 to 8 weeks old (AniKeeper Biotech Co.) were housed in polycarbonate cages kept within a well-ventilated room, which was maintained at 20°C to 26°C and at a relative humidity of 40% to 80%. Animals were injected subcutaneously under the right flank with H1734 cells at a density of 1 × 10^7^ cells [mixed 1:1 with Matrigel (Corning, #354235)] per mouse. Mice were randomized, and treatments began when the mean tumor volume measured approximately 183 mm^3^. RMC-8839 was formulated in 10% DMSO (Solarbio, #D8371), 20% PEG400 (Sigma, #P3265), 10% Solutol HS15 (Sigma, #42966), and 60% water and was administered daily at 100 mg/kg by oral gavage for 29 days. Docetaxel was formulated in 5% ethanol, 5% Cremophor EL (Sigma, #C5135), and 90% saline and was administered once at the start of the treatment (day 0) at 5 mg/kg by i.v. injection. Tumors were measured biweekly using a digital caliper, by an unblinded employee who was not involved in the design or analysis of study results. The tumor volume (mm^3^) was calculated using the following formula: Volume = [(width)^2^ × length]/2, where width and length are measured in millimeters (mm). Mice were also weighed biweekly. No mice were excluded from the study.

### Chicken Egg Xenograft Analysis of Tumor Growth

*In vivo* experiments do not require any special permits as long as the embryos are sacrificed before hatching, as in this study. We used premium specific pathogen-free, fertile, 11-day-incubated embryonated chicken eggs. We inoculated 1.5 × 10^6^ BEAS-2B cells (expressing the indicated *KRAS*-mutant variants), previously treated for 24 hours with DOX (100 ng/mL) or vehicle (medium + deionized water), in 2.5% FBS medium mixed 1:1 with Matrigel, into the CAM. Three days after inoculation, the tumors were treated again with DOX or vehicle. After 7 days, the tumors were excised and weighed. Tumor weight was normalized by dividing the weight of each tumor by the mean of the tumor weights in the −DOX (vehicle) condition. The Robust regression and Outlier removal (ROUT) method (Q = 1%) was applied to the normalized tumor weight to exclude outliers.

### Drug Interaction Analyses

For *in vitro* work, dose–response matrices using two compounds were carried out using CellTiter-Glo 3D, and the data were uploaded onto SynergyFinder (RRID:SCR_019318) to determine synergy using the Bliss independence model (80). A synergy score of < −10 denotes drug antagonism; between −10 and 10 denotes drug additivity, and >10 denotes synergism. For *in vivo* work, the InvivoSyn R package (64) was used, which assessed synergy under two models (HSA model and Bliss independence model) and calculated a CI. CI > 1 denotes drug antagonism, CI = 0 denotes drug additivity, and CI < 1 denotes drug synergy.

### Statistical Analysis

Data were graphed using GraphPad Prism10 (RRID:SCR_002798), Biorender (RRID:SCR_018361), and IBM SPSS Statistics version 931.0.0.0 (RRID:SCR_016479). Data are presented as the mean of independent biological replicates ± standard error of the mean (SEM), unless stated otherwise. Statistical tests and *P* values were generated using GraphPad Prism 10 and IBM SPSS Statistics version 31.0.0.0 and are specified within the figure legends. To determine statistical significance between two conditions, a two-tailed Student *t* test was used. To determine statistical significance between multiple comparisons, a one- or two-way ANOVA was used. The log-rank (Mantel–Cox) test was used for survival curve analyses. To determine associations between comutations, contingency analysis and Fisher exact testing were used. ns, not significant, *, *P* < 0.05; **, *P* < 0.01; ***, *P* < 0.001; ****, *P* < 0.0001.

### Ethical Approval

For *in vivo* work, studies were carried out in compliance with all applicable regulations and guidelines of the relevant IACUC. For RAS-PM patient studies, data collection protocols were approved by the local governance committee. No ethical approval is needed for chicken egg xenograft experiments as long as embryos are sacrificed before hatching.

## Supplementary Material

Supplementary Tables S1-S4 Supplementary Table 1 - Baseline characteristics of RAS-PM patients. Supplementary Table 2 - Treatment information for RAS-PM patients. Supplementary Table 3 - Number of patients tested for each co-mutation within RAS-PM, independently of treatment regimen. Supplementary Table 4 – Antibodies.

Supplementary Figures S1-S9 Supplementary Figure S1: Analysis of prognostic variables and genomic features in *KRAS*-mutant NSCLC patient datasets. Supplementary Figure S2: The impact of co-mutations on immunotherapy outcomes in KRAS^G13X^ NSCLC patients. Supplementary Figure S3: *KRAS*-mutant isoform-specific immune signatures from TCGA Firehose Legacy dataset. Supplementary Figure S4: Analysis of KRAS^G13X^ oncogenicity using isogenic models. Supplementary Figure S5: *KRAS*-mutant NSCLC cell line panel co-mutations and growth rate analyses. Supplementary Figure S6: *KRAS*-mutant NSCLC cell line panel signaling. Supplementary Figure S7: Analysis of (K)RAS (ON) inhibition in NSCLC models. Supplementary Figure S8: KRAS^G13C^-driven NSCLC is selectively vulnerable to chemotherapy. Supplementary Figure S9: Combination of RMC-8839 and docetaxel is synergistic in KRAS^G13C^-driven NSCLC models.

Supplementary File 1 List of compound library screen drugs

Supplementary File 2 Cell line viability responses to compound library screen

Supplementary File 3 TCGA LUAD immune gene signatures based on *KRAS*-mutant genotype

## Data Availability

Compound library screen raw data are presented in Supplementary File S2. RAS-PM comutation patient numbers analysis breakdown is presented in Supplementary Table S3. TCGA Firehose Legacy immune gene signature analysis is presented in Supplementary File S3. All other data are available from the corresponding authors upon request.
