# Supplementary material for: Targeting KRAS Codon 13 Mutations Using Direct Combination Approaches in Non–Small Cell Lung Cancer
Source: Cancer Discov. 2026 Jun 22;16(11):2274–97. doi: 10.1158/2159-8290.CD-25-0884 (PMC13725345; doi:10.1158/2159-8290.CD-25-0884)
Supplement: Supplementary file 1 [file cd-25-0884_supplementary_figures_s1-s9_suppsf1.pdf]

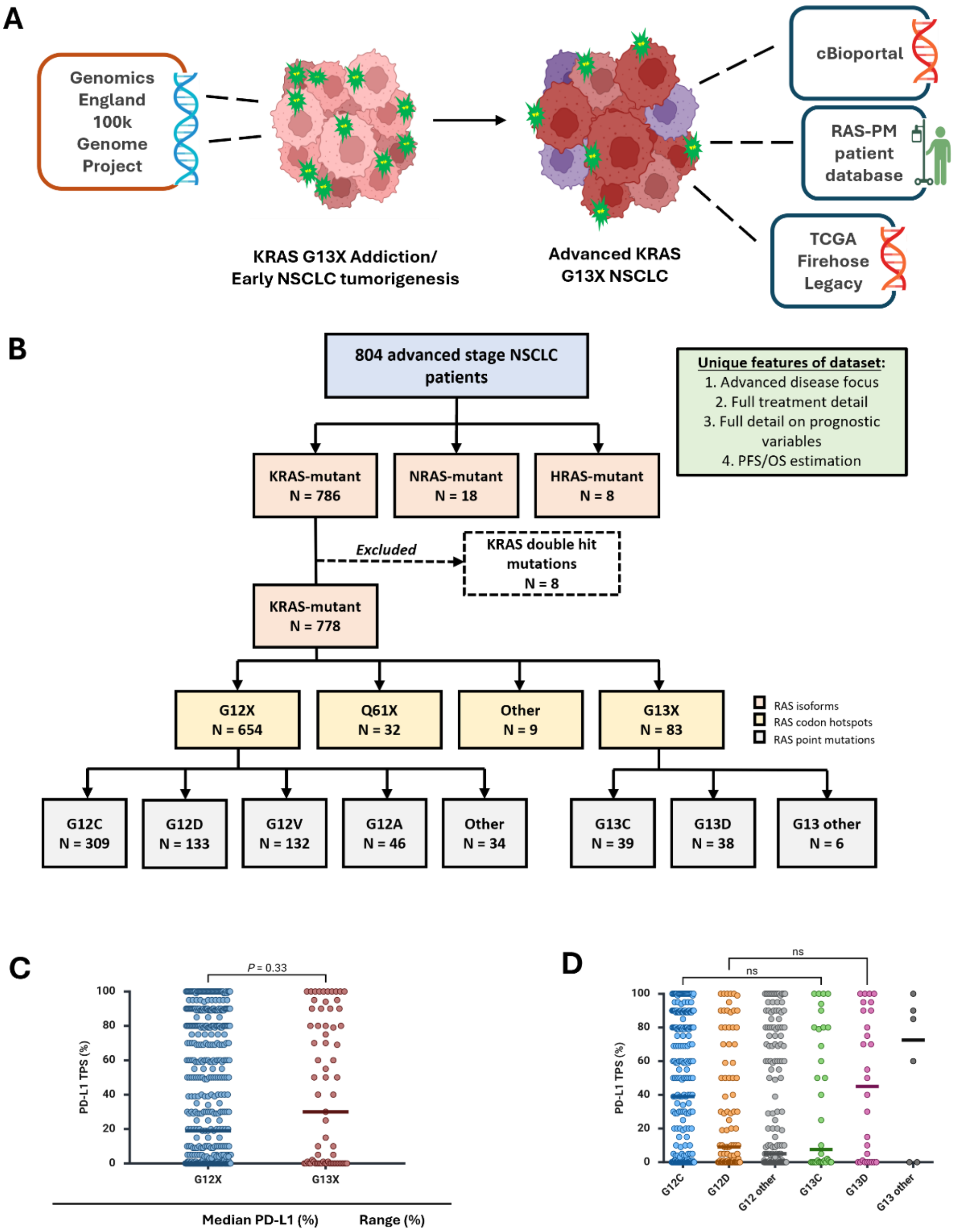

	Median PD-L1 (%)	Range (%)
G12X	19.0	0 – 100
G13X	30.0	0 - 100

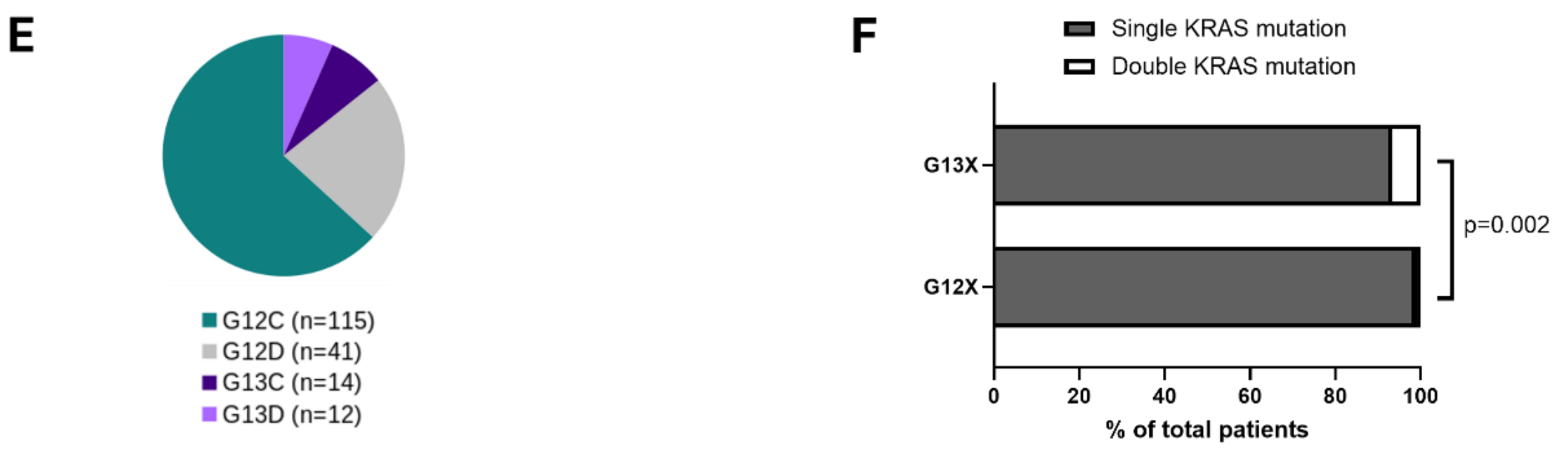

SUPPLEMENTARY FIGURE S1

Supplementary Figure S1: Analysis of prognostic variables and genomic features in *KRAS*-mutant NSCLC patient datasets

(A) Schematic illustrating the databases used to identify immunotherapy responses, pathogenic co-mutations and immune signatures associated with *KRAS*-mutant NSCLC in both early and advanced disease settings. Created with Biorender.
(B) Schematic highlighting the numbers of patients with specific RAS mutations that were enrolled in the RAS-PM database.
(C) Comparison of PL-D1 tumor proportion score (TPS) between $KRAS^{G12X}$ and $KRAS^{G13X}$ NSCLC patients from the RAS-PM database. Bar indicates median.
(D) Comparison of PL-D1 tumor proportion score (TPS) between $KRAS^{G12C}$, $KRAS^{G12D}$, $KRAS^{G12other}$, $KRAS^{G13C}$, $KRAS^{G13D}$ and $KRAS^{G13other}$ NSCLC patients from the RAS-PM database. Bar indicates median.
(E) The distribution of patients with different KRAS mutations that underwent whole genome sequencing as part of the 100K Genomes Project.
(F) The proportion of co-occuring KRAS mutations associated with $KRAS^{G12X}$ and $KRAS^{G13X}$ mutant patients from the RAS-PM database.

Statistical significance between comparisons calculated using an unpaired student's T-test, a one-way ANOVA (D) and a Fisher's exact test (F). *P<0.05, ns>0.05.

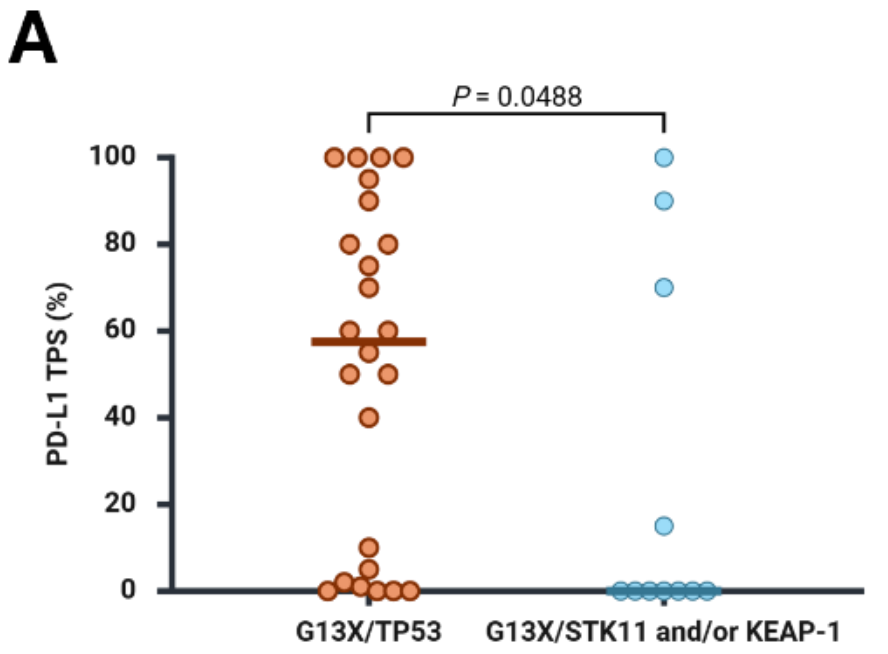

	Median PD-L1 (%)	Range (%)
G13X/TP53	57.5	0 – 100
G13X/STK11 and/or KEAP-1	0	0 - 100

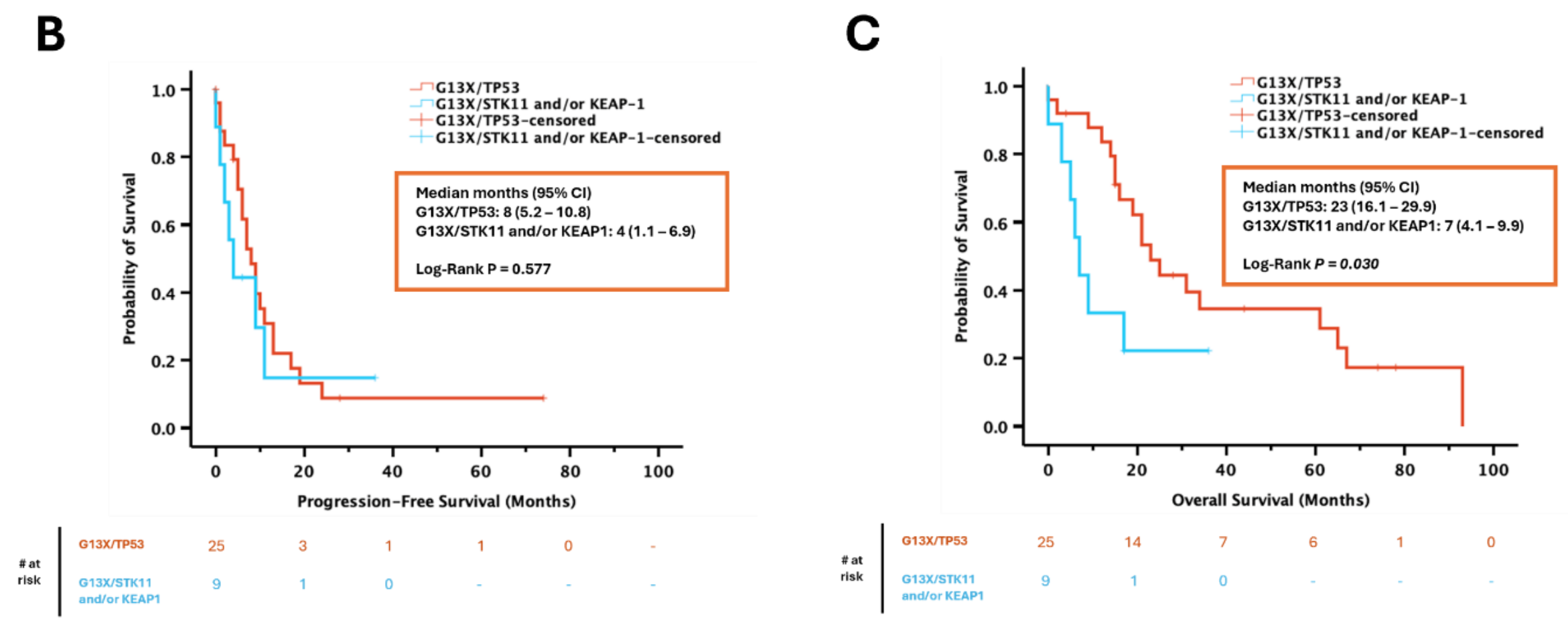

SUPPLEMENTARY FIGURE S2

Supplementary Figure S2: The impact of co-mutations on immunotherapy outcomes in *KRAS*^{G13X} NSCLC patients

(A) Comparison of PL-D1 tumor proportion score (TPS) between *KRAS*^{G13X} NSCLC patients divided into two subgroups: *TP53* mutation alone and *STK11* and/or *KEAP1* mutations. Data the RAS-PM database.

(B) Progression-free survival (PFS) of *KRAS*^{G13X} NSCLC patients receiving immunotherapy in all lines divided into two subgroups: *TP53* mutations alone and *STK11* and/or *KEAP1* mutations.

(C) Overall survival (OS) of *KRAS*^{G13X} NSCLC patients receiving immunotherapy in all lines divided into two subgroups: *TP53* mutations alone and *STK11* and/or *KEAP1* mutations.

Statistical significance between comparisons calculated using an unpaired student's T-test (A) and Log-rank (Mantel-Cox) test (B and C). *P<0.05, ns>0.05.

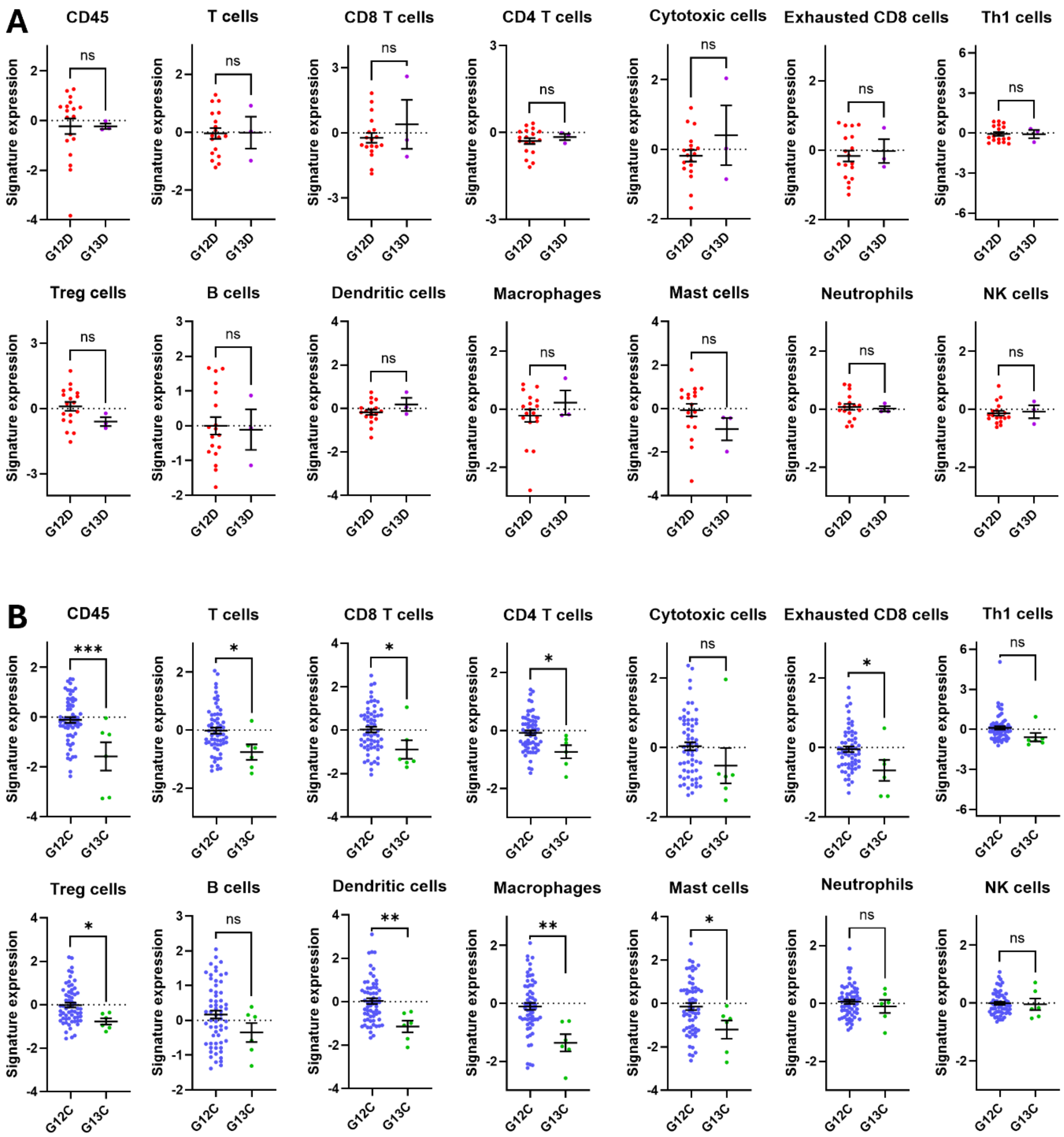

SUPPLEMENTARY FIGURE S3

Supplementary Figure S3: *KRAS*-mutant isoform-specific immune signatures from TCGA Firehose Legacy dataset

Comparison of immune gene signatures between (A) $KRAS^{G12D}$ and $KRAS^{G13D}$ NSCLC tumors and (B) $KRAS^{G12C}$ and $KRAS^{G13C}$ NSCLC tumors. Data taken from TCGA Firehose Legacy. Statistical significance between comparisons calculated using unpaired student's T-test.***P<0.001, **P<0.01 *P<0.05, ns>0.05.

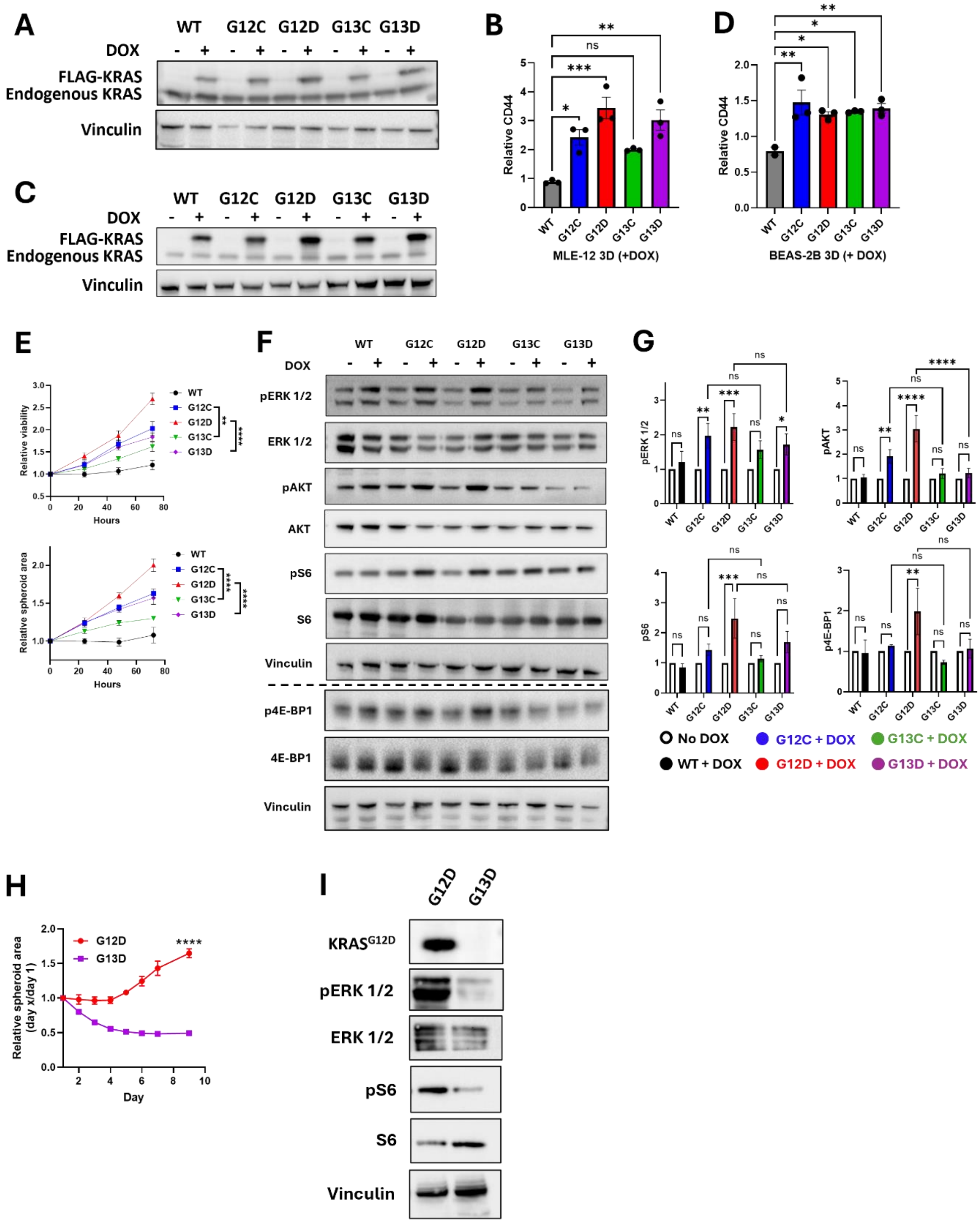

SUPPLEMENTARY FIGURE S4

Supplementary Figure S4: Analysis of *$KRAS^{G13X}$* oncogenicity using isogenic models

(A) Western blot analysis of FLAG-tagged KRAS in isogenic MLE-12 cells exposed to 100ng/mL doxycycline for 24h in media containing 0.5% FBS. Cells were plated in ULA conditions. Vinculin was used as a protein loading control.

(B) Surface CD44 levels of isogenic MLE-12 cells 24h after exposure to 100 ng/mL doxycycline (DOX) in medium containing 0.5% FBS and quantified by flow cytometry. Cells were plated in ULA conditions. Data normalized to untreated (no DOX) control. N=3 biological repeats per cell line; mean ± s.e.m.

(C) Western blot analysis of FLAG-tagged KRAS in isogenic BEAS-2B cells exposed to 100ng/mL DOX for 24h in medium containing 2.5% FBS. Cells were plated in ULA conditions. Vinculin was used as a protein loading control.

(D) Surface CD44 levels of isogenic BEAS-2B cells 24h after exposure to 100 ng/mL DOX in medium containing 2.5% FBS and quantified by flow cytometry. Cells were plated in ULA conditions. Data normalized to untreated (no DOX) control. N=2 (WT) or 3 (KRAS mutants) biological repeats per cell line; mean ± s.e.m.

(E) Relative viability (above) and spheroid area (below) of BEAS-2B cells over time after DOX-induced expression (100ng/mL) of each KRAS-mutant variant in medium containing 2.5% FBS. Cells were plated in ULA conditions. Viability was analyzed using CellTiter-Glo 3D and spheroid area measured using ImageJ. Data is expressed relative to untreated (no doxycycline) control. N=3 (WT) or 4 (KRAS mutants) biological repeats per cell line; mean ± s.e.m.

(F) Western blotting of BEAS-2B cells 24h after DOX-induced expression (100ng/mL) of each KRAS-mutant variant in medium containing 2.5% FBS. Cells were plated in ULA conditions. Data representative of 3-5 biological repeats per phospho-protein. Vinculin was used as a protein loading control. Dashed line indicates that representative blots from two different biological replicates are presented

(G) Quantification of (F). Phosphorylated protein and total protein run on separate blots were normalized to their respective loading controls on the same blot and then phospho-protein was normalized to the total protein. The data was then normalized to untreated (no DOX) control. Data normalized to untreated (no DOX) control. N=3-5 biological repeats per phospho-protein; mean ± s.e.m.

(H) Relative spheroid area of *$KRAS^{G12D}$* and *$KRAS^{G13D}$* MEFs over time measured using ImageJ. Cells were plated in ULA conditions. N=3 biological repeats per cell line; mean ± s.e.m.

(I) Western blotting of $KRAS^{G12D}$ and $KRAS^{G13D}$ MEFs 24h following seeding under ULA conditions. Data representative of 3 biological repeats. Vinculin was used as a protein loading control.

Statistical significance between comparisons calculated using a one-way ANOVA (B and D) and a two-way ANOVA (E, G and H). ****P<0.0001, ***P<0.001, **P<0.01, *P<0.05, ns>0.05.

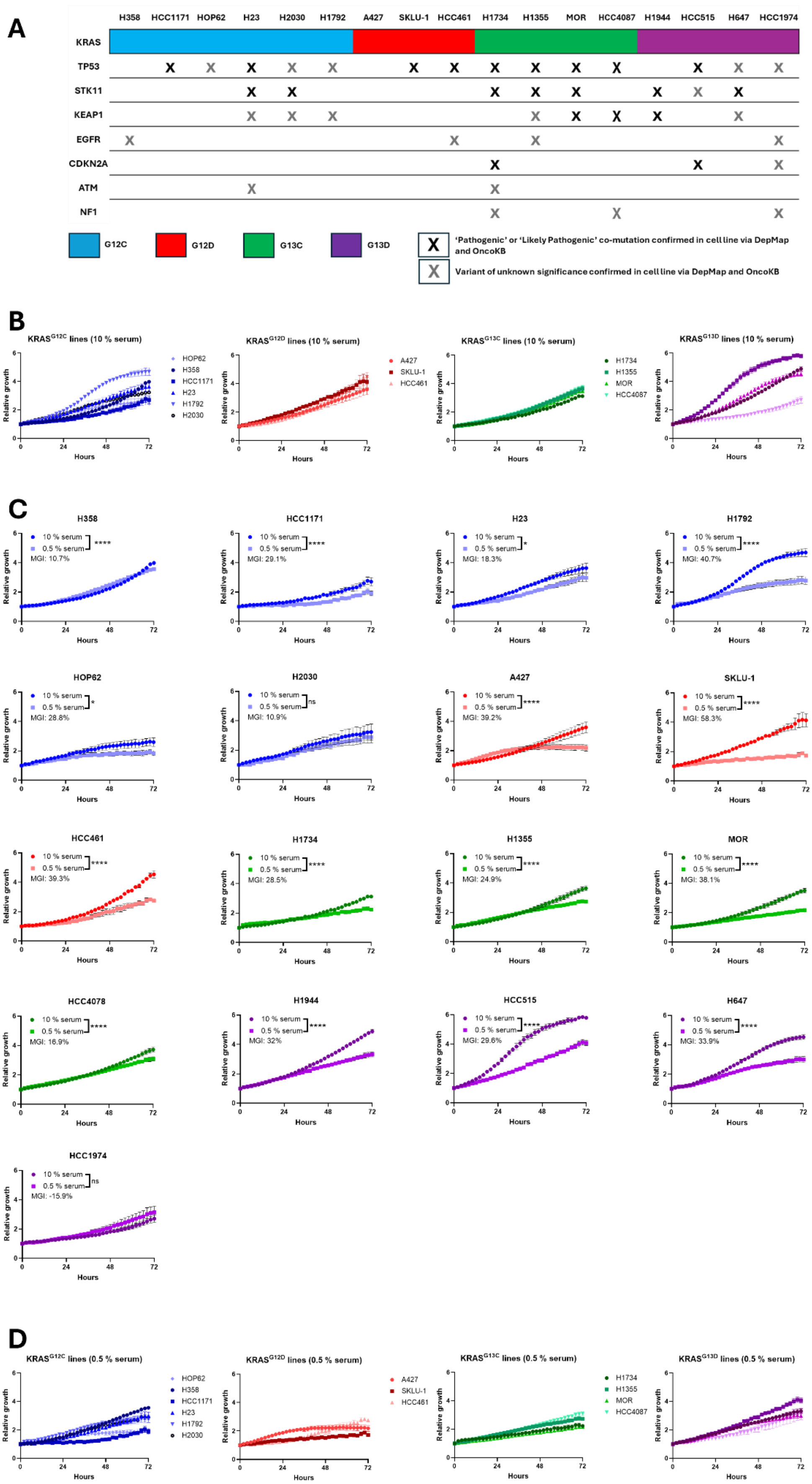

SUPPLEMENTARY FIGURE S5

107

Supplementary Figure S5: *KRAS*-mutant NSCLC cell line panel co-mutations and growth rate analyses

(A) Panel of advanced human NSCLC cell lines grouped by *KRAS*-mutant genotype and description of co-mutations for each cell line.

(B) Relative proliferation rates of advanced NSCLC cell lines cultured in medium containing 10% FBS. Each dot represents a timepoint which is normalized to 0hr. N=3 biological replicates per cell line; mean ± s.e.m.

(C) Relative proliferation rates of advanced NSCLC cell lines cultured in medium containing either 10% or 0.5% FBS. Each dot represents a timepoint which is normalized to 0hr. N=3 biological repeats per cell line per genotype; mean ± s.e.m. MGI = mean growth inhibition which is expressed as % reduction in proliferation between cells grown at 10% FBS and cells grown at 0.5% FBS at 72 hr timepoint. Blue = $KRAS^{G12C}$ lines; red = $KRAS^{G12D}$ lines; green = $KRAS^{G13C}$ lines; and purple = $KRAS^{G13D}$ lines. Statistical significance between comparisons calculated using a two-way ANOVA with significance at 72 hr reported. ****P<0.0001,*P<0.05, ns>0.05.

(D) Relative proliferation rates of advanced NSCLC cell lines cultured in medium containing 0.5% FBS. Each dot represents a timepoint which is normalized to 0hr. N=3 biological replicates per cell line; mean ± s.e.m.

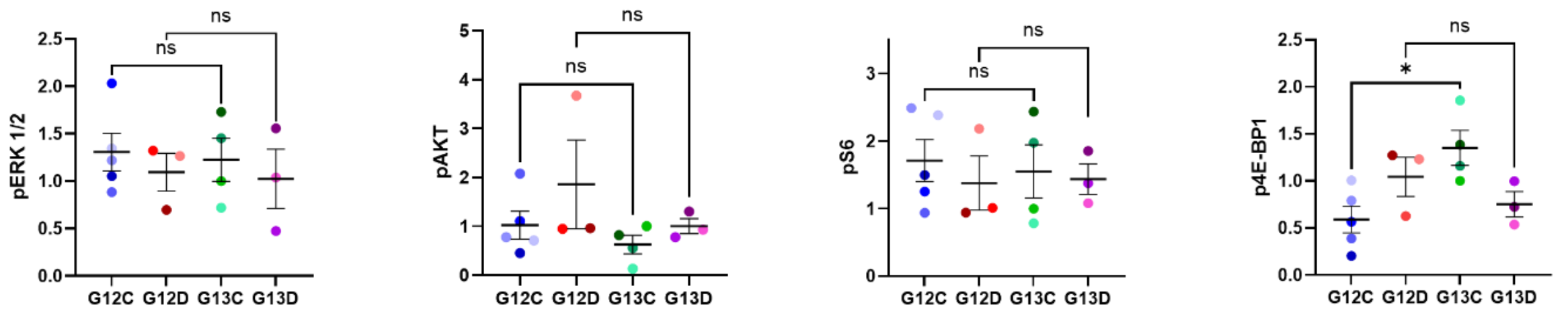

SUPPLEMENTARY FIGURE S6

Supplementary Figure S6: *KRAS*-mutant NSCLC cell line panel signaling

Quantification data from Figure 3D grouped by *KRAS*-mutant genotype. Statistical significance between comparisons calculated using a one-way ANOVA. *P<0.05, ns>0.05.

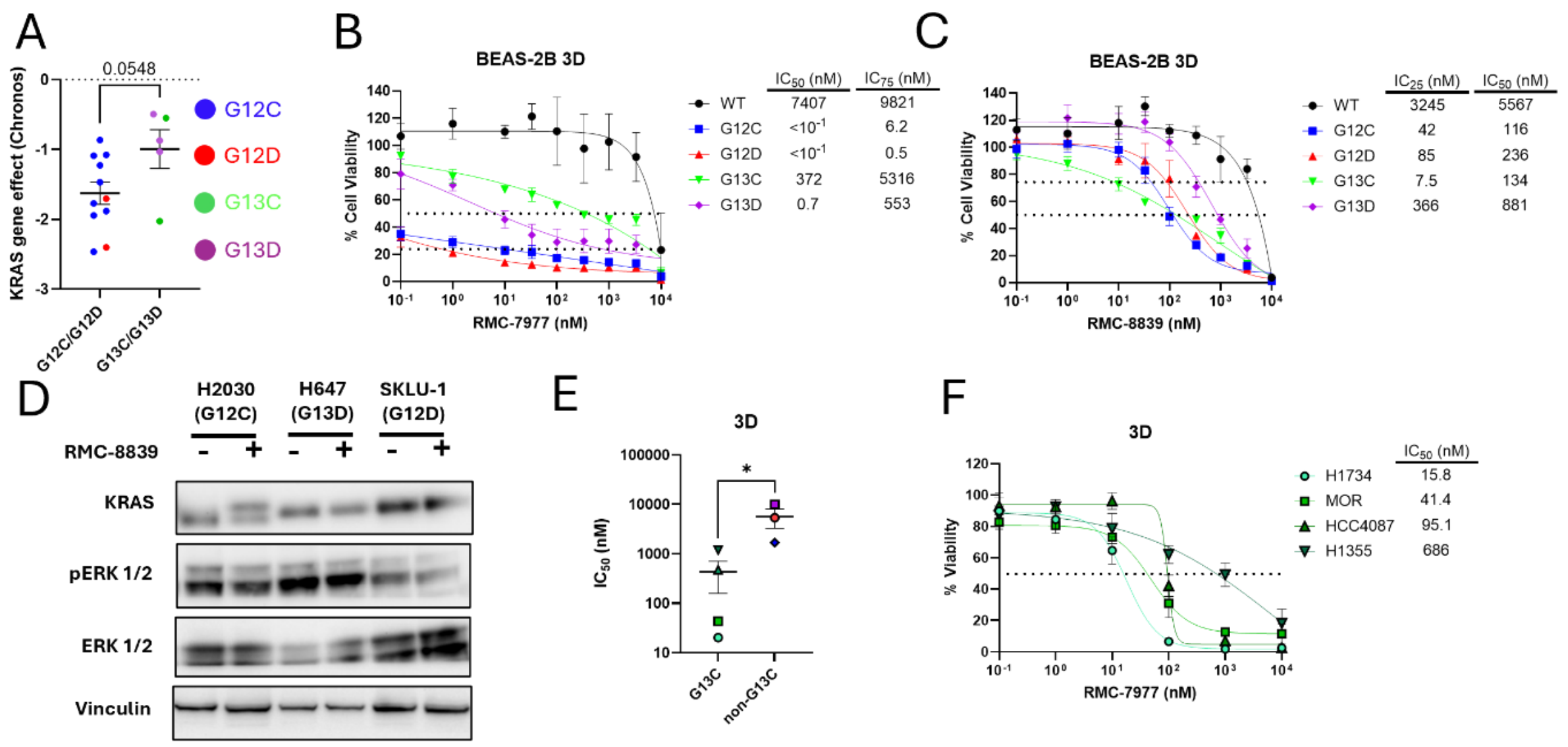

SUPPLEMENTARY FIGURE S7

Supplementary Figure S7: Analysis of (K)RAS (ON) inhibition in NSCLC models

(A) DepMap analysis of *KRAS* gene essentiality between $KRAS^{G12X}$ and $KRAS^{G13X}$ NSCLC cell lines using an CRISPR-Cas9 genetic screen.

(B) Dose response analysis of isogenic BEAS-2B cells previously treated with DOX for 24h to induce KRAS variant expression in 2.5% serum followed by 72h treatment with a concentration range of RMC-7977. Cells were plated in ULA conditions. Viability was analyzed by CellTiter-Glo 3D. Data expressed as a % of DMSO control. N=2 biological repeats per isogenic cell line; mean ± s.e.m. IC_{25} and IC_{50} values calculated for each cell line and presented as a table.

(C) Dose response analysis of isogenic BEAS-2B cells previously treated with DOX for 24h to induce KRAS variant expression in 2.5% serum followed by 72h treatment with a concentration range of RMC-8839. Cells were plated in ULA conditions. Viability was analyzed by CellTiter-Glo 3D. Data expressed as a % of DMSO control. N=3 biological repeats per isogenic cell line; mean ± s.e.m. IC_{25} and IC_{50} values calculated for each cell line and presented as a table.

(D) Western blotting of H2030 (*$KRAS^{G12C}$*), H647 (*$KRAS^{G13D}$*) and SKLU-1 (*$KRAS^{G12D}$*) NSCLC cell lines 24h after exposure to 100nM RMC-8839. Cells were plated in ULA conditions. Data representative of 3 biological repeats per cell line. Vinculin was used as a protein loading control.

(E) Comparison of IC_{50} values between *$KRAS^{G13C}$* and *$KRAS^{non\text{-}G13C}$* advanced cancer cell lines in response to RMC-8839. N=3 biological repeats per cell line; mean ± s.e.m.

(F) Dose response analysis *$KRAS^{G13C}$* advanced cancer cell lines exposed to increasing concentrations of RMC-7797 for 168h. Viability measured by CellTiter-Glo 3D. Data expressed as % of DMSO control. N=3-4 biological repeats per cell

line; mean ± s.e.m. IC_{50} values calculated for each cell line and presented as a table.

Statistical significance between comparisons calculated using an unpaired student's T-test (A and E). *$P<0.05$, ns>0.05.

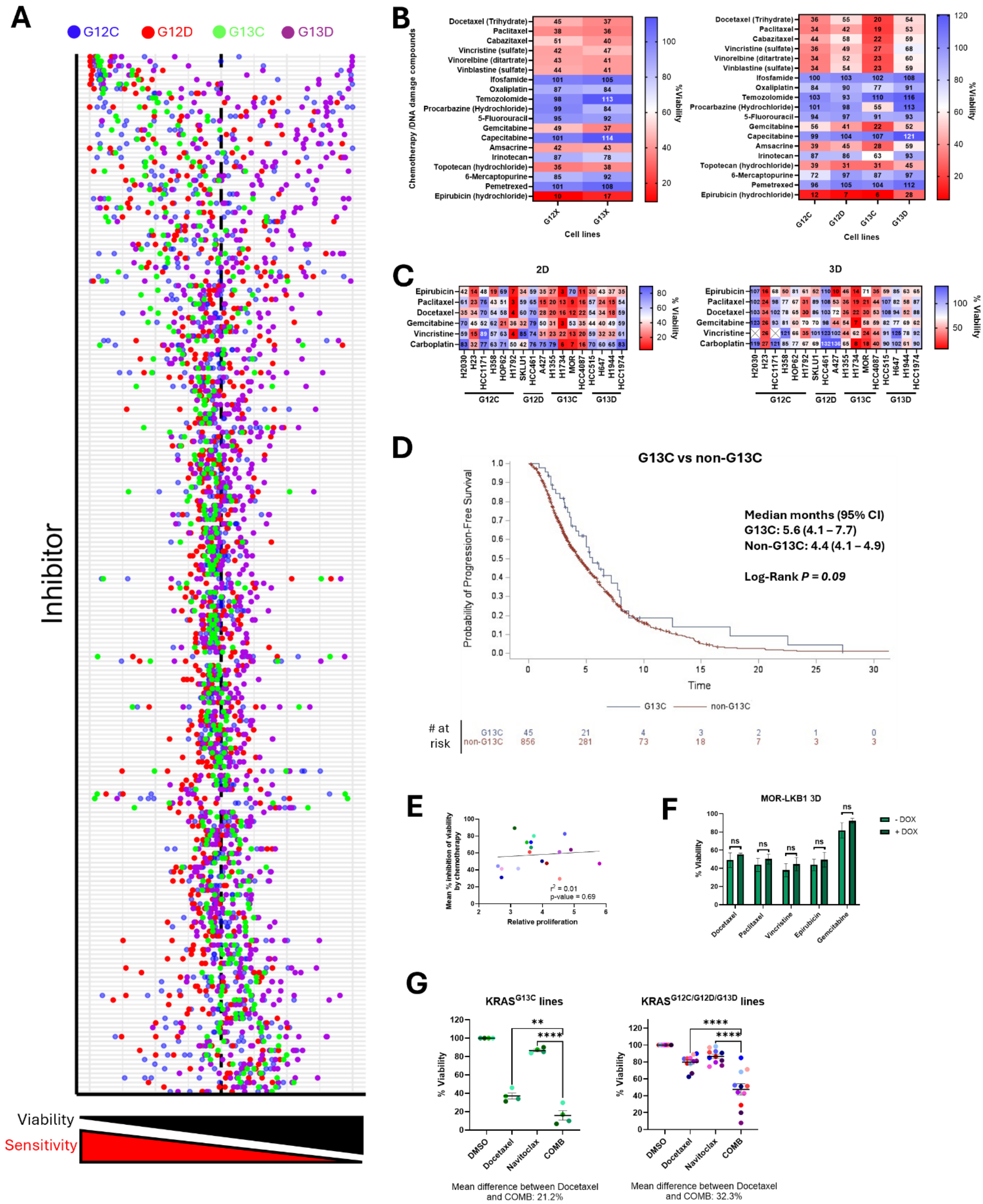

SUPPLEMENTARY FIGURE S8

Supplementary Figure S8: *KRAS*^{G13C}-driven NSCLC is selectively vulnerable to chemotherapy

(A) Relative sensitivity of advanced NSCLC cell lines to compound library screening. Each dot represents a cell line and is the mean of two repeats. Dashed line indicates midway point between the most resistant and the most sensitive cell line for every compound, with cell lines to the left of dashed line deemed drug-sensitive and cell lines to the right deemed drug-resistant. Each cell line is considered a genotype-specific biological replicate: *KRAS*^{G12C} (blue) – n=4 cell lines; *KRAS*^{G12D} (red) – n=3 cell lines; *KRAS*^{G13C} (green) – n=2 cell lines and *KRAS*^{G13D} (purple) – n=4 cell lines.

(B) Heatmap showing changes in viability in response to chemotherapy or DNA damaging compounds from the compound library screen in advanced *KRAS*-mutant NSCLC cell lines grouped by genotype (Left - *KRAS*^{G12X} (7 cell lines) and *KRAS*^{G13X} (6 cell lines); right - *KRAS*^{G12C} (4 cell lines), *KRAS*^{G12D} (3 cell lines), *KRAS*^{G13C} (2 cell lines) and *KRAS*^{G13D} (4 cell lines)). Viability was analyzed after 72h by CellTiter-Glo. Each value represents the mean change in % viability (relative to DMSO control) of different cell lines within that genotype.

(C) Heatmaps showing 2D (left) and 3D (right) viability of advanced cancer cell lines harboring *KRAS*^{G12C} (H358, H23, HCC1171, H2030, H1792 and HOP62), *KRAS*^{G12D} (SKLU-1, A427 and HCC461), *KRAS*^{G13C} (H1734, H1355, HCC4087 and MOR) and *KRAS*^{G13D} (H647, H1944, HCC515 and HCC1974) exposed to 1µM docetaxel, 1µM paclitaxel, 1µM vincristine, 1µM epirubicin, 100µM carboplatin and 1µM gemcitabine. Viability measured by CellTiter-Glo (3D). Data expressed as % of DMSO control. N=3 biological repeats; mean ± s.e.m for each cell line. Two KRAS^{G12C} lines (H2030 and HCC1171) excluded from vincristine response analysis due to viability increase of over 180% relative to DMSO control.

(D) Progression-free survival (PFS) between *KRAS*^{G13C} and *KRAS*$^{non\text{-}G13C}$ mutant NSCLC patients receiving 1st line chemotherapy. Data taken from the IFCT dataset.

(E) Correlation of *KRAS*-mutant NSCLC cell line relative growth at 72h with cell line-specific sensitivity to chemotherapy. N=3 biological repeats per cell line; mean ± s.e.m. Correlation determined using Pearson's algorithm. Each dot represents a cell line. For each cell line, the responses to all 6 chemotherapies were averaged. Blue = *KRAS*^{G12C} lines; red = *KRAS*^{G12D} lines; green = *KRAS*^{G13C} lines; and purple = *KRAS*^{G13D} lines.

(F) Viability of MOR-LKB1 exposed to 500 ng/mL doxycycline for 24h to induce LKB1 expression followed by exposure to 1µM docetaxel, 1µM paclitaxel, 1µM vincristine, 1µM epirubicin and 1µM gemcitabine. Viability measured by CellTiter-Glo (3D) 72 hr later. Data expressed as % of DMSO control. N=3 biological repeats; mean ± s.e.m.

(G) Viability of advanced cancer cell lines expressing (Left) KRAS^{G13C} and (Right) KRAS^{G12C}, KRAS^{G12D} and KRAS^{G13D} grouped together exposed to 1µM docetaxel or 100-1000nM navitoclax or both in combination. Viability measured by CellTiter-Glo (3D) 72 hr later. Data expressed as % of DMSO control. N=3 biological repeats

per cell line with the mean of two or three biological repeats shown as a dot. Error bars represent mean ± s.e.m of all cell lines within each condition.

Statistical significance between comparisons calculated using Log-rank (Mantel-Cox) (D), an unpaired student's T-test (F) and a one-way ANOVA (G). ****$P<0.0001$,**$P<0.01$, ns>0.05

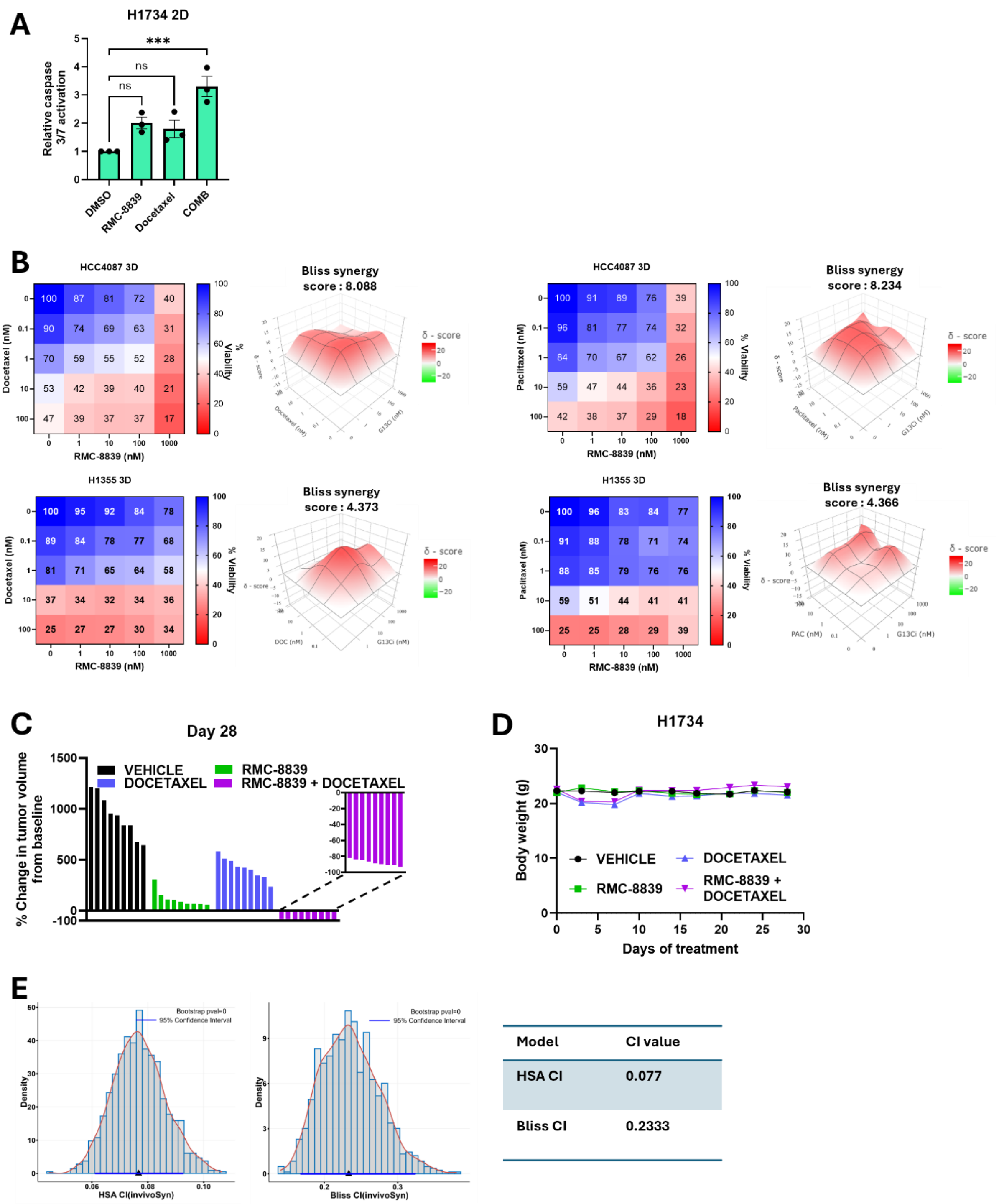

SUPPLEMENTARY FIGURE S9

Supplementary Figure S9: Combination of RMC-8839 and docetaxel is synergistic in *KRAS*^{G13C}-driven NSCLC models

(A) Relative caspase-3/-7 levels after exposure to RMC-8839, docetaxel and a combination of both for 24 hours. Cells were plated in ULA conditions. Data expressed relative DMSO control. N=3 biological repeats; mean ± s.e.m.

(B) HCC4087 and H1355 cells were exposed to increasing concentrations of $KRAS^{G13C}$ inhibition (RMC-8839) and either docetaxel and paclitaxel. Cells were plated in ULA conditions. 72h later, viability was analyzed by CellTiter-Glo (3D) and was expressed as a % of DMSO control. Drug interaction was calculated using Bliss Synergy (BS) Analysis. N=3 biological repeats per cell line; mean shown. BS less than -10 denotes antagonism; BS more than -10 but less than 10 denotes additivity and BS above 10 denotes synergy. In Bliss Synergy graphs, G13Ci indicates RMC-8839, DOC indicates docetaxel and PAC indicates paclitaxel.

(C) % change in tumor volume from baseline at day 28 in mice from the in vivo experiment described in Figure 6B.

(D) Body weights of mice from the in vivo experiment described in Figure 6B.

(E) In vivo drug synergy analysis of data from Figure 6B using InvivoSyn. (Left) Histograms showing the distribution of values for combination index (CI) under the highest single agent (HSA) model and the Bliss independence model with confidence internals and p values also reported. The black triangle identifies the overall CI value. (Right) The overall CI values under both models. CI <0 signifies synergy.

Statistical significance between comparisons calculated using a one-way ANOVA (A). ***P<0.001, ns>0.05
