# Supplementary material for: Targeting KRAS Codon 13 Mutations Using Direct Combination Approaches in Non–Small Cell Lung Cancer
Source: Cancer Discov. 2026 Jun 22;16(11):2274–97. doi: 10.1158/2159-8290.CD-25-0884 (PMC13725345; doi:10.1158/2159-8290.CD-25-0884)
Supplement: Supplementary file 2 [file cd-25-0884_supplementary_file_1_suppsd1.docx]

Supplementary File 1: Compounds used from HY-L022 FDA- approved drug library

| **Catalog Number** | **Product name** | **Class** |
| --- | --- | --- |
| HY-16513 | VAL-083 | chemotherapy/DNA damage |
| HY-17419 | Ifosfamide | chemotherapy/DNA damage |
| HY-13733 | Procarbazine (Hydrochloride) | chemotherapy/DNA damage |
| HY-B0015 | Paclitaxel | chemotherapy/DNA damage |
| HY-N0488 | Vincristine (sulfate) | chemotherapy/DNA damage |
| HY-17400 | Tegafur | chemotherapy/DNA damage |
| HY-105019A | Melflufen (hydrochloride) | chemotherapy/DNA damage |
| HY-13701 | Nelarabine | chemotherapy/DNA damage |
| HY-90006 | 5-Fluorouracil | chemotherapy/DNA damage |
| HY-17556 | Folinic acid | chemotherapy/DNA damage |
| HY-B0152 | Adenine | chemotherapy/DNA damage |
| HY-16562 | Irinotecan | chemotherapy/DNA damage |
| HY-15459 | Cabazitaxel | chemotherapy/DNA damage |
| HY-16398 | Pipobroman | chemotherapy/DNA damage |
| HY-13677 | 6-Mercaptopurine | chemotherapy/DNA damage |
| HY-13768A | Topotecan (hydrochloride) | chemotherapy/DNA damage |
| HY-B0021 | Doxifluridine | chemotherapy/DNA damage |
| HY-10821 | Raltitrexed | chemotherapy/DNA damage |
| HY-16503 | Treosulfan | chemotherapy/DNA damage |
| HY-17026 | Gemcitabine | chemotherapy/DNA damage |
| HY-B0016 | Capecitabine | chemotherapy/DNA damage |
| HY-10820 | Pemetrexed | chemotherapy/DNA damage |
| HY-12279 | Umbralisib | chemotherapy/DNA damage |
| HY-13624A | Epirubicin (hydrochloride) | chemotherapy/DNA damage |
| HY-13585 | Carmustine | chemotherapy/DNA damage |
| HY-10446 | Pralatrexate | chemotherapy/DNA damage |
| HY-B0078 | Dacarbazine | chemotherapy/DNA damage |
| HY-13727A | Pixantrone | chemotherapy/DNA damage |
| HY-13669 | Lomustine | chemotherapy/DNA damage |
| HY-B0011A | Docetaxel (Trihydrate) | chemotherapy |
| HY-17364 | Temozolomide | chemotherapy/DNA damage |
| HY-A0004 | Decitabine | chemotherapy/DNA damage |
| HY-B0069 | Fludarabine | chemotherapy/DNA damage |
| HY-13551 | Amsacrine | chemotherapy/DNA damage |
| HY-13761 | Teniposide | chemotherapy/DNA damage |
| HY-13703A | Nimustine (hydrochloride) | chemotherapy/DNA damage |
| HY-13593 | Chlorambucil | chemotherapy/DNA damage |
| HY-12053A | Vinorelbine (ditartrate) | chemotherapy/DNA damage |
| HY-B0181 | Altretamine | chemotherapy/DNA damage |
| HY-13567 | Bendamustine | chemotherapy/DNA damage |
| HY-13780 | Vinblastine (sulfate) | chemotherapy/DNA damage |
| HY-13566A | Belotecan (hydrochloride) | chemotherapy/DNA damage |
| HY-13667 | Levoleucovorin (Calcium) | chemotherapy/DNA damage |
| HY-17371 | Oxaliplatin | chemotherapy/DNA damage |
| HY-A0063A | Tipiracil | chemotherapy/DNA damage |
| HY-10159 | Nilotinib | TKI |
| HY-10230 | Midostaurin | TKI |
| HY-12297 | Sulfatinib | TKI |
| HY-12798A | Netarsudil (dimesylate) | TKI |
| HY-N0110 | Palmatine (chloride) | TKI |
| HY-15164A | Icotinib | TKI |
| HY-12215 | Lorlatinib | TKI |
| HY-10261 | Afatinib | TKI |
| HY-10209 | Masitinib | TKI |
| HY-15307A | Belumosudil (mesylate) | TKI |
| HY-15728 | Radotinib | TKI |
| HY-10997 | Ibrutinib | TKI |
| HY-18300 | Filgotinib | TKI |
| HY-14660 | Dabrafenib | TKI |
| HY-15463 | Imatinib | TKI |
| HY-10961 | Momelotinib | TKI |
| HY-10208 | Pazopanib | TKI |
| HY-10201 | Sorafenib | TKI |
| HY-101474A | Zanubrutinib | TKI |
| HY-15666 | Olverembatinib | TKI |
| HY-13404 | Capmatinib | TKI |
| HY-13001 | Quizartinib | TKI |
| HY-16379 | Pacritinib | TKI |
| HY-12279C | Umbralisib (hydrochloride) | TKI |
| HY-13064 | Cobimetinib | TKI |
| HY-13904 | Flumatinib | TKI |
| HY-10999 | Trametinib | TKI |
| HY-15202 | Binimetinib | TKI |
| HY-18708 | Erdafitinib | TKI |
| HY-10065 | Axitinib | TKI |
| HY-13016 | Cabozantinib | TKI |
| HY-109061 | Lazertinib | TKI |
| HY-13911 | Hydroxyfasudil | TKI |
| HY-12866 | Larotrectinib | TKI |
| HY-12678 | Entrectinib | TKI |
| HY-12057 | Vemurafenib | TKI |
| HY-10255A | Sunitinib | TKI |
| HY-I0960 | Uracil | TKI |
| HY-50904 | Nintedanib | TKI |
| HY-19912 | Fruquintinib | TKI |
| HY-100818 | Futibatinib | TKI |
| HY-10981 | Lenvatinib | TKI |
| HY-50858 | Ruxolitinib (phosphate) | TKI |
| HY-13272 | Dacomitinib | TKI |
| HY-B1305 | Chloropyramine hydrochloride | TKI |
| HY-10158 | Bosutinib | TKI |
| HY-103022 | Repotrectinib | TKI |
| HY-50706 | Selumetinib | TKI |
| HY-13038 | Fostamatinib Disodium | TKI |
| HY-17600 | Acalabrutinib | TKI |
| HY-16749A | Pexidartinib (hydrochloride) | TKI |
| HY-19730 | Olmutinib | TKI |
| HY-N0281 | Daphnetin | TKI |
| HY-101561 | Avapritinib | TKI |
| HY-50895 | Gefitinib | TKI |
| HY-131328 | Pirtobrutinib | TKI |
| HY-10342 | Enzastaurin | TKI |
| HY-16069 | Tucatinib | TKI |
| HY-135815 | Mobocertinib | TKI |
| HY-13011 | Alectinib | TKI |
| HY-B0719 | Ingenol Mebutate | TKI |
| HY-10409 | Fedratinib | TKI |
| HY-10260 | Vandetanib | TKI |
| HY-10999A | Trametinib (DMSO solvate) | TKI |
| HY-15431 | Capivasertib | TKI |
| HY-109099 | Pemigatinib | TKI |
| HY-50878 | Crizotinib | TKI |
| HY-129390 | Orelabrutinib | TKI |
| HY-13026 | Idelalisib | TKI |
| HY-112301 | Pralsetinib | TKI |
| HY-15244 | Alpelisib | TKI |
| HY-32721 | Neratinib | TKI |
| HY-112823 | Almonertinib | TKI |
| HY-N0060 | Ferulic acid | TKI |
| HY-50898 | Lapatinib | TKI |
| HY-50896 | Erlotinib | TKI |
| HY-17044 | Duvelisib | TKI |
| HY-13311 | Infigratinib | TKI |
| HY-10340 | Tirbanibulin | TKI |
| HY-112306 | Ripretinib | TKI |
| HY-10331 | Regorafenib | TKI |
| HY-15959 | Savolitinib | TKI |
| HY-15605 | Encorafenib | TKI |
| HY-101820 | Simotinib | TKI |
| HY-103714A | Ensartinib (dihydrochloride) | TKI |
| HY-15772 | Osimertinib | TKI |
| HY-10977 | Tivozanib | TKI |
| HY-114370 | Selpercatinib | TKI |
| HY-12047 | Ponatinib | TKI |
| HY-104010 | Asciminib | TKI |
| HY-10341 | Fasudil (Hydrochloride) | TKI |
| HY-10181 | Dasatinib | TKI |
| HY-15346A | Copanlisib (dihydrochloride) | TKI |
| HY-12432 | Gilteritinib | TKI |
| HY-12857 | Brigatinib | TKI |
| HY-10330 | Toceranib | TKI |
| HY-N1373 | Sophoridine | Apoptosis |
| HY-107324 | ?-Elemene | Apoptosis |
| HY-50935 | Troglitazone | Apoptosis |
| HY-19696A | Tauroursodeoxycholate (sodium) | Apoptosis |
| HY-13680 | Meisoindigo | Apoptosis |
| HY-N0124 | Dioscin | Apoptosis |
| HY-15531 | Venetoclax | Apoptosis |
| HY-N0714A | Berbamine (dihydrochloride) | Apoptosis |
| HY-10452 | Ixazomib citrate | Apoptosis |
| HY-B0313 | Hydroxyurea | Apoptosis |
| HY-13407 | Gossypol | Apoptosis |
| HY-14944 | Homoharringtonine | Apoptosis |
| HY-10219 | Rapamycin | autophagy |
| HY-10218 | Everolimus | autophagy |
| HY-B2098 | Lucanthone | autophagy |
| HY-A0061 | Trifluridine | autophagy |
| HY-50856 | Ruxolitinib | autophagy |
| HY-50767C | Palbociclib (hydrochloride) | cell cycle |
| HY-16209 | Forodesine (hydrochloride) | cell cycle/ DNA damage |
| HY-13674 | Maytansine | cell cycle/ DNA damage |
| HY-129071 | Vindesine (sulfate) | cell cycle/ DNA damage |
| HY-16210 | Forodesine | cell cycle/ DNA damage |
| HY-10492 | Dinaciclib | cell cycle |
| HY-15777 | Ribociclib | cell cycle |
| HY-10617A | Rucaparib | DNA damage |
| HY-17469 | Gimeracil | DNA damage |
| HY-10162 | Olaparib | DNA damage |
| HY-10619 | Niraparib | DNA damage |
| HY-104044 | Pamiparib | DNA damage |
| HY-16106 | Talazoparib | DNA damage |
| HY-12137 | Volasertib | cell cycle |
| HY-12009 | Pazopanib (Hydrochloride) | DNA damage |
| HY-114778 | Fluzoparib | DNA damage |
| HY-16297A | Abemaciclib | cell cycle |
| HY-109108 | Valemetostat | epigenetics |
| HY-18690 | Enasidenib | epigenetics |
| HY-13322 | Pracinostat | epigenetics |
| HY-12164 | Mocetinostat | epigenetics |
| HY-10225 | Belinostat | epigenetics |
| HY-10224 | Panobinostat | epigenetics |
| HY-13426 | Roxadustat | epigenetics |
| HY-109015 | Tucidinostat | epigenetics |
| HY-B1625 | Deferoxamine | hypoxia |
| HY-N0523 | Gallic acid | hypoxia |
| HY-N0733 | Glucosamine (hydrochloride) | hypoxia |
| HY-125840 | Belzutifan | hypoxia |
| HY-17363 | Dimethyl fumarate | hypoxia |
| HY-18258 | Berberine (chloride) | hypoxia |
| HY-N0193 | Artesunate | hypoxia |
| HY-B1640 | Ethacrynic acid | hypoxia |
| HY-14649 | Retinoic acid | hypoxia |
| HY-N0018 | Daidzin | metabolism |
| HY-B0627 | Metformin | metabolism |
| HY-B0364A | Dyclonine (hydrochloride) | metabolism |
| HY-16500 | Tolrestat | metabolism |
| HY-76847 | Chenodeoxycholic Acid | metabolism |
| HY-B0589 | Atorvastatin | metabolism |
| HY-B0144A | Pitavastatin | metabolism |
| HY-B0240 | Disulfiram | metabolism |
| HY-N0198 | Nordihydroguaiaretic acid | metabolism |
| HY-17504 | Rosuvastatin (Calcium) | metabolism |
| HY-N0164 | Matrine | metabolism |
| HY-B0218 | Orlistat | metabolism |
| HY-114226 | Olutasidenib | metabolism |
| HY-18767 | Ivosidenib | metabolism |
| HY-B0808 | Oxaprozin | NFKB |
| HY-B0167A | Sodium Salicylate | NFKB |
| HY-15122 | Sinomenine | NFKB |
| HY-32735 | Triptolide | NFKB |
| HY-N0510 | Aristolochic acid A | NFKB |
| HY-N0052A | Sanguinarine (chloride) | NFKB |
| HY-114277 | Sotorasib | other |
| HY-17536 | Selinexor | other |
| HY-B0667 | Balsalazide | other |
| HY-B0146 | Verteporfin | other |
| HY-15373 | Fenretinide | other |
| HY-N0196 | Baicalein | other |
| HY-N0156 | Oleanolic Acid | other |
| HY-130149 | Adagrasib | other |
| HY-15136 | Lonafarnib | other |
| HY-10453 | Ixazomib | proteasome |
| HY-10227 | Bortezomib | proteasome |
| HY-B0380A | Trimebutine (maleate) | stemness |
| HY-13748 | Silybin A | stemness |
| HY-16582A | Sonidegib | stemness/ WNT |
| HY-16582 | Sonidegib (diphosphate) | stemness/ WNT |
| HY-16391 | Glasdegib | stemness/ WNT |
