# Supplementary material for: Targeting KRAS Codon 13 Mutations Using Direct Combination Approaches in Non–Small Cell Lung Cancer
Source: Cancer Discov. 2026 Jun 22;16(11):2274–97. doi: 10.1158/2159-8290.CD-25-0884 (PMC13725345; doi:10.1158/2159-8290.CD-25-0884)
Supplement: Supplementary file 3 [file cd-25-0884_supplementary_file_2_suppsd2.docx]

| Catalog Number | Product name | Class | Dose (uM) | H2030 | H23 | HCC1171 | H1792 | SKLU1 | A427 | HCC461 | H1355 | H1734 | HCC515 | H647 | H1944 | HCC1974 |
| --- | --- | --- | --- | --- | --- | --- | --- | --- | --- | --- | --- | --- | --- | --- | --- | --- |
| HY-16513 | VAL-083 | chemotherapy/DNA damage | 1.667 | 86.6754 | 90.43339 | 95.6901181 | 75.190363 | 63.10236 | 90.07367 | 99.70299 | 94.64629 | 53.87877 | 91.41374 | 115.285 | 76.15 | 124.445 |
| HY-17419 | Ifosfamide | chemotherapy/DNA damage | 1.667 | 97.9382 | 99.64514 | 100.342933 | 101.63234 | 84.88829 | 118.8854 | 104.4478 | 100.0282 | 104.0006 | 102.6456 | 90.6385 | 104.02 | 133.3651 |
| HY-13733 | Procarbazine (Hydrochloride) | chemotherapy/DNA damage | 1.667 | 109.8279 | 105.3274 | 96.3825588 | 102.60598 | 94.50806 | 140.147 | 102.1062 | 107.2404 | 114.1413 | 105.6096 | 101.5484 | 104.9597 | 136.8585 |
| HY-B0015 | Paclitaxel | chemotherapy/DNA damage | 1.667 | 50.856 | 17.31692 | 63.0573105 | 2.9895002 | 48.06037 | 23.76624 | 54.9895 | 18.26282 | 19.12822 | 73.43884 | 40.10005 | 26.61369 | 69.93587 |
| HY-N0488 | Vincristine (sulfate) | chemotherapy/DNA damage | 1.667 | 80.66263 | 11.05633 | 49.6207174 | 2.8388733 | 47.60384 | 31.17652 | 67.01925 | 20.46767 | 32.54857 | 72.37228 | 88.71112 | 31.50502 | 80.69118 |
| HY-17400 | Tegafur | chemotherapy/DNA damage | 1.667 | 111.876 | 102.3205 | 101.342876 | 111.00066 | 93.65027 | 112.2262 | 98.9899 | 102.4613 | 130.84 | 103.7751 | 129.155 | 111.367 | 136.0822 |
| HY-105019A | Melflufen (hydrochloride) | chemotherapy/DNA damage | 1.667 | 99.0527 | 11.30206 | 41.0023037 | 21.888029 | 7.732699 | 0.020586 | 27.47324 | 55.46799 | 3.918431 | 40.35207 | 80.64608 | 55.49029 | 27.85227 |
| HY-13701 | Nelarabine | chemotherapy/DNA damage | 1.667 | 105.4402 | 100.3664 | 101.592816 | 106.87405 | 98.65978 | 120.8206 | 100.3167 | 107.8955 | 124.453 | 108.2149 | 121.4844 | 101.7717 | 128.8955 |
| HY-90006 | 5-Fluorouracil | chemotherapy/DNA damage | 1.667 | 113.372 | 93.3624 | 96.367155 | 72.765354 | 77.3293 | 110.2494 | 102.518 | 78.01138 | 104.6987 | 82.50203 | 112.1362 | 68.46623 | 108.7285 |
| HY-17556 | Folinic acid | chemotherapy/DNA damage | 1.667 | 114.5785 | 88.52417 | 103.824831 | 108.9483 | 96.47213 | 106.6294 | 108.8545 | 102.5985 | 109.3978 | 100.855 | 111.2921 | 103.6312 | 151.8418 |
| HY-B0152 | Adenine | chemotherapy/DNA damage | 1.667 | 112.7722 | 99.04301 | 106.138343 | 105.59007 | 100.9682 | 124.9865 | 105.8615 | 101.2907 | 119.6029 | 113.9364 | 120.514 | 111.4884 | 154.1113 |
| HY-16562 | Irinotecan | chemotherapy/DNA damage | 1.667 | 90.43197 | 92.50718 | 87.1686897 | 78.513937 | 54.06036 | 105.3424 | 99.84421 | 96.54921 | 29.37536 | 69.99773 | 104.3436 | 82.44201 | 113.3891 |
| HY-15459 | Cabazitaxel | chemotherapy/DNA damage | 1.667 | 61.28575 | 31.6399 | 75.2940755 | 6.1207538 | 55.96666 | 48.44727 | 69.60244 | 23.95868 | 20.05566 | 73.59017 | 57.1188 | 34.17884 | 70.64219 |
| HY-16398 | Pipobroman | chemotherapy/DNA damage | 1.667 | 112.0629 | 114.4762 | 101.71075 | 120.34035 | 101.3258 | 130.2454 | 101.3297 | 100.434 | 109.825 | 105.5665 | 145.2078 | 98.53248 | 123.4114 |
| HY-13677 | 6-Mercaptopurine | chemotherapy/DNA damage | 1.667 | 95.98304 | 62.35391 | 100.981816 | 30.443296 | 88.9423 | 105.4086 | 96.07003 | 66.9331 | 107.5188 | 102.9179 | 115.268 | 73.65027 | 94.75128 |
| HY-13768A | Topotecan (hydrochloride) | chemotherapy/DNA damage | 1.667 | 51.66309 | 38.29004 | 40.5206725 | 25.513493 | 10.71087 | 35.02317 | 46.4958 | 59.58493 | 1.970398 | 39.66041 | 63.20213 | 37.8046 | 39.20268 |
| HY-B0021 | Doxifluridine | chemotherapy/DNA damage | 1.667 | 109.1253 | 111.4718 | 102.274049 | 117.324 | 96.40938 | 122.4899 | 101.2813 | 85.4977 | 121.3173 | 85.24103 | 135.849 | 95.4685 | 125.8142 |
| HY-10821 | Raltitrexed | chemotherapy/DNA damage | 1.667 | 99.17164 | 114.3093 | 109.197328 | 60.363763 | 90.19129 | 134.8959 | 109.7476 | 67.30954 | 113.497 | 96.89106 | 115.1879 | 87.55201 | 115.773 |
| HY-16503 | Treosulfan | chemotherapy/DNA damage | 1.667 | 108.5322 | 94.62865 | 113.686393 | 101.25002 | 88.02909 | 121.9971 | 106.3422 | 99.2186 | 100.0848 | 103.4267 | 123.136 | 89.12821 | 136.8522 |
| HY-17026 | Gemcitabine | chemotherapy/DNA damage | 1.667 | 116.7981 | 30.13209 | 47.5315692 | 30.665358 | 21.56489 | 50.75824 | 49.94193 | 37.24513 | 6.143881 | 53.35491 | 60.93125 | 32.49829 | 60.41346 |
| HY-B0016 | Capecitabine | chemotherapy/DNA damage | 1.667 | 103.8433 | 94.4673 | 100.956335 | 95.53687 | 96.10871 | 119.3649 | 96.32724 | 96.06507 | 117.8777 | 123.5226 | 120.3231 | 109.8981 | 129.1829 |
| HY-10820 | Pemetrexed | chemotherapy/DNA damage | 1.667 | 107.0781 | 96.85787 | 109.324268 | 70.326821 | 93.68094 | 115.4065 | 107.1269 | 84.57974 | 122.5793 | 106.3194 | 126.1431 | 90.07366 | 124.8545 |
| HY-12279 | Umbralisib | chemotherapy/DNA damage | 1.667 | 103.1874 | 103.1455 | 107.02656 | 99.68113 | 98.36131 | 118.5216 | 101.8389 | 101.0233 | 127.9447 | 87.8694 | 108.1456 | 104.5127 | 122.7429 |
| HY-13624A | Epirubicin (hydrochloride) | chemotherapy/DNA damage | 1.667 | 9.424573 | 1.92311 | 34.7518646 | 1.6943542 | 9.533091 | 0.323196 | 11.52187 | 9.036636 | 1.99097 | 42.35477 | 48.28091 | 2.462715 | 19.8031 |
| HY-13585 | Carmustine | chemotherapy/DNA damage | 1.667 | 103.9764 | 104.064 | 102.178494 | 106.86573 | 104.5069 | 132.0634 | 119.4378 | 95.61273 | 103.7939 | 120.7571 | 105.706 | 100.056 | 117.0956 |
| HY-10446 | Pralatrexate | chemotherapy/DNA damage | 1.667 | 94.60158 | 48.1637 | 97.7438204 | 43.795621 | 84.42941 | 100.3838 | 103.2349 | 59.83616 | 91.41066 | 104.9115 | 96.81499 | 76.07743 | 61.62592 |
| HY-B0078 | Dacarbazine | chemotherapy/DNA damage | 1.667 | 100.5292 | 104.9856 | 95.4885837 | 107.74711 | 91.738 | 122.1934 | 105.4388 | 105.5523 | 107.4289 | 104.751 | 119.0769 | 105.952 | 135.0356 |
| HY-13727A | Pixantrone | chemotherapy/DNA damage | 1.667 | 78.25151 | 59.49205 | 50.5745724 | 39.252542 | 41.39978 | 38.27346 | 67.42441 | 59.10579 | 26.85621 | 66.0536 | 81.23406 | 63.10549 | 87.42122 |
| HY-13669 | Lomustine | chemotherapy/DNA damage | 1.667 | 99.44437 | 110.333 | 91.6104094 | 109.01985 | 93.34255 | 114.9857 | 119.0041 | 94.08783 | 115.6515 | 98.61612 | 110.9069 | 101.5113 | 123.4508 |
| HY-B0011A | Docetaxel (Trihydrate) | chemotherapy | 1.667 | 50.76196 | 22.44761 | 65.6331222 | 4.5314448 | 53.3708 | 39.42541 | 72.31896 | 19.97927 | 19.76576 | 69.01755 | 47.91038 | 30.54333 | 70.42066 |
| HY-17364 | Temozolomide | chemotherapy/DNA damage | 1.667 | 103.8188 | 97.64656 | 97.4393951 | 113.15194 | 89.276 | 79.65145 | 109.7896 | 104.2205 | 116.4639 | 108.7055 | 126.3368 | 103.7635 | 125.837 |
| HY-A0004 | Decitabine | chemotherapy/DNA damage | 1.667 | 100.3754 | 83.49722 | 105.365527 | 85.611599 | 69.94547 | 61.3232 | 88.51126 | 83.95949 | 91.61573 | 103.2253 | 141.5704 | 86.02496 | 78.66743 |
| HY-B0069 | Fludarabine | chemotherapy/DNA damage | 1.667 | 102.5103 | 107.1455 | 99.9309474 | 78.0348 | 77.95629 | 83.94623 | 80.35537 | 92.35438 | 93.57498 | 89.83298 | 110.109 | 100.6173 | 107.305 |
| HY-13551 | Amsacrine | chemotherapy/DNA damage | 1.667 | 51.64617 | 36.3263 | 48.0871916 | 19.246967 | 22.872 | 38.78197 | 73.33832 | 36.55274 | 19.65255 | 65.29245 | 65.45145 | 43.16237 | 61.07874 |
| HY-13761 | Teniposide | chemotherapy/DNA damage | 1.667 | 53.22643 | 19.32695 | 47.9057589 | 17.217765 | 19.60172 | 23.39051 | 59.00277 | 38.80168 | 17.5011 | 79.49649 | 75.87641 | 40.92801 | 52.74089 |
| HY-13703A | Nimustine (hydrochloride) | chemotherapy/DNA damage | 1.667 | 97.48584 | 103.1627 | 101.814867 | 94.976508 | 94.609 | 106.1705 | 92.15381 | 97.08918 | 101.4153 | 108.0692 | 116.03 | 99.03492 | 106.4707 |
| HY-13593 | Chlorambucil | chemotherapy/DNA damage | 1.667 | 96.83109 | 99.24652 | 95.2652533 | 98.542224 | 94.77357 | 96.78889 | 92.30222 | 96.13359 | 106.5865 | 102.5169 | 120.074 | 93.68007 | 113.5614 |
| HY-12053A | Vinorelbine (ditartrate) | chemotherapy/DNA damage | 1.667 | 70.57247 | 10.5508 | 51.5254293 | 3.4992493 | 56.61123 | 32.73821 | 66.75201 | 18.34171 | 26.67309 | 75.91998 | 72.12074 | 33.19523 | 57.70807 |
| HY-B0181 | Altretamine | chemotherapy/DNA damage | 1.667 | 100.1406 | 49.33906 | 100.638968 | 104.75493 | 90.17059 | 122.9014 | 92.90417 | 95.8013 | 100.0291 | 111.7977 | 98.20622 | 95.26233 | 98.41107 |
| HY-13567 | Bendamustine | chemotherapy/DNA damage | 1.667 | 94.79349 | 102.5058 | 104.693769 | 97.300462 | 85.11999 | 119.8227 | 110.2495 | 95.57306 | 104.825 | 114.8322 | 98.62333 | 94.93561 | 105.9048 |
| HY-13780 | Vinblastine (sulfate) | chemotherapy/DNA damage | 1.667 | 69.23962 | 11.04213 | 53.7545929 | 3.1086993 | 53.07085 | 34.56188 | 75.47894 | 19.76053 | 26.91039 | 71.91579 | 68.40437 | 30.87511 | 63.82637 |
| HY-13566A | Belotecan (hydrochloride) | chemotherapy/DNA damage | 1.667 | 45.09498 | 5.782881 | 33.7204044 | 11.563626 | 6.251769 | 3.077694 | 18.58089 | 48.03105 | 1.407536 | 33.42303 | 19.32302 | 19.6189 | 18.49771 |
| HY-13667 | Levoleucovorin (Calcium) | chemotherapy/DNA damage | 1.667 | 100.6621 | 89.82658 | 101.82629 | 96.105824 | 89.35501 | 118.2571 | 100.2655 | 95.8562 | 97.44659 | 87.95021 | 93.94009 | 100.4514 | 113.288 |
| HY-17371 | Oxaliplatin | chemotherapy/DNA damage | 1 | 76.86035 | 78.95204 | 88.3603558 | 92.616872 | 81.49441 | 104.1827 | 84.65311 | 89.24216 | 63.82429 | 89.60463 | 87.14921 | 95.99884 | 92.26263 |
| HY-A0063A | Tipiracil | chemotherapy/DNA damage | 1 | 106.6369 | 93.09367 | 103.754441 | 98.733757 | 95.63534 | 107.3294 | 97.19398 | 96.6141 | 124.6871 | 97.5773 | 117.1673 | 101.7828 | 104.3552 |
| HY-10159 | Nilotinib | TKI | 1.667 | 91.50952 | 94.37062 | 88.6767031 | 81.825244 | 71.59269 | 52.604 | 84.33873 | 105.1441 | 82.1275 | 75.26141 | 84.02642 | 70.66845 | 124.9786 |
| HY-10230 | Midostaurin | TKI | 1.667 | 13.98614 | 41.75325 | 34.9831848 | 19.467296 | 25.66439 | 0.940236 | 38.94295 | 35.21748 | 3.366406 | 34.87518 | 58.54438 | 18.64243 | 49.64211 |
| HY-12297 | Sulfatinib | TKI | 1.667 | 94.56872 | 96.89062 | 87.5786294 | 95.214271 | 86.26536 | 78.44691 | 99.63551 | 98.91019 | 69.07088 | 100.6446 | 70.04505 | 90.87897 | 126.3325 |
| HY-12798A | Netarsudil (dimesylate) | TKI | 1.667 | 142.5298 | 120.0627 | 105.043884 | 64.424452 | 71.74726 | 66.33006 | 112.1563 | 95.55701 | 117.5503 | 134.3521 | 83.01483 | 84.58807 | 143.1702 |
| HY-N0110 | Palmatine (chloride) | TKI | 1.667 | 97.97563 | 107.9722 | 98.6508418 | 99.457514 | 97.46332 | 112.6899 | 117.1872 | 110.3373 | 105.2938 | 111.0204 | 102.8447 | 102.9879 | 138.0972 |
| HY-15164A | Icotinib | TKI | 1.667 | 72.17508 | 92.05835 | 93.4967857 | 95.469629 | 72.60251 | 73.00457 | 104.3825 | 83.32716 | 75.33641 | 67.42683 | 87.97391 | 90.73078 | 124.861 |
| HY-12215 | Lorlatinib | TKI | 1.667 | 90.16866 | 85.41168 | 94.1838236 | 92.975613 | 66.48262 | 60.53958 | 89.46851 | 88.78421 | 82.97678 | 81.5085 | 52.57993 | 102.2 | 119.4101 |
| HY-10261 | Afatinib | TKI | 1.667 | 51.53465 | 41.22288 | 74.2027787 | 66.405247 | 83.82418 | 125.2755 | 63.19104 | 97.78474 | 35.73574 | 51.70199 | 63.47797 | 70.60022 | 73.71985 |
| HY-10209 | Masitinib | TKI | 1.667 | 118.4015 | 112.7773 | 103.107475 | 108.54354 | 93.44343 | 111.3394 | 104.9324 | 109.8786 | 103.0835 | 101.244 | 143.5703 | 95.06169 | 130.9186 |
| HY-15307A | Belumosudil (mesylate) | TKI | 1.667 | 96.68657 | 39.14584 | 110.366513 | 90.725256 | 92.66437 | 84.0509 | 101.5298 | 85.91597 | 105.3802 | 72.64678 | 95.87681 | 92.55229 | 118.7565 |
| HY-15728 | Radotinib | TKI | 1.667 | 125.2534 | 82.19114 | 96.315824 | 97.802502 | 85.83893 | 87.22347 | 95.97014 | 111.0589 | 127.2007 | 100.3505 | 136.6017 | 116.5737 | 140.5728 |
| HY-10997 | Ibrutinib | TKI | 1.667 | 94.61829 | 111.661 | 87.3478979 | 106.01335 | 83.91544 | 82.16755 | 79.2626 | 95.90564 | 45.12907 | 62.11751 | 75.44963 | 85.95762 | 129.0408 |
| HY-18300 | Filgotinib | TKI | 1.667 | 104.7807 | 99.04974 | 96.2940363 | 101.98123 | 98.70215 | 103.2702 | 104.0029 | 99.31698 | 98.25433 | 90.93441 | 104.3642 | 98.71493 | 122.6116 |
| HY-14660 | Dabrafenib | TKI | 1.667 | 47.25487 | 90.65789 | 72.1452254 | 42.503616 | 63.6773 | 36.70298 | 58.04508 | 75.75205 | 36.07171 | 87.69867 | 84.72301 | 135.9263 | 109.6456 |
| HY-15463 | Imatinib | TKI | 1.667 | 124.0283 | 98.74966 | 107.972929 | 111.07387 | 87.70949 | 101.642 | 110.0518 | 106.356 | 112.3792 | 103.2462 | 104.6188 | 95.64807 | 138.1567 |
| HY-10961 | Momelotinib | TKI | 1.667 | 131.734 | 103.416 | 99.4351419 | 69.461505 | 68.01526 | 86.30191 | 91.2887 | 91.48374 | 55.32927 | 110.9809 | 61.95585 | 101.677 | 124.5501 |
| HY-10208 | Pazopanib | TKI | 1.667 | 102.3839 | 97.9524 | 96.3192696 | 90.333235 | 74.75649 | 86.09195 | 76.13851 | 95.78182 | 87.04301 | 90.75837 | 75.2371 | 94.68754 | 123.4713 |
| HY-10201 | Sorafenib | TKI | 1.667 | 101.9048 | 80.81765 | 91.9427604 | 89.731783 | 115.4851 | 134.0738 | 70.69021 | 94.74339 | 129.8712 | 75.90004 | 118.3468 | 103.6783 | 118.8031 |
| HY-101474A | Zanubrutinib | TKI | 1.667 | 87.47849 | 104.378 | 94.2465114 | 110.28128 | 83.02308 | 86.50002 | 98.93293 | 90.83511 | 73.97951 | 61.81066 | 94.05864 | 78.32377 | 122.4301 |
| HY-15666 | Olverembatinib | TKI | 1.667 | 9.604487 | 52.80414 | 33.6083004 | 26.149731 | 48.26566 | 12.88163 | 38.08827 | 69.4542 | 6.340323 | 69.68176 | 29.52002 | 97.05641 | 76.04187 |
| HY-13404 | Capmatinib | TKI | 1.667 | 99.66262 | 93.26882 | 103.268757 | 111.76457 | 46.55081 | 18.84249 | 92.62098 | 66.94552 | 91.43072 | 94.17715 | 110.3017 | 102.9672 | 129.755 |
| HY-13001 | Quizartinib | TKI | 1.667 | 103.1277 | 42.73016 | 100.654789 | 71.211457 | 87.11837 | 57.32545 | 94.77746 | 93.74555 | 89.84027 | 63.60194 | 107.2234 | 86.57344 | 105.2281 |
| HY-16379 | Pacritinib | TKI | 1.667 | 6.572793 | 30.01119 | 60.5692705 | 47.171554 | 46.8777 | 18.75616 | 41.21188 | 83.48494 | 67.92417 | 83.95176 | 89.87627 | 105.8047 | 129.5223 |
| HY-12279C | Umbralisib (hydrochloride) | TKI | 1.667 | 105.6056 | 110.8027 | 105.249792 | 93.956816 | 80.17487 | 74.74541 | 93.53181 | 92.3316 | 109.0059 | 93.59589 | 115.6027 | 100.7846 | 119.4405 |
| HY-13064 | Cobimetinib | TKI | 1.667 | 41.23449 | 11.96567 | 41.8040882 | 4.7203013 | 86.28311 | 118.0752 | 34.04559 | 99.48216 | 4.765911 | 38.41232 | 76.45568 | 34.74418 | 43.00747 |
| HY-13904 | Flumatinib | TKI | 1.667 | 92.35812 | 82.30455 | 96.0024185 | 87.028029 | 95.49548 | 29.21574 | 92.77058 | 98.65551 | 63.43781 | 119.1175 | 84.55286 | 98.97688 | 115.9177 |
| HY-10999 | Trametinib | TKI | 1.667 | 107.5797 | 93.44226 | 117.591243 | 99.884699 | 77.33082 | 81.67502 | 103.6286 | 85.33204 | 113.8062 | 98.74416 | 120.1076 | 108.4316 | 119.4935 |
| HY-15202 | Binimetinib | TKI | 1.667 | 64.23729 | 45.28289 | 72.5111389 | 71.090982 | 78.80438 | 108.9256 | 66.36911 | 90.26281 | 28.43942 | 61.2718 | 117.2667 | 52.5366 | 70.49389 |
| HY-18708 | Erdafitinib | TKI | 1.667 | 53.02111 | 86.37949 | 87.5573936 | 79.633731 | 52.0831 | 23.83656 | 70.79003 | 75.88287 | 38.81047 | 83.79353 | 68.66662 | 84.80396 | 141.2748 |
| HY-10065 | Axitinib | TKI | 1.667 | 109.4739 | 76.73727 | 105.670324 | 51.884059 | 79.8989 | 39.71487 | 106.4973 | 78.69821 | 101.9 | 84.88603 | 82.13081 | 54.89025 | 118.4588 |
| HY-13016 | Cabozantinib | TKI | 1.667 | 92.60806 | 99.17704 | 86.5937409 | 108.12308 | 83.09806 | 97.34301 | 78.98994 | 94.71063 | 58.47882 | 101.6225 | 61.72637 | 90.09918 | 124.3204 |
| HY-109061 | Lazertinib | TKI | 1.667 | 70.22166 | 118.726 | 93.8834183 | 101.47782 | 85.14392 | 13.64801 | 90.10965 | 84.15917 | 86.99095 | 88.7121 | 110.1797 | 100.3527 | 139.1408 |
| HY-13911 | Hydroxyfasudil | TKI | 1.667 | 105.8602 | 99.20679 | 101.917791 | 91.391721 | 90.37937 | 121.8152 | 112.3212 | 98.95951 | 107.9303 | 108.0099 | 114.9529 | 101.7225 | 127.7975 |
| HY-12866 | Larotrectinib | TKI | 1.667 | 109.7734 | 101.8641 | 101.134073 | 103.66083 | 98.42734 | 126.0696 | 110.6512 | 103.6238 | 122.9003 | 99.78846 | 122.0752 | 107.182 | 139.7379 |
| HY-12678 | Entrectinib | TKI | 1.667 | 104.3643 | 98.35602 | 100.666604 | 101.42017 | 95.42509 | 133.3036 | 102.4265 | 96.43483 | 10.20216 | 65.43934 | 15.09505 | 44.35655 | 83.79841 |
| HY-12057 | Vemurafenib | TKI | 1.667 | 109.1447 | 110.2529 | 107.356546 | 92.0902 | 84.10814 | 103.4055 | 98.03082 | 89.96502 | 171.7878 | 102.2389 | 104.9086 | 108.0566 | 131.9945 |
| HY-10255A | Sunitinib | TKI | 1.667 | 52.26968 | 75.03081 | 63.7709785 | 63.112534 | 48.48685 | 21.66737 | 76.91176 | 77.51486 | 25.66514 | 62.50094 | 79.15575 | 101.9519 | 136.0978 |
| HY-I0960 | Uracil | TKI | 1.667 | 107.546 | 110.0767 | 105.775564 | 100.90673 | 102.1881 | 113.5867 | 115.3101 | 102.4514 | 127.6617 | 94.54467 | 124.8311 | 99.77494 | 125.3858 |
| HY-50904 | Nintedanib | TKI | 1.667 | 64.09101 | 98.17642 | 97.9787112 | 90.24679 | 69.56998 | 53.76603 | 109.3001 | 92.23826 | 61.49134 | 109.6963 | 88.41678 | 101.5412 | 127.1235 |
| HY-19912 | Fruquintinib | TKI | 1.667 | 97.06408 | 99.0189 | 102.153117 | 99.191531 | 95.99287 | 83.41126 | 109.3543 | 105.5572 | 107.8923 | 106.0051 | 107.6397 | 92.07339 | 126.1439 |
| HY-100818 | Futibatinib | TKI | 1.667 | 89.52049 | 102.4077 | 103.590121 | 99.118015 | 84.3836 | 61.83507 | 90.15908 | 95.00011 | 70.29494 | 106.3271 | 56.52932 | 69.06472 | 119.8694 |
| HY-10981 | Lenvatinib | TKI | 1.667 | 94.0791 | 109.5401 | 99.2305587 | 101.97071 | 68.33824 | 61.84255 | 109.3885 | 106.3877 | 67.8839 | 99.65514 | 103.0068 | 66.61043 | 134.411 |
| HY-50858 | Ruxolitinib (phosphate) | TKI | 1.667 | 117.6114 | 108.4792 | 104.637749 | 99.812517 | 96.76281 | 97.18129 | 116.719 | 98.59677 | 106.6491 | 104.3621 | 107.702 | 108.3995 | 126.5026 |
| HY-13272 | Dacomitinib | TKI | 1.667 | 39.06302 | 44.16061 | 57.5789445 | 53.467257 | 67.54259 | 71.76696 | 53.21146 | 85.64661 | 21.84517 | 49.57419 | 52.60204 | 80.46728 | 101.5206 |
| HY-B1305 | Chloropyramine hydrochloride | TKI | 1.667 | 99.75002 | 103.6035 | 94.8904363 | 102.08614 | 84.69394 | 114.6435 | 113.1693 | 91.19299 | 105.0139 | 121.2118 | 99.89877 | 100.3372 | 123.1761 |
| HY-10158 | Bosutinib | TKI | 1.667 | 24.60078 | 65.56022 | 46.4499409 | 51.94836 | 43.24383 | 40.20295 | 70.98497 | 74.23671 | 37.09734 | 50.86885 | 39.62181 | 118.6813 | 138.0282 |
| HY-103022 | Repotrectinib | TKI | 1.667 | 21.99903 | 47.86745 | 40.1149636 | 29.263287 | 25.17044 | 18.64395 | 48.37122 | 47.93432 | 30.19236 | 70.00825 | 16.56604 | 84.02179 | 82.52383 |
| HY-50706 | Selumetinib | TKI | 1.667 | 62.59121 | 50.53365 | 64.8070685 | 74.849883 | 82.92369 | 80.72872 | 82.7879 | 87.01834 | 33.97109 | 63.03847 | 113.0711 | 62.06989 | 84.78979 |
| HY-13038 | Fostamatinib Disodium | TKI | 1.667 | 16.03156 | 76.56887 | 61.2489979 | 60.804323 | 56.55393 | 41.47564 | 65.17262 | 72.02644 | 33.23427 | 86.65873 | 25.7948 | 82.13862 | 111.6358 |
| HY-17600 | Acalabrutinib | TKI | 1.667 | 95.04904 | 112.0049 | 94.3141695 | 97.924741 | 92.09763 | 106.638 | 99.09444 | 102.7564 | 115.1216 | 107.7658 | 103.2373 | 97.00514 | 131.3843 |
| HY-16749A | Pexidartinib (hydrochloride) | TKI | 1.667 | 95.3639 | 100.8149 | 102.660514 | 101.33607 | 81.63312 | 135.3064 | 110.4883 | 107.2104 | 103.4251 | 113.8314 | 116.5996 | 104.3112 | 121.7528 |
| HY-19730 | Olmutinib | TKI | 1.667 | 71.0394 | 86.35086 | 83.6413217 | 94.002224 | 73.24943 | 17.15482 | 86.52622 | 73.7659 | 61.83104 | 76.32287 | 95.55267 | 71.11396 | 107.3123 |
| HY-N0281 | Daphnetin | TKI | 1.667 | 103.9739 | 89.18108 | 103.883171 | 103.42843 | 90.73654 | 86.92794 | 99.47885 | 90.96432 | 99.45212 | 103.1819 | 98.56793 | 100.7909 | 121.8349 |
| HY-101561 | Avapritinib | TKI | 1.667 | 60.18878 | 87.77267 | 93.5292287 | 87.963421 | 76.29284 | 76.10917 | 87.86108 | 86.36421 | 74.69918 | 95.50386 | 74.85062 | 90.93748 | 118.9569 |
| HY-50895 | Gefitinib | TKI | 1.667 | 63.59816 | 100.4972 | 80.0736936 | 88.283753 | 45.42164 | 18.65543 | 92.94042 | 62.50018 | 53.50758 | 53.50793 | 72.90744 | 75.13288 | 113.8751 |
| HY-131328 | Pirtobrutinib | TKI | 1.667 | 104.1457 | 97.69777 | 99.1848416 | 104.42842 | 90.16087 | 89.66907 | 108.1536 | 96.5878 | 88.77723 | 104.2051 | 100.9521 | 97.81857 | 123.6022 |
| HY-10342 | Enzastaurin | TKI | 1.667 | 109.9202 | 107.3335 | 100.103839 | 106.47408 | 90.09837 | 120.8746 | 110.2312 | 95.49926 | 107.6549 | 100.9837 | 112.0114 | 94.62834 | 121.3293 |
| HY-16069 | Tucatinib | TKI | 1.667 | 81.36234 | 86.29583 | 87.2982956 | 106.3365 | 52.24792 | 62.0977 | 106.0032 | 58.03193 | 99.74387 | 81.31803 | 84.71708 | 90.68196 | 116.5794 |
| HY-135815 | Mobocertinib | TKI | 1.667 | 66.69241 | 90.6781 | 82.5634195 | 92.455609 | 93.73259 | 131.8525 | 100.7307 | 100.534 | 68.9647 | 59.51877 | 122.3673 | 81.25281 | 123.4794 |
| HY-13011 | Alectinib | TKI | 1.667 | 69.58255 | 79.92641 | 90.3625021 | 102.5042 | 71.43087 | 80.62402 | 105.6757 | 85.20706 | 90.45414 | 87.55278 | 116.0547 | 91.94536 | 117.8222 |
| HY-B0719 | Ingenol Mebutate | TKI | 1.667 | 72.22803 | 68.50571 | 85.4596321 | 56.226375 | 60.50855 | 47.557 | 92.01853 | 84.0851 | 141.9307 | 82.90592 | 124.3998 | 84.05642 | 101.5172 |
| HY-10409 | Fedratinib | TKI | 1.667 | 6.894425 | 46.4167 | 44.0127662 | 33.076745 | 32.48 | 8.126913 | 49.03965 | 45.81514 | 8.912292 | 55.5879 | 25.70394 | 67.12657 | 83.57307 |
| HY-10260 | Vandetanib | TKI | 1.667 | 41.93724 | 89.61211 | 65.271937 | 86.547931 | 57.04079 | 27.47697 | 62.57592 | 88.28483 | 34.53349 | 49.59359 | 46.22122 | 83.80601 | 115.165 |
| HY-10999A | Trametinib (DMSO solvate) | TKI | 1.667 | 45.38501 | 13.35173 | 48.6241208 | 4.1552295 | 84.68796 | 70.8927 | 38.96492 | 81.25767 | 4.763459 | 37.66613 | 88.12858 | 28.66216 | 38.92293 |
| HY-15431 | Capivasertib | TKI | 1.667 | 78.48514 | 77.54431 | 99.2207614 | 101.43922 | 71.01581 | 71.00091 | 94.34227 | 88.7625 | 95.1291 | 100.856 | 77.2423 | 80.47554 | 112.1879 |
| HY-109099 | Pemigatinib | TKI | 1.667 | 34.84055 | 89.01447 | 55.3085611 | 70.828791 | 49.06271 | 15.3629 | 69.57104 | 52.85404 | 26.66963 | 72.27941 | 87.84989 | 84.5036 | 110.8852 |
| HY-50878 | Crizotinib | TKI | 1.667 | 97.9139 | 37.79849 | 67.7129618 | 36.784219 | 50.46552 | 34.02747 | 71.56528 | 69.15993 | 23.34275 | 90.95946 | 20.83046 | 56.55432 | 68.89005 |
| HY-129390 | Orelabrutinib | TKI | 1.667 | 91.79664 | 97.86673 | 97.7499944 | 98.622843 | 87.67684 | 100.5393 | 87.70468 | 95.82958 | 136.0282 | 104.3319 | 108.2672 | 97.0277 | 103.9362 |
| HY-13026 | Idelalisib | TKI | 1.667 | 55.87738 | 92.81312 | 87.7619153 | 100.37461 | 52.03461 | 46.80693 | 66.74101 | 63.7854 | 89.21212 | 89.88112 | 82.1445 | 86.31019 | 101.0423 |
| HY-112301 | Pralsetinib | TKI | 1.667 | 12.50074 | 73.25467 | 57.8498061 | 75.414878 | 37.76938 | 19.68904 | 63.56751 | 73.98778 | 23.87134 | 64.27479 | 33.35585 | 64.24062 | 91.00816 |
| HY-15244 | Alpelisib | TKI | 1.667 | 67.63277 | 64.17956 | 88.3415034 | 88.370039 | 91.7782 | 110.2574 | 59.36756 | 101.01 | 68.47887 | 90.4909 | 62.12572 | 84.47076 | 86.36332 |
| HY-32721 | Neratinib | TKI | 1.667 | 30.49301 | 44.42399 | 52.5036216 | 45.128768 | 99.11853 | 125.6144 | 33.84662 | 93.66172 | 30.75872 | 39.24995 | 57.48484 | 85.25892 | 78.0045 |
| HY-112823 | Almonertinib | TKI | 1.667 | 68.23569 | 90.98259 | 82.587694 | 91.828358 | 98.12757 | 128.5649 | 68.42091 | 94.01678 | 65.19125 | 56.12891 | 57.75495 | 78.17687 | 94.01619 |
| HY-N0060 | Ferulic acid | TKI | 1.667 | 97.5897 | 97.2529 | 100.801884 | 113.18708 | 86.11413 | 94.80903 | 95.70445 | 95.37837 | 107.5026 | 108.898 | 105.7893 | 111.5338 | 107.8036 |
| HY-50898 | Lapatinib | TKI | 1.667 | 69.40313 | 106.0044 | 87.0556952 | 83.440896 | 78.22979 | 85.19904 | 90.77076 | 89.76598 | 66.88832 | 67.55204 | 86.07735 | 92.11949 | 104.0852 |
| HY-50896 | Erlotinib | TKI | 1.667 | 49.79204 | 82.67181 | 81.8846708 | 82.039955 | 75.16672 | 75.79504 | 77.85248 | 78.62729 | 56.72097 | 48.33629 | 77.24881 | 74.94764 | 99.21163 |
| HY-17044 | Duvelisib | TKI | 1.667 | 50.29439 | 78.32345 | 83.7016579 | 84.191642 | 79.89848 | 92.40916 | 65.96285 | 83.74718 | 54.8811 | 94.14304 | 74.7681 | 79.96076 | 98.4172 |
| HY-13311 | Infigratinib | TKI | 1.667 | 59.14143 | 107.1761 | 86.9666325 | 76.835974 | 63.01669 | 33.367 | 80.13935 | 83.10855 | 13.15413 | 69.85802 | 53.85024 | 68.62928 | 97.92529 |
| HY-10340 | Tirbanibulin | TKI | 1.667 | 79.5823 | 11.35253 | 59.9459141 | 2.773051 | 53.54929 | 70.04082 | 61.75871 | 20.97295 | 44.96022 | 59.43021 | 56.73947 | 32.23546 | 67.70469 |
| HY-112306 | Ripretinib | TKI | 1.667 | 18.05225 | 53.58139 | 58.1051026 | 37.629378 | 62.93279 | 22.51696 | 53.73778 | 79.31236 | 16.21162 | 71.98604 | 35.41786 | 83.39347 | 65.76689 |
| HY-10331 | Regorafenib | TKI | 1.667 | 83.41848 | 72.87039 | 91.8960934 | 87.945017 | 50.22274 | 36.17726 | 83.92328 | 20.13547 | 98.33975 | 81.35263 | 90.11046 | 85.43211 | 90.99221 |
| HY-15959 | Savolitinib | TKI | 1.667 | 98.13682 | 81.92534 | 99.2367971 | 102.79528 | 64.84702 | 79.38471 | 105.8878 | 97.13255 | 101.0073 | 105.0557 | 89.93667 | 105.8834 | 109.9777 |
| HY-15605 | Encorafenib | TKI | 1.667 | 33.30622 | 64.58548 | 61.8527734 | 46.191464 | 80.63554 | 55.44171 | 54.51836 | 95.75893 | 18.60174 | 57.8904 | 56.10561 | 107.348 | 77.75646 |
| HY-101820 | Simotinib | TKI | 1.667 | 60.16709 | 92.5699 | 83.2209846 | 88.243397 | 94.31058 | 129.1739 | 82.6498 | 103.0059 | 75.67893 | 66.05934 | 86.42807 | 89.67853 | 96.28027 |
| HY-103714A | Ensartinib (dihydrochloride) | TKI | 1.667 | 79.13643 | 31.57418 | 82.816241 | 45.546942 | 53.95275 | 38.7185 | 81.96102 | 71.45019 | 53.37149 | 73.20504 | 26.15007 | 70.27014 | 66.95523 |
| HY-15772 | Osimertinib | TKI | 1.667 | 61.97831 | 87.51994 | 81.4923947 | 83.022536 | 95.26782 | 50.54889 | 70.63781 | 102.4151 | 44.50063 | 46.3356 | 55.36086 | 61.58782 | 92.56106 |
| HY-10977 | Tivozanib | TKI | 1.667 | 96.20573 | 103.6918 | 90.0319064 | 92.192977 | 84.0463 | 82.46891 | 101.8075 | 102.5582 | 92.18884 | 77.51274 | 53.72837 | 87.95222 | 102.2353 |
| HY-114370 | Selpercatinib | TKI | 1.667 | 84.61083 | 91.88125 | 85.7000969 | 102.62774 | 82.10804 | 66.90824 | 90.70037 | 88.18403 | 60.61636 | 87.26752 | 68.86863 | 83.30639 | 99.77267 |
| HY-12047 | Ponatinib | TKI | 1.667 | 0.391339 | 26.89763 | 16.2803044 | 3.4478758 | 23.9225 | 0.737139 | 31.63798 | 40.791 | 2.617904 | 38.28781 | 11.35547 | 53.80913 | 28.84858 |
| HY-104010 | Asciminib | TKI | 1.667 | 99.08512 | 97.84883 | 103.011693 | 97.655956 | 96.5365 | 82.22474 | 84.77701 | 101.4019 | 107.3831 | 99.6015 | 110.4473 | 90.79504 | 98.85454 |
| HY-10341 | Fasudil (Hydrochloride) | TKI | 1.667 | 103.3286 | 103.1024 | 113.405103 | 98.179109 | 94.78249 | 121.2899 | 106.0015 | 97.15146 | 100.338 | 91.10682 | 99.33976 | 89.44574 | 109.4656 |
| HY-10181 | Dasatinib | TKI | 1.667 | 33.47794 | 77.09329 | 41.5402441 | 32.191822 | 46.77084 | 45.8967 | 34.26823 | 67.60268 | 39.75302 | 58.38158 | 42.30053 | 109.6872 | 74.33157 |
| HY-15346A | Copanlisib (dihydrochloride) | TKI | 1.667 | 18.80554 | 14.906 | 57.0249379 | 17.210833 | 58.60399 | 33.74121 | 31.29915 | 75.65042 | 1.476786 | 37.49767 | 26.63611 | 8.037985 | 46.99678 |
| HY-12432 | Gilteritinib | TKI | 1 | 12.86106 | 22.70272 | 24.2124811 | 21.3289 | 29.23649 | 13.88053 | 38.43294 | 34.65268 | 5.852241 | 55.17348 | 14.59661 | 64.13834 | 59.58155 |
| HY-12857 | Brigatinib | TKI | 1 | 44.90962 | 26.30078 | 58.3837471 | 46.523917 | 20.59777 | 29.32429 | 48.27467 | 37.6122 | 8.381013 | 50.00572 | 31.14629 | 75.51269 | 58.33879 |
| HY-10330 | Toceranib | TKI | 1 | 62.28072 | 77.87463 | 81.3153906 | 85.706649 | 64.06481 | 80.73869 | 87.9902 | 86.84963 | 45.58961 | 91.73947 | 75.65321 | 97.55738 | 104.0495 |
| HY-N1373 | Sophoridine | Apoptosis | 1.667 | 96.73376 | 111.8159 | 96.026315 | 104.32562 | 85.43543 | 118.0439 | 103.2799 | 99.08276 | 92.56356 | 107.9118 | 135.7849 | 107.4556 | 133.604 |
| HY-107324 | ?-Elemene | Apoptosis | 1.667 | 105.87 | 92.9439 | 100.085762 | 112.37822 | 89.20091 | 124.2412 | 110.9755 | 100.2143 | 112.7417 | 106.4854 | 128.0379 | 110.3261 | 129.6285 |
| HY-50935 | Troglitazone | Apoptosis | 1.667 | 107.8102 | 128.1512 | 101.677494 | 88.619093 | 109.3929 | 181.1339 | 132.5408 | 103.3304 | 114.4342 | 104.877 | 136.9419 | 91.04443 | 122.2582 |
| HY-19696A | Tauroursodeoxycholate (sodium) | Apoptosis | 1.667 | 101.5046 | 109.4707 | 105.453468 | 108.00061 | 98.36692 | 114.743 | 92.6048 | 102.2121 | 100.7505 | 98.05343 | 119.8513 | 104.6856 | 124.1626 |
| HY-13680 | Meisoindigo | Apoptosis | 1.667 | 89.86368 | 81.1175 | 103.058567 | 100.83549 | 88.83061 | 100.8084 | 89.07397 | 81.78546 | 81.2091 | 97.55066 | 89.82017 | 92.67263 | 105.2134 |
| HY-N0124 | Dioscin | Apoptosis | 1.667 | 77.91303 | 35.88546 | 74.7112203 | 44.859069 | 58.03407 | 67.51954 | 54.97787 | 66.03895 | 79.51542 | 63.80434 | 74.80165 | 95.44231 | 89.38906 |
| HY-15531 | Venetoclax | Apoptosis | 1.667 | 136.7449 | 102.9211 | 131.989789 | 87.318507 | 100.3523 | 130.7691 | 96.65449 | 102.2291 | 119.1337 | 108.6938 | 141.9906 | 107.7265 | 140.663 |
| HY-N0714A | Berbamine (dihydrochloride) | Apoptosis | 1.667 | 71.46777 | 87.2213 | 97.6006563 | 81.684878 | 81.48636 | 23.57849 | 88.9354 | 85.60711 | 60.44677 | 95.98328 | 84.71644 | 96.91933 | 100.2663 |
| HY-10452 | Ixazomib citrate | Apoptosis | 1.667 | 17.69287 | 1.581326 | 48.1996633 | 7.4032081 | 1.993685 | 0.13895 | 7.094185 | 2.58669 | 2.452694 | 84.73277 | 10.2713 | 8.166882 | 27.45478 |
| HY-B0313 | Hydroxyurea | Apoptosis | 1.667 | 99.52764 | 91.7861 | 101.210396 | 108.92725 | 91.95284 | 103.5449 | 95.45837 | 99.23417 | 105.6876 | 104.0169 | 114.9994 | 101.911 | 109.8097 |
| HY-13407 | Gossypol | Apoptosis | 1.667 | 109.7251 | 104.5104 | 100.451447 | 98.868287 | 93.6712 | 99.50871 | 102.658 | 98.69983 | 114.6894 | 111.7405 | 117.7688 | 101.607 | 95.46968 |
| HY-14944 | Homoharringtonine | Apoptosis | 1.667 | 5.026158 | 1.863217 | 42.7705668 | 0.2782564 | 7.214852 | 0.053694 | 20.87172 | 4.766564 | 5.966094 | 37.30916 | 21.35177 | 2.209499 | 19.40126 |
| HY-10219 | Rapamycin | autophagy | 1.667 | 43.95096 | 46.16084 | 88.189061 | 60.235043 | 53.39255 | 96.63645 | 63.98083 | 60.75097 | 53.46766 | 59.8745 | 93.25998 | 40.88214 | 98.74809 |
| HY-10218 | Everolimus | autophagy | 1.667 | 46.89714 | 51.41291 | 90.6885967 | 63.714761 | 49.09958 | 72.98863 | 68.13255 | 60.84805 | 66.22215 | 67.99231 | 94.54059 | 43.98098 | 94.87027 |
| HY-B2098 | Lucanthone | autophagy | 1.667 | 91.71218 | 96.49746 | 100.96789 | 108.2434 | 99.13223 | 55.40246 | 102.5898 | 91.51997 | 115.5884 | 102.3501 | 102.5499 | 105.2536 | 129.0176 |
| HY-A0061 | Trifluridine | autophagy | 1.667 | 96.28461 | 87.96333 | 96.7420791 | 57.965007 | 71.66142 | 109.1897 | 86.29715 | 86.10803 | 108.9106 | 104.5355 | 126.1028 | 79.72635 | 104.595 |
| HY-50856 | Ruxolitinib | autophagy | 1.667 | 117.6989 | 100.8095 | 107.341876 | 99.352006 | 94.16753 | 90.24377 | 97.53533 | 95.44755 | 103.1989 | 101.297 | 94.8183 | 118.9961 | 108.8224 |
| HY-50767C | Palbociclib (hydrochloride) | cell cycle | 1.667 | 66.90098 | 83.79783 | 77.7313953 | 59.796572 | 48.33418 | 96.10471 | 77.85734 | 84.94319 | 70.09406 | 71.12419 | 110.5731 | 68.74162 | 110.8065 |
| HY-16209 | Forodesine (hydrochloride) | cell cycle/ DNA damage | 1.667 | 82.94353 | 101.7293 | 97.711721 | 92.339954 | 90.25157 | 122.0059 | 68.22796 | 93.6847 | 95.98339 | 93.94403 | 100.3527 | 102.6814 | 124.8277 |
| HY-13674 | Maytansine | cell cycle/ DNA damage | 1.667 | 72.62596 | 7.552098 | 33.6054524 | 2.830429 | 46.03259 | 27.79366 | 72.70158 | 21.66893 | 41.69337 | 78.07037 | 74.79377 | 40.39697 | 77.1789 |
| HY-129071 | Vindesine (sulfate) | cell cycle/ DNA damage | 1.667 | 69.76972 | 10.12501 | 43.7924239 | 2.3765023 | 49.53315 | 28.90417 | 80.87377 | 19.83759 | 31.39451 | 82.23169 | 81.60234 | 38.23499 | 76.14564 |
| HY-16210 | Forodesine | cell cycle/ DNA damage | 1.667 | 94.53236 | 88.58768 | 103.980372 | 107.07047 | 91.91183 | 112.85 | 69.94668 | 94.94901 | 112.5365 | 92.19533 | 105.0659 | 110.2249 | 137.2196 |
| HY-10492 | Dinaciclib | cell cycle | 1.667 | 2.288544 | 3.243468 | 59.555169 | 6.1392332 | 11.54985 | 0.737737 | 17.3288 | 10.11552 | 3.330885 | 61.56829 | 15.13079 | 3.104489 | 41.03373 |
| HY-15777 | Ribociclib | cell cycle | 1.667 | 98.36777 | 96.80588 | 90.4548904 | 90.543216 | 81.78265 | 120.4484 | 84.34933 | 98.70087 | 104.1773 | 83.09963 | 99.49548 | 76.08237 | 119.6748 |
| HY-10617A | Rucaparib | DNA damage | 1.667 | 96.81952 | 101.3974 | 109.181886 | 91.223657 | 91.82357 | 110.7525 | 110.3556 | 98.34731 | 98.1355 | 134.4983 | 115.0144 | 84.49952 | 121.5294 |
| HY-17469 | Gimeracil | DNA damage | 1.667 | 96.97116 | 101.8005 | 98.8293186 | 101.88568 | 89.33807 | 106.3913 | 105.6726 | 104.195 | 101.0567 | 93.45214 | 111.1328 | 109.6274 | 131.284 |
| HY-10162 | Olaparib | DNA damage | 1.667 | 91.89178 | 75.28643 | 94.7734613 | 95.835782 | 73.65879 | 83.80977 | 115.2544 | 98.62448 | 121.2838 | 102.1358 | 91.57438 | 91.46144 | 120.1789 |
| HY-10619 | Niraparib | DNA damage | 1.667 | 99.51472 | 79.04566 | 95.660751 | 94.234635 | 77.81479 | 70.448 | 101.6976 | 98.83969 | 92.59127 | 107.3343 | 100.6833 | 79.72536 | 121.4234 |
| HY-104044 | Pamiparib | DNA damage | 1.667 | 89.13202 | 105.338 | 93.1231494 | 94.309247 | 74.21365 | 113.935 | 87.3064 | 97.27216 | 103.4764 | 112.2425 | 91.85136 | 88.30912 | 101.2876 |
| HY-16106 | Talazoparib | DNA damage | 1.667 | 72.33957 | 59.07347 | 79.6702449 | 35.607216 | 48.0212 | 66.09239 | 83.93316 | 82.64224 | 60.37437 | 94.66465 | 90.46172 | 48.06228 | 90.27956 |
| HY-12137 | Volasertib | cell cycle | 1.667 | 40.73558 | 3.491642 | 58.6396484 | 4.5793433 | 31.47718 | 11.48308 | 44.39892 | 26.55167 | 28.41714 | 61.5537 | 40.75007 | 24.45691 | 51.66615 |
| HY-12009 | Pazopanib (Hydrochloride) | DNA damage | 1.667 | 95.32178 | 87.5772 | 85.6425073 | 85.072883 | 77.97339 | 82.59268 | 86.47675 | 94.69413 | 93.0803 | 92.65897 | 71.92883 | 97.31894 | 104.4381 |
| HY-114778 | Fluzoparib | DNA damage | 1.667 | 91.5692 | 83.99955 | 95.8331459 | 90.265211 | 85.35091 | 97.54914 | 90.81898 | 91.76674 | 125.4176 | 98.8145 | 81.34131 | 94.77877 | 104.4715 |
| HY-16297A | Abemaciclib | cell cycle | 1 | 41.72329 | 35.03999 | 58.900867 | 28.188461 | 39.54702 | 39.33672 | 49.81925 | 63.64053 | 63.80342 | 54.23742 | 30.88778 | 41.04205 | 62.04606 |
| HY-109108 | Valemetostat | epigenetics | 10 | 97.52058 | 98.30793 | 99.9310392 | 97.799169 | 88.264 | 74.36134 | 100.2148 | 104.681 |  | 88.0321 | 106.7421 | 87.40124 | 121.4472 |
| HY-18690 | Enasidenib | epigenetics | 10 | 99.64945 | 121.3812 | 109.851184 | 97.373178 | 87.03939 | 47.79051 | 83.45123 | 108.7139 | 119.7859 | 92.60512 | 99.03907 | 95.05233 | 112.9282 |
| HY-13322 | Pracinostat | epigenetics | 10 | 6.459492 | 1.053509 | 19.8765196 | 3.6562122 | 5.275572 | 2.212308 | 9.951686 | 20.58063 | 4.143845 | 33.96398 | 27.48149 | 1.378842 | 28.08045 |
| HY-12164 | Mocetinostat | epigenetics | 10 | 23.2956 | 12.11739 | 41.9396273 | 6.9338462 | 32.58519 | 2.285217 | 16.95106 | 22.19095 | 5.30961 | 47.22581 | 24.47091 | 2.340936 | 32.50146 |
| HY-10225 | Belinostat | epigenetics | 10 | 9.53706 | 0.526562 | 24.6540936 | 1.9729438 | 5.480687 | 1.393807 | 13.2376 | 21.9629 | 2.539046 | 34.84376 | 34.13697 | 1.478437 | 30.369 |
| HY-10224 | Panobinostat | epigenetics | 10 | 2.874677 | 1.428971 | 11.6663152 | 2.4964138 | 5.339658 | 0.396412 | 3.199754 | 4.850695 | 1.258789 | 23.72607 | 8.409524 | 1.119648 | 19.97872 |
| HY-13426 | Roxadustat | epigenetics | 10 | 94.37591 | 116.5174 | 92.5443515 | 74.596458 | 90.09689 | 95.67296 | 91.39977 | 96.72866 | 104.8318 | 90.92791 | 118.1255 | 97.57641 | 92.60964 |
| HY-109015 | Tucidinostat | epigenetics | 10 | 43.78553 | 30.10522 | 74.8200115 | 40.419877 | 62.8446 | 20.24054 | 52.05606 | 44.05564 | 26.70935 | 46.75176 | 50.31115 | 15.17513 | 60.17875 |
| HY-B1625 | Deferoxamine | hypoxia | 1.667 | 77.51728 | 91.96613 | 77.9192118 | 64.601245 | 59.57781 | 66.03402 | 92.10518 | 81.97421 | 71.18637 | 82.80609 | 75.03721 | 94.36061 | 104.1852 |
| HY-N0523 | Gallic acid | hypoxia | 1.667 | 96.52075 | 108.8817 | 97.1584908 | 109.6856 | 97.94755 | 147.5584 | 114.1014 | 98.49403 | 101.3535 | 97.86865 | 125.7054 | 105.1516 | 124.9307 |
| HY-N0733 | Glucosamine (hydrochloride) | hypoxia | 1.667 | 109.8929 | 106.0712 | 104.981533 | 103.90862 | 89.84487 | 119.3144 | 112.5114 | 103.5711 | 106.6808 | 95.48723 | 98.48067 | 107.8653 | 133.3085 |
| HY-125840 | Belzutifan | hypoxia | 1.667 | 110.6445 | 100.2878 | 79.7478529 | 100.71386 | 99.76678 | 158.7033 | 102.7518 | 95.05948 | 117.3212 | 121.9257 | 110.5797 | 108.7123 | 135.5617 |
| HY-17363 | Dimethyl fumarate | hypoxia | 10 | 60.20111 | 44.14918 | 83.2297675 | 81.253868 | 68.92869 | 109.7981 | 69.76014 | 62.49708 | 42.79431 | 94.75364 | 68.60489 | 85.42991 | 94.8226 |
| HY-18258 | Berberine (chloride) | hypoxia | 1.667 | 71.36023 | 69.72637 | 89.0059536 | 87.955432 | 52.7232 | 101.8642 | 43.99752 | 79.24049 | 80.76461 | 85.76766 | 101.1395 | 90.41021 | 104.6885 |
| HY-N0193 | Artesunate | hypoxia | 1.667 | 98.25135 | 56.77944 | 76.3323817 | 95.266939 | 87.589 | 107.6694 | 86.58273 | 85.77615 | 111.2028 | 75.16967 | 110.429 | 76.36076 | 87.4403 |
| HY-B1640 | Ethacrynic acid | hypoxia | 10 | 57.32725 | 52.70838 | 72.4490427 | 69.291817 | 63.6137 | 97.99575 | 46.38927 | 50.3497 | 61.0857 | 82.5846 | 62.79977 | 82.42741 | 80.66161 |
| HY-14649 | Retinoic acid | hypoxia | 1.667 | 92.19345 | 98.57953 | 91.4184385 | 101.35978 | 99.62067 | 98.59416 | 99.18388 | 95.91681 | 126.2329 | 112.4951 | 67.536 | 99.17431 | 99.35807 |
| HY-N0018 | Daidzin | metabolism | 1.667 | 102.4921 | 113.4211 | 94.1601931 | 102.10595 | 87.13735 | 117.981 | 86.45741 | 108.6443 | 110.2809 | 95.82177 | 100.7305 | 105.8472 | 121.2636 |
| HY-B0627 | Metformin | metabolism | 1.667 | 108.6683 | 112.113 | 100.732777 | 104.89574 | 94.96736 | 112.2009 | 113.1645 | 99.72649 | 106.4613 | 105.5492 | 124.9147 | 107.4315 | 123.4875 |
| HY-B0364A | Dyclonine (hydrochloride) | metabolism | 10 | 55.19029 | 48.184 | 68.0885275 | 70.650589 | 59.48676 | 56.26279 | 28.75421 | 53.96813 | 37.59205 | 95.5537 | 71.65154 | 78.9205 | 100.8291 |
| HY-16500 | Tolrestat | metabolism | 1.667 | 105.9688 | 104.1065 | 103.385088 | 106.64821 | 96.31394 | 127.5857 | 101.2046 | 102.969 | 95.20438 | 102.2001 | 121.4253 | 105.0197 | 123.3583 |
| HY-76847 | Chenodeoxycholic Acid | metabolism | 1.667 | 116.3115 | 99.88827 | 104.759299 | 118.5311 | 90.20865 | 113.6532 | 116.2116 | 99.69144 | 111.0613 | 99.99176 | 96.47471 | 105.165 | 145.2158 |
| HY-B0589 | Atorvastatin | metabolism | 1.667 | 106.0931 | 118.9415 | 107.184499 | 109.69621 | 79.30654 | 89.54941 | 81.47809 | 102.3171 | 90.79645 | 97.15679 | 128.5017 | 98.26377 | 124.4146 |
| HY-B0144A | Pitavastatin | metabolism | 1.667 | 102.8128 | 88.09312 | 111.611547 | 103.9088 | 37.68962 | 77.47123 | 15.67212 | 95.06897 | 31.35338 | 84.02242 | 95.10844 | 96.27102 | 96.87512 |
| HY-B0240 | Disulfiram | metabolism | 1.667 | 107.74 | 5.81235 | 109.9772 | 3.1705433 | 9.796322 | 0.016297 | 23.04488 | 20.88133 | 37.69184 | 61.99298 | 98.97909 | 69.02686 | 88.85437 |
| HY-N0198 | Nordihydroguaiaretic acid | metabolism | 1.667 | 99.9007 | 114.2926 | 105.153027 | 106.08286 | 94.22846 | 172.8022 | 110.6348 | 100.5188 | 124.2877 | 119.422 | 133.3532 | 103.7241 | 125.526 |
| HY-17504 | Rosuvastatin (Calcium) | metabolism | 1.667 | 101.3883 | 107.6708 | 107.99259 | 101.80969 | 87.425 | 98.66042 | 109.1885 | 102.0973 | 114.105 | 91.96461 | 111.8703 | 107.9762 | 130.1566 |
| HY-N0164 | Matrine | metabolism | 1.667 | 101.6071 | 103.6105 | 100.061116 | 106.18168 | 95.2686 | 124.3927 | 107.3184 | 99.75087 | 117.1758 | 113.1905 | 114.4103 | 105.0216 | 129.9135 |
| HY-B0218 | Orlistat | metabolism | 1.667 | 106.4041 | 91.79178 | 100.373666 | 100.9569 | 83.33207 | 81.9249 | 94.95777 | 102.7384 | 121.4677 | 100.4067 | 117.4261 | 104.6858 | 100.0433 |
| HY-114226 | Olutasidenib | metabolism | 1.667 | 98.09245 | 107.6635 | 95.5692815 | 99.303698 | 89.63658 | 103.0251 | 100.6947 | 91.63549 | 110.3357 | 95.9627 | 94.34595 | 94.31961 | 104.1199 |
| HY-18767 | Ivosidenib | metabolism | 1.667 | 95.95899 | 95.99833 | 100.753581 | 104.57554 | 93.91726 | 109.6437 | 96.46832 | 95.60274 | 88.9691 | 89.18566 | 100.5157 | 93.59962 | 93.28349 |
| HY-B0808 | Oxaprozin | NFKB | 1.667 | 96.30831 | 98.58398 | 100.88774 | 101.22639 | 86.83285 | 125.4202 | 101.147 | 99.01238 | 105.1686 | 121.9389 | 105.5149 | 105.3179 | 128.6219 |
| HY-B0167A | Sodium Salicylate | NFKB | 1.667 | 98.99865 | 98.60532 | 94.7724738 | 102.51475 | 84.91782 | 141.3708 | 99.03068 | 94.50767 | 108.9061 | 101.1396 | 118.3232 | 111.507 | 128.4924 |
| HY-15122 | Sinomenine | NFKB | 1.667 | 98.58709 | 105.0781 | 91.9616971 | 99.219362 | 91.93631 | 133.1862 | 100.1007 | 98.60015 | 98.04262 | 98.29741 | 127.7504 | 92.06324 | 130.9368 |
| HY-32735 | Triptolide | NFKB | 1.667 | 1.612756 | 2.743897 | 55.7500132 | 2.3695362 | 8.548107 | 0.344464 | 9.945918 | 9.034547 | 3.395544 | 52.82224 | 8.392987 | 4.17032 | 33.52641 |
| HY-N0510 | Aristolochic acid A | NFKB | 1.667 | 98.24131 | 95.49953 | 102.458143 | 102.24601 | 95.30871 | 117.038 | 101.1023 | 94.17816 | 104.3698 | 90.29866 | 106.3212 | 97.17046 | 109.2946 |
| HY-N0052A | Sanguinarine (chloride) | NFKB | 1 | 61.35036 | 8.757754 | 58.9470356 | 52.042598 | 22.63991 | 10.92028 | 30.13049 | 64.94288 | 40.23441 | 81.30543 | 81.05331 | 71.62982 | 67.27667 |
| HY-114277 | Sotorasib | other | 1.667 | 44.57408 | 25.32769 | 39.94639 | 79.406791 | 115.157 | 124.8118 | 98.43225 | 107.5083 | 135.7042 | 106.6926 | 148.8226 | 101.9049 | 165.0325 |
| HY-17536 | Selinexor | other | 1.667 | 79.46664 | 22.01712 | 59.4943296 | 26.143456 | 16.32587 | 16.49818 | 40.53395 | 27.73577 | 36.49963 | 71.63043 | 59.22058 | 18.75558 | 62.52679 |
| HY-B0667 | Balsalazide | other | 1.667 | 105.0436 | 110.6033 | 108.707749 | 110.8517 | 96.47556 | 117.3722 | 94.18596 | 101.8633 | 105.511 | 105.9188 | 128.3142 | 108.8848 | 129.5773 |
| HY-B0146 | Verteporfin | other | 1.667 | 120.2546 | 132.6404 | 124.898364 | 107.66454 | 102.0128 | 130.9006 | 104.8467 | 105.4934 | 116.7205 | 112.0765 | 141.3743 | 106.1726 | 134.7043 |
| HY-15373 | Fenretinide | other | 1.667 | 125.358 | 103.4478 | 84.1569842 | 98.34658 | 118.267 | 38.05595 | 103.4424 | 97.56254 | 132.1565 | 101.9237 | 122.0398 | 106.1222 | 131.0406 |
| HY-N0196 | Baicalein | other | 1.667 | 108.2664 | 120.9669 | 107.491414 | 117.09564 | 107.2205 | 197.5965 | 117.7337 | 99.19376 | 123.9986 | 117.7756 | 138.1297 | 101.9982 | 124.5598 |
| HY-N0156 | Oleanolic Acid | other | 1.667 | 106.5654 | 115.4001 | 109.205448 | 105.0893 | 90.60058 | 113.0368 | 114.4363 | 104.6869 | 101.8398 | 101.9482 | 121.4538 | 100.3044 | 132.2208 |
| HY-130149 | Adagrasib | other | 1.667 | 31.86412 | 15.17463 | 20.2131128 | 48.210405 | 86.78297 | 27.56432 | 77.23237 | 83.63118 | 31.55733 | 118.2191 | 99.88631 | 74.65323 | 90.828 |
| HY-15136 | Lonafarnib | other | 1.667 | 91.57993 | 73.28943 | 94.1866998 | 47.887338 | 71.39082 | 92.53248 | 78.93789 | 77.87648 | 122.0478 | 105.2495 | 75.04763 | 70.73897 | 104.1645 |
| HY-10453 | Ixazomib | proteasome | 1.667 | 18.85367 | 1.684829 | 49.9202658 | 9.1035929 | 2.63324 | 0.204478 | 5.918686 | 2.755519 | 2.973153 | 75.3747 | 11.25568 | 8.318411 | 28.40285 |
| HY-10227 | Bortezomib | proteasome | 1.667 | 4.251423 | 0.245071 | 23.6420322 | 0.4423486 | 1.40083 | 0.07634 | 1.245727 | 2.062117 | 1.519721 | 47.30399 | 1.095841 | 3.703868 | 10.88695 |
| HY-B0380A | Trimebutine (maleate) | stemness | 1.667 | 99.94992 | 104.2836 | 96.3244892 | 96.558703 | 82.34293 | 78.5998 | 75.91373 | 103.057 | 98.67134 | 97.52565 | 98.75693 | 102.5532 | 136.4854 |
| HY-13748 | Silybin A | stemness | 1.667 | 100.4732 | 92.7081 | 97.1452143 | 93.678065 | 80.96714 | 110.8491 | 92.36849 | 94.56223 | 101.7401 | 99.90704 | 120.2587 | 111.5694 | 129.8614 |
| HY-16582A | Sonidegib | stemness/ WNT | 1.667 | 102.8927 | 90.9416 | 96.2528055 | 103.03779 | 86.13536 | 84.79741 | 107.0448 | 96.59631 | 130.7509 | 104.2532 | 131.885 | 104.2795 | 118.0114 |
| HY-16582 | Sonidegib (diphosphate) | stemness/ WNT | 1.667 | 106.051 | 103.1582 | 102.986583 | 104.87768 | 91.82303 | 90.85868 | 102.0323 | 100.724 | 102.1746 | 93.87271 | 108.3493 | 100.6683 | 127.191 |
| HY-16391 | Glasdegib | stemness/ WNT | 1.667 | 105.7691 | 118.2966 | 106.237209 | 117.5256 | 98.23989 | 133.4745 | 95.56652 | 101.6137 | 101.7807 | 101.7258 | 130.0463 | 109.5253 | 135.3115 |
