# Supplementary material for: Targeting KRAS Codon 13 Mutations Using Direct Combination Approaches in Non–Small Cell Lung Cancer
Source: Cancer Discov. 2026 Jun 22;16(11):2274–97. doi: 10.1158/2159-8290.CD-25-0884 (PMC13725345; doi:10.1158/2159-8290.CD-25-0884)
Supplement: Supplementary file 4 [file cd-25-0884_supplementary_file_3_suppsd3.docx]

| **patient.ID** | **KRASmut** | **KRAS.codon** | **KRAS.mutation** | **KEAP1.mutation** | **STK11.mutation** | **NF1.mutation** | **RAG** | **RI** | **B.cells** | **CD45** | **Cytotoxic.cells** | **DC** | **Exhausted.CD8** | **Macrophages** | **Mast.cells** | **Neutrophils** | **NK.CD56dim.cells** | **NK.cells** | **T.cells** | **Th1.cells** | **Treg** | **CD8.T.cells** | **CD4.cells** |
| --- | --- | --- | --- | --- | --- | --- | --- | --- | --- | --- | --- | --- | --- | --- | --- | --- | --- | --- | --- | --- | --- | --- | --- |
| TCGA-05-4244 | yes | 12 | G12C | NA | NA | NA | RAG-3 | 11.5104282 | -1.1792902 | -0.1937459 | -0.1064273 | 0.51601163 | -0.4821236 | 0.41009749 | -0.5660662 | 0.54545218 | -0.3198486 | -0.2963593 | -0.5005182 | -0.3687956 | -0.471533 | -0.5165227 | 0.04639621 |
| TCGA-05-4249 | yes | 12 | G12C | NA | NA | NA | RAG-2 | 11.2097921 | 0.33707895 | 0.48692637 | -1.0239789 | 0.3887352 | -0.1803321 | 0.11518654 | 1.91767025 | 0.10037201 | 0.03622301 | -0.205094 | -0.5008013 | -0.5309933 | 0.31868281 | -0.6886913 | 0.20690535 |
| TCGA-05-4250 | yes | 12 | G12C | NA | NA | NA | RAG-4 | 12.0779719 | -0.7859089 | -0.0004034 | 1.09574757 | 0.82899868 | 0.69706876 | 0.96865366 | -0.6400474 | 0.1253822 | -0.0299514 | 0.26090412 | 0.54270629 | 0.14478716 | -0.6230978 | 1.53584399 | 0.54470312 |
| TCGA-05-4403 | yes | 12 | G12C | NA | NA | NA | RAG-4 | 11.7832144 | -0.4344508 | -0.0458972 | -0.067764 | 1.30506448 | -0.0098913 | 1.07714462 | 0.64273154 | 0.68566563 | -0.0837702 | 0.00899009 | -0.1447873 | -0.4024459 | -0.5707524 | -0.5359964 | 0.68470354 |
| TCGA-05-4405 | yes | 12 | G12C | NA | NA | NA | RAG-2 | 11.2171673 | 0.15239034 | -0.2411433 | -0.6922097 | -0.5461434 | -0.1914794 | -0.2381248 | 0.61099176 | -0.1081943 | -0.2933673 | -0.210978 | -0.6656416 | -0.6664995 | -0.8315557 | -0.2627837 | 0.23565315 |
| TCGA-05-4415 | yes | 12 | G12C | NA | L228Rfs*33 | NA | RAG-4 | 11.4274849 | -1.2010777 | -0.7220751 | 0.67619892 | -0.4127429 | 0.22158186 | -0.37457 | -0.7605606 | 0.10794712 | -0.2180547 | 0.39003329 | -0.503412 | 0.1085592 | -0.6252686 | 0.43551253 | 0.3372729 |
| TCGA-05-4417 | yes | 12 | G12C | NA | H168R | L2088= | RAG-1 | 11.2245051 | 1.11571048 | 1.05017058 | 0.80032047 | 1.5199272 | 0.41857064 | 0.58123936 | 0.14848007 | 0.71996513 | 0.42487736 | 0.23754693 | 1.09719069 | 0.32821994 | 1.50959646 | 0.73658449 | 0.48945403 |
| TCGA-05-4418 | yes | 12 | G12C | R460G | NA | NA | RAG-4 | 11.5528713 | 1.31245799 | 0.32730841 | 0.67437334 | 0.62187677 | 0.14574239 | 0.63131278 | -0.354992 | 0.40614539 | 0.27713957 | 0.32488239 | 0.70474927 | -0.1674918 | 1.06121183 | 0.85002423 | 0.45169779 |
| TCGA-35-3615 | yes | 12 | G12C | E449* | X245_splice | NA | RAG-1 | 10.8707615 | -0.1891401 | -0.7411983 | -0.4633526 | 0.81888302 | -0.5247609 | 0.03997669 | 0.04410868 | -0.3010718 | -0.2526954 | -0.043782 | -0.4675156 | -0.5395273 | -0.5324844 | -0.2476316 | 0.11600071 |
| TCGA-44-7659 | yes | 12 | G12C | NA | NA | NA | RAG-2 | 11.1554395 | 0.79652744 | -0.3481378 | -0.3998125 | -0.4125036 | -0.4707573 | -0.5831647 | 0.59553103 | -0.649766 | -0.2261746 | -0.3476188 | -0.4540109 | -0.1555659 | -0.3940045 | -0.814248 | -0.8634132 |
| TCGA-44-7671 | yes | 12 | G12C | P278S | K262* | NA | RAG-1 | 11.0968314 | -1.0824246 | -1.4973754 | -1.3687571 | 0.03393267 | -0.6246379 | -0.554296 | 0.53631809 | -0.9108376 | -0.4862784 | -0.5021333 | -1.404591 | -0.8183674 | -1.5033395 | -1.4750161 | -0.5924667 |
| TCGA-49-4505 | yes | 12 | G12C | NA | NA | NA | RAG-3 | 11.4378414 | 0.4383359 | -0.3211922 | 0.80383237 | 0.51446661 | 0.2634009 | -0.0096339 | 2.75348654 | 0.3678647 | 0.03593877 | 0.09854656 | 0.37140002 | 0.11627512 | 0.68652124 | 1.12553009 | 0.31048551 |
| TCGA-4B-A93V | yes | 12 | G12C | L199F | Q170P | NA | RAG-1 | 10.8212432 | 0.45679692 | -1.1398038 | 0.6947956 | -1.1433018 | 0.43579459 | -1.1494416 | -2.630499 | -0.706309 | 0.30606548 | -0.1970431 | -0.1016446 | 0.34606621 | -0.7759308 | -0.4142509 | -0.7533105 |
| TCGA-50-5051 | yes | 12 | G12C | G417R | NA | NA | RAG-1 | 10.8440419 | 0.57059235 | -1.606636 | -0.8650003 | -1.6641669 | -0.9942826 | -2.1052458 | 0.15728898 | -0.4770164 | -0.42251 | -0.3088635 | -1.0881182 | -0.6773394 | -0.926602 | -0.6390225 | -1.3338889 |
| TCGA-50-5932 | yes | 12 | G12C | NA | NA | NA | RAG-1 | 10.7864231 | -0.9637247 | -1.0708908 | -0.9284561 | -0.4923595 | -0.9283504 | -0.5333227 | -1.4735735 | -0.5421389 | -0.4995448 | -0.293337 | -1.3481515 | -0.633467 | -1.553771 | -1.5438221 | -0.3351809 |
| TCGA-50-5933 | yes | 12 | G12C | NA | NA | NA | RAG-4 | 11.621686 | -1.295717 | 0.92716567 | -0.2148734 | 2.28831479 | -0.227404 | 1.36104991 | 0.22640285 | 0.19706952 | 0.05702429 | -0.2999729 | -0.444971 | -0.6866693 | 0.58822927 | -0.4380068 | 1.35439104 |
| TCGA-50-5936 | yes | 12 | G12C | Q46* | NA | NA | RAG-4 | 11.2389519 | -0.2142081 | -0.0240753 | -0.9047076 | 0.15045397 | -0.7218986 | 0.60022261 | -0.4476231 | 0.53274298 | -0.1503955 | -0.2934441 | -0.3251039 | -0.7500088 | 0.86977238 | -1.1431098 | 0.15559647 |
| TCGA-50-7109 | yes | 12 | G12C | NA | S193=;D194Y | NA | RAG-1 | 10.6547137 | 0.41089419 | 0.10342867 | 0.77830418 | -1.1285475 | -0.0224779 | -0.0359882 | -1.3146753 | 0.61417094 | 0.04459683 | 0.08256736 | 0.39909952 | 0.26046551 | -0.3404242 | 0.51451571 | -0.0674675 |
| TCGA-50-8459 | yes | 12 | G12C | NA | NA | NA | RAG-4 | 11.3858926 | 1.31207871 | 1.14981004 | -0.1246137 | 0.91800552 | 0.21012254 | 1.5006594 | 0.70418366 | 0.38278361 | 0.11588635 | -0.0926219 | 0.33381055 | -0.0119052 | 1.12349079 | -0.0291444 | 0.72436209 |
| TCGA-55-1595 | yes | 12 | G12C | NA | NA | NA | RAG-2 | 10.9877233 | 0.24835732 | -0.2207908 | -0.0909103 | -0.8883787 | 0.0693089 | -0.5437746 | -1.3312438 | -0.2278485 | 0.07846904 | -0.1089722 | -0.0081069 | 0.21977394 | 0.46699968 | 0.31915367 | 0.02578434 |
| TCGA-55-6983 | yes | 12 | G12C | NA | NA | NA | RAG-1 | 11.1684939 | 2.047936 | 1.0798724 | 0.52522011 | -0.5645331 | 0.05616347 | -0.1662655 | 0.4647144 | -0.0100746 | 0.05244184 | -0.1979196 | 0.93335622 | 0.35569169 | -0.1708206 | 0.6085659 | -0.4671257 |
| TCGA-55-7281 | yes | 12 | G12C | NA | NA | NA | RAG-3 | 11.8388474 | -0.9004107 | -0.0380089 | 0.21979598 | 0.1725367 | -0.0900517 | 0.21234867 | -0.4887347 | 0.22634055 | 0.2173829 | 0.04878343 | -0.1755818 | -0.0429997 | 1.30311252 | 0.19683403 | 0.4536783 |
| TCGA-55-7726 | yes | 12 | G12C | NA | NA | NA | RAG-4 | 11.8209904 | -0.7910485 | -0.1299621 | -1.0631835 | 0.97972208 | -0.4826985 | -0.2421951 | -0.1956315 | -0.2921014 | -0.2045221 | -0.0946868 | -0.9561917 | -0.9015115 | -0.4872396 | -1.287133 | -0.1167672 |
| TCGA-55-7907 | yes | 12 | G12C | NA | NA | D2482N | RAG-3 | 11.1868161 | -0.027117 | 0.44765288 | 0.79364578 | -0.3522242 | 0.64516942 | 0.18957569 | -0.2059186 | -0.0187316 | 0.31769074 | 0.36488219 | 0.52811134 | 0.66774245 | 0.53158857 | 0.66940106 | 0.22090744 |
| TCGA-55-7914 | yes | 12 | G12C | NA | NA | NA | RAG-2 | 10.8965613 | 1.14049976 | -0.057006 | 0.13488082 | -1.4132636 | -0.0465779 | -1.100732 | 0.7203145 | -0.5755124 | -0.0881583 | -0.0709744 | 0.24526421 | 0.03841608 | -0.729327 | 0.67244352 | -0.3841986 |
| TCGA-55-8097 | yes | 12 | G12C | NA | NA | NA | RAG-2 | 11.0091236 | 0.3414024 | 0.00423642 | -0.8520956 | 0.37275834 | -0.3029125 | -0.4585105 | 1.98357195 | -0.1947141 | 0.00169944 | -0.4822088 | 0.1554154 | -0.0405329 | -0.1682624 | -0.7240283 | -0.4091053 |
| TCGA-55-8203 | yes | 12 | G12C | NA | G56W | NA | RAG-1 | 10.8339938 | 1.81783449 | 0.45299499 | 0.25860827 | -0.0315905 | 0.33987079 | -0.4094528 | -1.2308514 | 0.22481042 | 0.21482734 | 0.31714963 | 0.54918097 | 0.40394989 | 0.20442802 | 0.69859763 | -0.4594862 |
| TCGA-55-8207 | yes | 12 | G12C | NA | NA | NA | RAG-3 | 11.7184303 | -0.6401888 | 0.76638816 | -0.4337386 | 0.64130107 | -0.1541031 | 0.78123042 | 1.7598086 | 0.86626998 | -0.3731406 | -0.3516613 | -0.3150199 | -0.1252556 | -0.1933199 | -0.1760854 | 0.18112227 |
| TCGA-55-8299 | yes | 12 | G12C | NA | E223* | NA | RAG-3 | 11.4518663 | 0.56991463 | 1.08897542 | 2.36224751 | 0.90591712 | 1.2625228 | 0.98586461 | 1.71098699 | 1.09970428 | 0.98483945 | 0.70506034 | 1.31057633 | 1.55636651 | 0.54490495 | 2.08816833 | 0.67777515 |
| TCGA-55-8302 | yes | 12 | G12C | G417V | NA | NA | RAG-4 | 11.4555765 | 0.16398617 | -0.934188 | 0.09581118 | -0.1758841 | -0.1654368 | -0.3080592 | 0.55331125 | -0.3873692 | 0.02329816 | 0.03653793 | -0.4104497 | 5.04397092 | -0.3866005 | -0.0020678 | 0.22102209 |
| TCGA-55-8508 | yes | 12 | G12C | NA | D194Y | NA | RAG-1 | 10.9970899 | 1.42859285 | -0.3598264 | 0.0932017 | -1.0601648 | -0.3933691 | -0.7270505 | -0.7732501 | 1.21535417 | 2.41439104 | -0.3613203 | -0.4217137 | -0.1712508 | 0.38321595 | -0.459698 | -0.748125 |
| TCGA-55-8615 | yes | 12 | G12C | NA | Q220* | NA | RAG-1 | 10.7637081 | -1.0556602 | -2.1919687 | -1.169946 | -0.5799895 | -1.0709267 | -0.8781008 | -1.9929897 | 0.07002354 | -0.4454949 | -0.015358 | -1.3243494 | -0.9705551 | -1.0990319 | -2.0474565 | -0.6841743 |
| TCGA-55-8616 | yes | 12 | G12C | NA | NA | NA | RAG-2 | 10.9878084 | 0.81130242 | -0.6670363 | -1.1289747 | -1.0802483 | -0.37041 | -0.5497065 | 0.56801985 | -0.3344453 | -0.5781545 | -0.5796075 | -0.1455928 | -0.2828446 | -1.2670522 | -0.1891249 | -0.737825 |
| TCGA-55-A490 | yes | 12 | G12C | NA | NA | NA | RAG-1 | 11.1775177 | -0.6751097 | -1.5962968 | -0.8373586 | 0.01932435 | -0.5838894 | 0.00075494 | -1.5119321 | -0.4683522 | -0.3094662 | -0.573898 | 0.0950683 | -0.1430108 | 0.69060247 | -0.7949919 | 0.33813546 |
| TCGA-64-1677 | yes | 12 | G12C | NA | NA | NA | RAG-3 | 11.1146658 | 1.52994132 | -0.4148075 | 0.88341694 | -0.8240255 | -0.0750666 | -1.4513961 | -1.0271385 | -0.3956488 | -0.0200871 | 0.82387722 | 0.55568631 | -0.2354171 | -0.3671632 | 0.49011704 | -0.7567356 |
| TCGA-64-5774 | yes | 12 | G12C | NA | NA | NA | RAG-1 | 10.7245483 | -0.4252483 | -1.7074795 | -1.0722696 | -1.1900046 | -0.9161333 | -2.0880341 | -2.2657944 | 0.06666924 | 2.20845779 | 0.45140816 | -0.8271705 | -0.9483116 | -0.076236 | 0.81676958 | -1.069194 |
| TCGA-64-5778 | yes | 12 | G12C | NA | NA | NA | RAG-3 | 10.8699057 | 1.38670902 | 0.73899142 | 1.70904219 | -0.6630655 | 0.82570962 | -0.0741839 | -2.0292883 | -0.5029364 | 0.19730962 | 0.90725905 | 1.59969847 | 1.7028291 | 0.91805258 | 2.51871779 | -0.0495003 |
| TCGA-69-7973 | yes | 12 | G12C | NA | NA | NA | RAG-1 | 10.6620834 | -0.0285952 | -0.7835551 | -0.4985565 | -0.4943759 | -0.4901377 | -0.5476523 | -0.3787487 | 0.32117811 | 0.69285997 | -0.5629418 | -0.6145714 | -0.7430715 | -0.2915751 | 0.68768083 | -0.6008299 |
| TCGA-69-7978 | yes | 12 | G12C | NA | NA | L2413Ffs*5 | RAG-4 | 11.3786961 | 0.70881393 | 1.53068365 | 1.2868859 | 2.27615022 | 1.19932047 | 1.6311295 | 0.95467097 | 0.32161683 | 0.76607643 | 0.67182835 | 1.17731365 | 1.51163823 | 1.56157293 | 1.36260341 | 1.25920272 |
| TCGA-73-4662 | yes | 12 | G12C | NA | NA | NA | RAG-2 | 11.1497792 | 1.20104854 | 0.72215779 | -0.1879896 | 0.61549736 | 0.13355673 | 0.34354987 | -0.0105742 | -0.0828144 | 0.04424004 | 0.06532508 | 0.6356545 | 0.14553969 | 1.01245859 | 0.41928935 | -0.1813755 |
| TCGA-73-7498 | yes | 12 | G12C | E218V | D53Tfs*11 | NA | RAG-1 | 10.5259752 | 0.26538238 | -0.1603946 | -0.4173718 | 0.06511421 | -0.1745121 | -0.5192165 | 1.14625555 | -0.1909054 | -0.0121129 | -0.1918578 | 0.09930296 | 0.14585569 | -0.0536425 | -0.7679425 | -0.7513286 |
| TCGA-75-5126 | yes | 12 | G12C | NA | NA | NA | RAG-4 | 11.8554339 | -0.5875572 | 1.1660911 | 0.99715759 | 3.10449841 | 0.74300502 | 2.08553125 | 1.24961124 | 1.8962841 | 0.31488583 | 0.42880962 | 0.27375188 | -0.1449566 | -0.3179037 | 1.27979528 | 1.41602068 |
| TCGA-78-7148 | yes | 12 | G12C | NA | E265Gfs*19 | NA | RAG-1 | 11.1528297 | -0.9060546 | -0.4459448 | 0.48555095 | -0.2335877 | -0.2183817 | -0.8492217 | -1.0882954 | -0.721535 | -0.1540078 | 0.18413418 | 0.09231313 | 0.23278954 | 0.12709714 | 0.63800917 | -0.3700252 |
| TCGA-78-7166 | yes | 12 | G12C | F280Y | G56V;K78E | NA | RAG-1 | 11.3665744 | 0.25335451 | -1.658462 | 0.2472083 | -1.5640841 | -0.6091833 | -2.2259128 | -1.4221318 | -0.8445858 | 0.10147541 | 0.26896223 | -0.3609118 | 0.10089437 | -0.7695069 | -0.8884268 | -1.4671339 |
| TCGA-78-7539 | yes | 12 | G12C | NA | NA | NA | RAG-2 | 10.7525502 | -0.3256136 | 0.62306083 | 0.84080945 | -0.3881529 | 0.55820669 | -0.0953503 | -0.5493972 | -0.529849 | 0.06536733 | 0.32036124 | 1.05060507 | 0.56573451 | 0.4530649 | 1.33388404 | 0.054344 |
| TCGA-86-7713 | yes | 12 | G12C | NA | L245F | NA | RAG-1 | 10.9247464 | -0.6659594 | -1.0865327 | -1.1107524 | -0.8736933 | -0.7961122 | -0.4801935 | -2.3651442 | 0.17612525 | -0.0712781 | -0.3369558 | -0.9735197 | -0.7555227 | -0.7427352 | -1.4053421 | -0.6846336 |
| TCGA-91-6849 | yes | 12 | G12C | A159P | NA | NA | RAG-3 | 11.3823059 | 1.34987685 | 0.59097478 | 0.35666431 | 0.44278938 | 0.14116071 | 0.80204972 | -1.2138606 | 0.22601253 | 0.01454676 | -0.3972217 | 0.21401563 | 0.48437412 | -0.7388328 | 0.24694721 | -0.4840458 |
| TCGA-93-7347 | yes | 12 | G12C | NA | NA | NA | RAG-3 | 11.0939474 | 0.86147433 | 1.44501548 | 1.08319666 | 1.18033813 | 1.03529172 | 0.72669876 | 1.61067622 | 0.39874752 | 0.58599116 | 0.60555066 | 1.93658274 | 1.0728997 | 2.14724282 | 1.75114727 | 0.43736393 |
| TCGA-95-7562 | yes | 12 | G12C | NA | NA | NA | RAG-0 | 10.7472205 | -0.6645986 | -0.3375512 | -0.6575481 | -0.8594128 | -0.001564 | -0.1434601 | -0.4168783 | -0.5362354 | -0.1253931 | -0.150392 | -0.1877535 | -0.1719575 | -0.0476291 | -0.2094452 | 0.06520602 |
| TCGA-97-7938 | yes | 12 | G12C | NA | NA | NA | RAG-3 | 11.830146 | -0.0877904 | -0.0287131 | -0.657429 | -0.6851178 | -0.2825778 | -0.4815722 | 1.51394385 | -0.1437936 | -0.283549 | -0.1929715 | -0.3111825 | -0.3148131 | -0.9044744 | 0.12540107 | -0.4326081 |
| TCGA-97-A4M0 | yes | 12 | G12C | NA | NA | NA | RAG-3 | 10.8572165 | 0.00126411 | 0.69768642 | 0.62092093 | -0.2160835 | 0.44161332 | -0.1665073 | 0.62343662 | -0.2401557 | 0.14651452 | 0.05828175 | 0.79555861 | 0.44689419 | 0.76046317 | 0.98872866 | -0.1365872 |
| TCGA-97-A4M5 | yes | 12 | G12C | NA | NA | NA | RAG-1 | 10.9881523 | 0.38783886 | 0.30532257 | 0.42966253 | 0.42471824 | 0.35226019 | 0.62545464 | 1.61562508 | 0.2614869 | 0.3381496 | -0.0046983 | -0.3492135 | 0.97805597 | -0.0480467 | -0.7902447 | 0.38382075 |
| TCGA-99-8028 | yes | 12 | G12C | NA | NA | NA | RAG-3 | 11.5947299 | 1.62754231 | 1.35990041 | 2.27107688 | 1.66021815 | 1.43849822 | 1.17521946 | -0.4682239 | 0.50568279 | 0.72580893 | 1.07292501 | 2.05391798 | 1.9646954 | 1.71483841 | 2.1471062 | 1.0076568 |
| TCGA-99-8032 | yes | 12 | G12C | NA | M136K;M136I | NA | RAG-1 | 10.7750111 | 1.00668181 | -0.4257882 | -0.4438989 | -0.0847417 | -0.5581269 | -0.7338437 | 0.90799113 | 0.17394786 | -0.2658086 | -0.0686955 | -0.2545524 | -0.4151583 | 0.19775522 | -0.5231674 | -0.530029 |
| TCGA-J2-8194 | yes | 12 | G12C | E540Rfs*32 | K146* | NA | RAG-1 | 11.2901484 | -0.7947879 | 0.12636071 | -0.8946498 | 0.38161023 | -0.7122679 | 0.31317112 | -1.5443683 | 1.2278734 | -0.0436563 | -0.3974551 | -0.9207038 | -0.7602975 | -0.8486149 | -1.4555959 | -0.3971487 |
| TCGA-MN-A4N5 | yes | 12 | G12C | NA | NA | NA | RAG-3 | 11.2858492 | 0.04104818 | -0.3845457 | 1.28747502 | -1.1850428 | 0.4637106 | -1.041391 | -1.2341073 | -0.5553247 | 0.16374731 | 0.210587 | 0.1610995 | 1.18650646 | -0.2913156 | 0.95867219 | -0.26085 |
| TCGA-MP-A4TD | yes | 12 | G12C | NA | E57Kfs*7 | NA | RAG-1 | 10.9643789 | 1.68122157 | -0.2908575 | -0.3505876 | -0.034705 | -0.0432959 | -0.673816 | 0.55000752 | -0.2464765 | 0.04009771 | -0.0483895 | -0.0218125 | 0.24563975 | 0.06904941 | -0.7008058 | -0.4747203 |
| TCGA-MP-A4TE | yes | 12 | G12C | V604Wfs*68 | G251C | NA | RAG-1 | 10.7774877 | -0.2742928 | -2.383374 | -1.3065072 | -1.0807846 | -1.3072311 | -2.1326122 | -2.4022887 | 0.59912301 | -0.5919702 | -0.6451082 | -1.2494109 | -0.8686393 | -1.4144521 | -1.5431964 | -1.3047671 |
| TCGA-MP-A4TF | yes | 12 | G12C | NA | NA | NA | RAG-4 | 11.570515 | -0.1910645 | -1.3053903 | 0.01991724 | -0.8193843 | -0.6896141 | -1.4339954 | -1.1292517 | 0.11921318 | -0.1844889 | -0.1870501 | -0.9857839 | -0.1671135 | -0.5951837 | -0.4291214 | -0.6130247 |
| TCGA-MP-A4TI | yes | 12 | G12C | S166= | NA | NA | RAG-4 | 11.4020041 | 0.54759316 | 1.5241064 | 1.93259348 | 2.24842097 | 1.7266482 | 1.58039618 | 1.63782557 | 0.58012888 | 1.00940274 | 0.64642104 | 1.47636429 | 1.84214045 | 2.18891516 | 1.31036539 | 1.0462116 |
| TCGA-NJ-A4YI | yes | 12 | G12C | NA | NA | NA | RAG-2 | 11.0648088 | -1.3871971 | -0.2271918 | -1.1033793 | -1.1143909 | -0.5436508 | 0.12336745 | -1.1608969 | -0.1033565 | -0.4737574 | -0.4460706 | -1.2206585 | -1.2167921 | -0.4667014 | -1.8194852 | -0.0463948 |
| TCGA-NJ-A55O | yes | 12 | G12C | NA | S69* | NA | RAG-1 | 11.2623989 | -0.1551552 | -0.3029165 | -0.685199 | 0.66713396 | -0.4225272 | 0.94862969 | -0.271641 | -0.0736786 | -0.1884525 | -0.4965409 | -0.3285168 | -0.0628874 | -0.6637078 | -0.894918 | 0.43868615 |
| TCGA-44-6776 | yes | 12 | G12D | V155A | Q220* | NA | RAG-1 | 10.7831187 | -0.8052521 | -1.3812761 | -0.6317937 | -0.3797822 | -0.8179856 | -1.4514056 | 0.64866231 | -0.5784358 | -0.3430287 | -0.2138631 | -0.9824212 | -0.7011637 | -1.1267396 | -0.8390088 | -1.0557961 |
| TCGA-44-A47A | yes | 12 | G12D | NA | NA | NA | RAG-2 | 10.9275769 | -0.6156194 | 0.39696642 | -0.4504312 | 0.74597556 | -0.4214695 | 0.3000955 | 0.85861078 | -0.3268401 | 0.07020648 | -0.5520542 | 0.06095555 | -0.3450435 | 1.73416308 | -0.5581315 | -0.1737261 |
| TCGA-49-4510 | yes | 12 | G12D | NA | NA | NA | RAG-1 | 10.9512094 | -0.268371 | -1.810792 | 0.03245558 | 0.01741353 | -0.3986868 | -0.2956974 | -0.8539068 | -0.0345063 | -0.098358 | -0.1930907 | -0.7205134 | -0.5405517 | -1.1008911 | -0.1874923 | -0.6322043 |
| TCGA-55-7284 | yes | 12 | G12D | NA | NA | NA | RAG-4 | 11.6394664 | -0.574258 | 0.57250431 | 0.10921145 | -0.1298617 | -0.0230655 | 0.68284275 | 0.48910354 | 0.2228821 | -0.0434692 | 0.11075965 | -0.1196803 | 0.29216051 | -0.5222074 | 0.14239548 | -0.0520765 |
| TCGA-55-A494 | yes | 12 | G12D | NA | NA | NA | RAG-1 | 11.0927704 | -1.7664801 | -3.8309373 | -1.6838751 | -1.3424751 | -1.2729648 | -2.798232 | -3.3267213 | -0.5964265 | -0.7720586 | -0.4762521 | -0.8302654 | -0.7625257 | -1.5323724 | -0.6239513 | -1.1910737 |
| TCGA-62-8398 | yes | 12 | G12D | G430V | NA | NA | RAG-4 | 11.6813802 | -0.7464478 | 0.15942969 | -0.1705667 | 0.02560442 | -0.4893561 | 0.40236837 | -0.8214886 | 0.86204921 | 0.03413473 | -0.3191813 | -0.1348451 | -0.7359721 | 0.55778331 | -0.5659033 | -0.2901628 |
| TCGA-69-8253 | yes | 12 | G12D | E41* | NA | NA | RAG-1 | 11.4219373 | 0.58567234 | -0.8101018 | -0.3280141 | -0.6312553 | -0.9266031 | -0.4133572 | -0.1804583 | 0.0884326 | -0.2984385 | -0.304109 | -0.5835031 | -0.4755667 | -0.5998766 | -1.0082735 | -0.6806017 |
| TCGA-69-8254 | yes | 12 | G12D | NA | NA | NA | RAG-3 | 11.915618 | 0.96479099 | 0.12804183 | -0.0020398 | 0.49133441 | 0.79172091 | -0.0007549 | 0.50934339 | -0.0315747 | 0.02117207 | -0.0323026 | 0.36813262 | 0.60013451 | 0.22783746 | -0.3005075 | 0.03144397 |
| TCGA-75-7030 | yes | 12 | G12D | NA | NA | NA | RAG-4 | 11.9322077 | 1.22472164 | 0.52993786 | -0.0283385 | -0.0536602 | 0.06174584 | -0.449484 | 1.78779942 | 0.0851115 | -0.0612507 | -0.2942529 | 0.67208843 | 0.50944031 | 0.27661789 | 0.56248277 | -0.3690952 |
| TCGA-78-7160 | yes | 12 | G12D | NA | D53Tfs*11 | E806* | RAG-1 | 11.1949262 | 0.0228883 | 1.1938931 | 0.73755267 | 0.41864866 | 0.7103244 | 0.86299765 | -0.3694202 | 0.11933977 | 0.79736893 | 0.3218082 | 1.08075411 | 0.76257122 | 0.88784708 | 1.43539625 | 0.31873957 |
| TCGA-86-7953 | yes | 12 | G12D | NA | NA | NA | RAG-3 | 11.348416 | -0.0842452 | 0.4673186 | -0.1296179 | -0.2639946 | -0.1456216 | 0.78139477 | -1.7741485 | 0.71475323 | 0.02848033 | -0.1273255 | 0.19031034 | -0.408366 | 0.83106829 | -0.2186939 | 0.24603651 |
| TCGA-86-8056 | yes | 12 | G12D | NA | NA | NA | RAG-3 | 11.3941256 | -0.0394103 | 0.55083376 | -0.5526928 | -0.2170507 | 0.35183082 | 0.19511993 | 0.91782328 | 0.43089269 | -0.1577262 | -0.2743965 | -0.2350714 | -0.2506281 | -0.1456169 | -0.6002262 | 0.02656002 |
| TCGA-86-8076 | yes | 12 | G12D | NA | NA | NA | RAG-1 | 11.1220748 | 1.64435999 | 1.26659034 | 1.192316 | -0.1819454 | 0.73507921 | 0.11422737 | 0.92693726 | 0.83045843 | 0.24613039 | 0.80937288 | 1.29298462 | 0.8716482 | 0.46613701 | 1.83004938 | -0.3495508 |
| TCGA-93-A4JO | yes | 12 | G12D | NA | NA | NA | RAG-3 | 11.1318998 | 1.57758029 | 0.95669081 | 0.8032514 | -0.4823592 | 0.71454659 | -0.1428875 | 0.75238844 | 0.19335886 | 0.61815009 | 0.41367831 | 1.07251903 | 0.84580805 | 0.71349329 | 1.06171687 | 0.0624604 |
| TCGA-J2-A4AG | yes | 12 | G12D | NA | NA | NA | RAG-3 | 11.9089068 | 1.66371646 | 0.73130334 | 0.23052114 | 0.2718016 | 0.38976494 | -0.2804833 | -0.1502759 | 0.13196685 | 0.30576151 | -0.2754449 | 0.65340141 | 0.44282506 | 0.63018259 | 0.04436047 | -0.260443 |
| TCGA-MP-A4SY | yes | 12 | G12D | NA | NA | D946Y | RAG-3 | 11.4803439 | -1.1496081 | -0.7448909 | -1.3265701 | -0.3891831 | -0.7768784 | 0.09768759 | 0.19254068 | -0.4329453 | -0.1264528 | -0.4275538 | -1.2130085 | -0.4362974 | -0.2954925 | -1.6950687 | 0.14005181 |
| TCGA-MP-A4T8 | yes | 12 | G12D | NA | NA | NA | RAG-1 | 11.0772895 | -1.2705915 | -1.9847959 | -0.7770145 | -0.936477 | -1.0764806 | -1.4322613 | -1.4274128 | -0.1251548 | -0.3739443 | -0.619434 | -1.0918318 | -0.8052096 | -0.2393231 | -1.8751978 | -0.9623926 |
| TCGA-NJ-A4YG | yes | 12 | G12D | NA | NA | NA | RAG-2 | 11.3278144 | -0.3205747 | -0.5598013 | -0.3763971 | -0.1213146 | -0.3826696 | -0.1341493 | 0.57466546 | -0.0570182 | 0.037782 | -0.0566576 | -0.1710893 | -0.0900016 | 1.12923373 | -0.6344216 | -0.1649296 |
| TCGA-55-6975 | yes | 13 | G13C | NA | NA | F2382L | RAG-4 | 11.2001308 | 0.10121385 | -0.7012797 | -0.7594219 | -0.5291771 | -0.3683346 | -0.6262064 | -0.1105854 | -0.0070193 | -0.0495688 | -0.2090593 | -0.5380514 | -1.0048297 | -0.6069383 | -0.4507658 | -0.3014686 |
| TCGA-55-7910 | yes | 13 | G13C | NA | NA | NA | RAG-1 | 10.8879711 | 0.38980355 | -0.6389884 | -0.7882636 | -0.9805312 | -0.8584423 | -1.5851087 | -0.8704396 | 0.32243939 | -0.2858436 | -0.1677322 | -0.6133937 | -0.7039795 | -0.4087799 | -1.429137 | -0.737236 |
| TCGA-55-8094 | yes | 13 | G13C | Y162C | NA | NA | RAG-1 | 11.084872 | -1.3096579 | -3.2762352 | -1.5176073 | -1.7027239 | -1.3977679 | -1.2675574 | -2.7108227 | -0.4376393 | -0.6972844 | 0.40639382 | -1.4901799 | -1.1438005 | -1.2371111 | -1.7033536 | -1.1452549 |
| TCGA-86-8674 | yes | 13 | G13C | G476V | NA | NA | RAG-1 | 11.0586348 | -0.8456545 | -3.241138 | -0.8342438 | -2.1025135 | -1.3973652 | -2.5763389 | -2.2405211 | -1.0182342 | -0.386152 | -0.5269442 | -1.2842599 | -0.8431439 | -1.0585451 | -1.4900643 | -1.6009773 |
| TCGA-99-8025 | yes | 13 | G13C | NA | NA | NA | RAG-2 | 11.3187623 | 0.15686277 | -1.5785608 | -1.1718824 | -1.0868219 | -0.4787655 | -1.4848807 | -0.7017461 | 0.03083918 | -0.2384703 | -0.4642257 | -0.9398903 | -0.8077298 | -0.3551296 | -1.3347683 | -0.4255428 |
| TCGA-99-8033 | yes | 13 | G13C | R554Q | NA | NA | RAG-3 | 11.5752366 | -0.5826816 | -0.0399213 | 1.95839054 | -0.4541668 | 0.55496227 | -0.6071077 | -0.5838345 | 0.51140566 | 0.21221268 | 0.70734328 | 0.32056401 | 0.94131326 | -0.9528917 | 1.0549332 | -0.1703146 |
| TCGA-49-4506 | yes | 13 | G13D | M503K | NA | NA | RAG-4 | 11.5253262 | 0.87349794 | -0.0199702 | 2.04535683 | 0.0559241 | 0.64684225 | -0.2058485 | -1.9770923 | 0.2085659 | 0.14528723 | 0.27007939 | 0.9161536 | 0.31380697 | -0.2152546 | 2.60211429 | -0.3709378 |
| TCGA-62-A46S | yes | 13 | G13D | NA | K78N | X574_splice | RAG-1 | 11.20708 | -0.0622765 | -0.3119501 | 0.02836216 | -0.254989 | -0.2453275 | -0.1790111 | -0.4117401 | -0.0742503 | 0.43682388 | -0.0112603 | 0.02809631 | 0.10883909 | -0.6813997 | -0.3201313 | -0.0844933 |
| TCGA-75-6206 | yes | 13 | G13D | V214L | NA | NA | RAG-1 | 10.9131069 | -1.1415289 | -0.3646634 | -0.8552019 | 0.75055408 | -0.4749501 | 1.07211508 | -0.4356158 | -0.071357 | -0.1514003 | -0.5095432 | -0.9759461 | -0.6883124 | -0.8995265 | -1.0904547 | -0.0102955 |
