# Supplementary material for: Targeting KRAS Codon 13 Mutations Using Direct Combination Approaches in Non–Small Cell Lung Cancer
Source: Cancer Discov. 2026 Jun 22;16(11):2274–97. doi: 10.1158/2159-8290.CD-25-0884 (PMC13725345; doi:10.1158/2159-8290.CD-25-0884)
Supplement: Supplementary file 5 [file cd-25-0884_supplementary_tables_s1-s4_suppst1.docx]

**Supplementary Table 1 - Baseline characteristics of RAS-PM patients**

| **Baseline characteristics** | **KRAS^G12C^** | **KRAS^G12D^** | **KRAS^G13C^** | **KRAS^G13D^** | **KRAS^G12^ others** | **KRAS^G13^ others** |
| --- | --- | --- | --- | --- | --- | --- |
| **No. Patients** | 309 | 133 | 39 | 38 | 212 | 6 |
| **Age at baseline, median years, (range)** | 66.0 (39 – 88) | 63.0 (19 – 92) | 66.0 (45 – 81) | 63.5 (41 – 82) | 65.0 (34 – 85) | 68.5 (53 – 84) |
| **Sex:** |  |  |  |  |  |  |
| **Female, N (%)** | 181 (58.5%) | 68 (51.1%) | 23 (59.0%) | 20 (52.6%) | 104 (49.1%) | 2 (33.3%) |
| **Male, N (%)** | 128 (41.5%) | 65 (48.9%) | 16 (41.0%) | 18 (47.4%) | 108 (50.9%) | 4 (66.7%) |
| **ECOG PS:** |  |  |  |  |  |  |
| **0, N (%)** | 84 (27.2%) | 41 (30.8%) | 6 (15.4%) | 6 (15.8%) | 51 (24.1%) | 0 (0%) |
| **1, N (%)** | 170 (55.0%) | 59 (44.4%) | 19 (48.7%) | 19 (50.0%) | 127 (59.9%) | 4 (66.7%) |
| **≥2, N (%)** | 42 (13.6%) | 23 (17.3%) | 11 (28.2%) | 10 (26.3%) | 21 (9.9%) | 2 (33.3%) |
| **Unknown, N (%)** | 13 (4.2%) | 10 (7.5%) | 3 (7.7%) | 3 (7.9%) | 13 (6.1%) | 0 (0%) |
| **Smoking status:** |  |  |  |  |  |  |
| **Ex-smoker, N (%)** | 223 (72.2%) | 75 (56.4%) | 27 (69.2%) | 22 (57.9%) | 133 (62.7%) | 5 (83.3%) |
| **Current, N (%)** | 66 (21.4%) | 33 (24.8%) | 11 (28.2%) | 13 (34.2%) | 56 (26.4%) | 0 (0%) |
| **Never, N (%)** | 17 (5.5%) | 25 (18.8%) | 1 (2.6%) | 3 (7.9%) | 21 (9.9%) | 1 (16.7%) |
| **Unknown, N (%)** | 3 (0.9%) | 0 (0.0%) | 0 (0.0%) | 0 (0.0%) | 2 (1.0%) | 0 (0%) |
| **Pack year history known, N (%)** | 228 (73.8%) | 98 (73.7%) | 35 (89.7%) | 34 (89.5%) | 159 (75.0%) | 6 (100%) |
| **Median (range)** | 30 (0 – 120) | 25 (0 – 100) | 30 (0 – 100) | 35 (0 – 90) | 30 (0 – 160) | 48 (0 – 125) |
| **Histology** |  |  |  |  |  |  |
| **Non-squamous, N (%)** | 290 (93.9%) | 122 (91.9%) | 33 (84.6%) | 32 (84.2%) | 201 (94.8%) | 5 (83.3%) |
| **Squamous, N (%)** | 11 (3.6%) | 7 (5.2%) | 1 (2.6%) | 2 (5.3%) | 6 (2.8%) | 0 (0%) |
| **Other, N (%)** | 8 (2.5%) | 4 (2.9%) | 5 (12.8%) | 4 (10.5%) | 5 (2.4%) | 1 (16.7%) |
| **Stage:** |  |  |  |  |  |  |
| **IIIB/C, N (%)** | 19 (6.1%) | 3 (2.3%) | 3 (7.7%) | 2 (5.3%) | 12 (5.7%) | 0 (0%) |
| **IV, N (%)** | 289 (93.5%) | 129 (97.0%) | 35 (89.7%) | 35 (92.1%) | 200 (94.3%) | 6 (100%) |
| **Stage III/IV, N (%)** | 1 (0.3%) | 1 (0.7%) | 1 (2.6%) | 1 (2.6%) | 0 (0%) | 0 (0%) |
| **PD-L1 Known, N (%)** | 264 (85.4%) | 99 (74.4%) | 34 (87.1%) | 30 (78.9%) | 174 (82.1%) | 6 (100%) |
| **Median (range)** | 39.0 (0 – 100) | 9.0 (0 – 100) | 7.5 (0 – 100) | 45.0 (0 – 100) | 5.0 (0 – 100) | 72.5% (0 – 100) |
| **TMB Known, N (%)** | 15 (4.9%) | 8 (6.0%) | 12 (30.0%) | 17 (44.7%) | 17 (8.0%) | 1 (16.7%) |
| **Median (range)** | 12.0 (3 – 32) | 4.6 (2 – 12) | 13.7 (3 – 37) | 11.0 (0 – 23) | 5.9 (0 – 44) | 7.5 (7.5 – 7.5) |

**Supplementary Table 2 - Treatment information for RAS-PM patients:** Percentages calculated by total number of patients in each KRAS subgroup, except for time to progression from start of immunotherapy, which is calculated divided by the total of patients who were treated with immunotherapy-based regimens.

| **Treatment characteristics** | **KRAS^G12C^** | **KRAS^G12D^** | **KRAS^G13C^** | **KRAS^G13D^** | **KRAS^G12^ others** | **KRAS^G13^ others** |
| --- | --- | --- | --- | --- | --- | --- |
| **No. Patients** | 309 | 133 | 39 | 38 | 212 | 6 |
| **1^st^ line treatment regimens:** | | | | |  |  |
| **CT-IO, N (%)** | 73 (23.6%) | 32 (24.0%) | 15 (38.5%) | 11 (29.0%) | 50 (23.6%) | 1 (16.7%) |
| **Platinum-doublet, N (%)** | 123 (39.8%) | 69 (51.9%) | 14 (35.8%) | 15 (39.4%) | 96 (45.3%) | 1 (16.7%) |
| **IO monotherapy, N (%)** | 87 (28.2%) | 22 (16.5%) | 8 (20.5%) | 10 (26.3%) | 54 (25.5%) | 4 (66.7%) |
| **Single agent CT, N (%)** | 7 (2.3%) | 5 (3.8%) | 1 (2.6%) | 0 (0.0%) | 2 (0.9%) | 0 (0%) |
| **Other therapies, N (%)** | 19 (6.1%) | 5 (3.8%) | 1 (2.6%) | 2 (5.3%) | 10 (4.7%) | 0 (0%) |
| **Immunotherapy at any line:** | | | | |  |  |
| **Yes, N (%)** | 239 (77.3%) | 93 (69.9%) | 30 (76.9%) | 30 (78.9%) | 175 (82.5%) | 6 (100%) |
| **1^st^ line, N (%)** | 166 (53.7%) | 55 (41.3%) | 24 (61.5%) | 22 (57.8%) | 107 (50.4%) | 5 (83.3%) |
| **2^nd^ line, N (%)** | 63 (20.4%) | 28 (21.1%) | 5 (12.8%) | 8 (21.1%) | 56 (26.4%) | 1 (16.7%) |
| **> 2^nd^ line, N (%)** | 10 (3.2%) | 10 (7.5%) | 1 (2.6%) | 0 (0.0%) | 12 (5.7%) | 0 (0%) |
| **Immunotherapy regimen:** |  |  |  |  |  |  |
| **Monotherapy, N (%)** | 154 (49.8%) | 57 (42.9%) | 13 (33.3%) | 18 (47.4%) | 118 (55.7%) | 5 (83.3%) |
| **Combined with CT, N (%)** | 76 (24.6%) | 33 (24.8%) | 17 (43.6%) | 11 (28.9%) | 52 (24.5%) | 1 (16.7%) |
| **Trial combinations, N (%)** | 9 (2.9%) | 3 (2.2%) | 0 (0.0%) | 1 (2.6%) | 5 (2.3%) | 0 (0%) |
| **Time to progression from start of immunotherapy:** | | | | |  |  |
| **≤ 6 months, N (%)** | 115 (48.2%) | 44 (47.3%) | 16 (53.3%) | 13 (43.3%) | 91 (52.0%) | 4 (66.7%) |
| **> 6 months, N (%)** | 123 (51.5%) | 49 (52.7%) | 14 (46.7%) | 17 (56.7%) | 83 (46.9%) | 2 (33.3%) |
| **Dates missing, N (%)** | 1 (0.3%) | 0 (0%) | 0 (0%) | 0 (0%) | 1 (1.1%) | 0 (0%) |

**Supplementary Table 3 - Number of patients tested for each co-mutation within RAS-PM, independently of treatment regimen**

| **Co-mutation testing** | **Number tested** | **Mutant** | **Wildtype** | **Not tested** | **% of mutant** | **P value** |
| --- | --- | --- | --- | --- | --- | --- |
| **TP53:** |  |  |  |  |  |  |
| **G12X** | 337 | 147 | 190 | 317 | 43.60% | **0.048** |
| **G13X** | 63 | 36 | 27 | 20 | 57.10% |  |
| **G12C** | 134 | 67 | 67 | 175 | 50.00% | 0.585 |
| **G13C** | 26 | 13 | 13 | 13 | 50.00% |  |
| **G12D** | 85 | 33 | 52 | 48 | 38.80% | **0.007** |
| **G13D** | 33 | 22 | 11 | 6 | 66.60% |  |
| **STK11:** |  |  |  |  |  |  |
| **G12X** | 350 | 91 | 259 | 304 | 26.00% | 0.076 |
| **G13X** | 64 | 10 | 54 | 19 | 15.60% |  |
| **G12C** | 149 | 45 | 104 | 160 | 30.20% | 0.072 |
| **G13C** | 24 | 3 | 21 | 15 | 12.50% |  |
| **G12D** | 79 | 16 | 63 | 54 | 20.30% | 0.413 |
| **G13D** | 36 | 5 | 31 | 2 | 13.90% |  |
| **NF1:** |  |  |  |  |  |  |
| **G12X** | 296 | 14 | 282 | 358 | 4.70% | **< 0.001** |
| **G13X** | 62 | 14 | 48 | 21 | 22.60% |  |
| **G12C** | 123 | 10 | 113 | 186 | 8.10% | 0.193 |
| **G13C** | 24 | 4 | 20 | 14 | 16.70% |  |
| **G12D** | 67 | 1 | 66 | 66 | 1.50% | **< 0.001** |
| **G13D** | 34 | 10 | 24 | 4 | 29.40% |  |
| **KEAP1:** |  |  |  |  |  |  |
| **G12X** | 238 | 23 | 215 | 416 | 9.70% | 0.182 |
| **G13X** | 57 | 9 | 48 | 26 | 15.80% |  |
| **G12C** | 89 | 7 | 82 | 220 | 7.90% | 0.171 |
| **G13C** | 23 | 4 | 19 | 16 | 17.40% |  |
| **G12D** | 58 | 5 | 53 | 75 | 8.60% | 0.831 |
| **G13D** | 30 | 3 | 27 | 8 | 10.00% |  |
| **BRAF:** |  |  |  |  |  |  |
| **G12X** | 634 | 7 | 627 | 20 | 1.10% | 0.067 |
| **G13X** | 83 | 3 | 80 | 0 | 3.60% |  |
| **G12C** | 300 | 4 | 296 | 9 | 1.30% | 0.091 |
| **G13C** | 39 | 2 | 37 | 0 | 5.10% |  |
| **G12D** | 127 | 1 | 126 | 6 | 0.80% | 0.362 |
| **G13D** | 38 | 1 | 37 | 0 | 2.60% |  |

**Supplementary Table 4 – Antibodies**

| **Antibody** | **Source** | **Catalogue number** | **RRID** | **Application and dilution** |
| --- | --- | --- | --- | --- |
| **KRAS mouse antibody** | LS-Bio | LS-C175665 | AB_3713472 | Western blotting – 1:1000 |
| **ERK1/2 rabbit antibody** | Cell Signaling | 4695 | AB_390779 | Western blotting – 1:1000 |
| **p-ERK1/2 (Thr202/Tyr204) rabbit antibody** | Cell Signaling | 4370 | AB_2315112 | Western blotting – 1:1000 |
| **p-AKT (Ser473) rabbit antibody** | Cell Signaling | 4060 | AB_2315049 | Western blotting – 1:1000 |
| **AKT rabbit antibody** | Cell Signaling | 9272 | AB_329827 | Western blotting – 1:1000 |
| **S6 Ribosomal Protein rabbit antibody** | Cell Signaling | 2217 | AB_331355 | Western blotting – 1:1000 |
| **p-S6 (Ser235/236) Ribosomal Protein rabbit antibody** | Cell Signaling | 4858 | AB_916156 | Western blotting – 1:1000 |
| **APC-conjugated CD44 antibobdy** | Biolegend | 103012 | AB_312963 | Flow cytometry – 1:100 |
| **APC Rat IgG2b, κ Isotype Ctrl Antibody** | Biolegend | 400612 | AB_326556 | Flow cytometry – 1:100 |
| **Mouse HRP-conjugated secondary antibody** | Cell Signaling | 7076S | AB_330924 | Western blotting – 1:2000 |
| **Rabbit HRP-conjugated secondary antibody** | Cell Signaling | 7074S | AB_2099233 | Western blotting – 1:2000 |
| **β-actin mouse antibody** | Sigma | A5441 | AB_476744 | Western blotting – 1:1000 |
| **Vinculin mouse antibody** | Abcam | AB91459 | AB_2050446 | Western blotting – 1:1000 |
| **4E-BP1 rabbit antibody** | Cell Signaling | 9644 | AB_2097841 | Western blotting – 1:1000 |
| **p-4E-BP1 (Ser65) rabbit antibody** | Cell Signaling | 9451 | AB_330947 | Western blotting – 1:1000 |
| **α-tubulin mouse antibody** | Novus Biologicals | NB-100 690 | AB_2210209 | Western blotting – 1:1000 |
| **LKB1 rabbit antibody** | Cell Signaling | 3047 | AB_2198327 | Western blotting – 1:1000 |
| **P-P70 S6K (Thr389) rabbit antibody** | Cell Signaling | 9205 | AB_330944 | Western blotting – 1:1000 |
| **P70 S6K rabbit antibody** | Cell Signaling | 2708 | AB_390722 | Western blotting – 1:1000 |
| **cMyc rabbit antibody** | Cell Signaling | 9402 | AB_2151827 | Western blotting – 1:1000 |
| **Cyclin D1 rabbit antibody** | Cell Signaling | 2978 | AB_2259616 | Western blotting – 1:1000 |
| **RAS G12D rabbit antibody** | Cell Signaling | 14429 | AB_2728748 | Western blotting – 1:1000 |
| **Neurofibromin 1 (NF1) rabbit antibody** | Cell Signaling | 14623 | AB_2798543 | Western blotting – 1:1000 |
